# Supplementary material for: ATTIRE: Albumin To prevenT Infection in chronic liveR failurE: study protocol for an interventional randomised controlled trial
Source: BMJ Open. 2018 Oct 21;8(10):e023754. doi: 10.1136/bmjopen-2018-023754 (PMC6196858; doi:10.1136/bmjopen-2018-023754)
Supplement: Supplementary file 1 [file bmjopen-2018-023754supp001.pdf]

Appendix 1 – Classification of infection

1. Spontaneous bacteraemia: positive blood cultures without a source of infection.
2. SBP: ascitic fluid polymorphonuclear cells >250 cells/mm^3
3. Lower respiratory tract infections: new pulmonary infiltrates in the presence of:
    i) at least one respiratory symptom (cough, sputum production, dyspnoea, pleuritic pain) with
    ii) at least one finding on auscultation (rales or crepitation) or one sign of infection (core body temperature >38°C or less than 36°C, shivering, or leukocyte count >10,000/mm3 or <4,000/mm3) in the absence of antibiotics.
4. Clostridium difficile Infection: diarrhoea with a positive C. difficile assay.
5. Bacterial entero-colitis: diarrhoea or dysentery with a positive stool culture for Salmonella, Shigella, Yersinia, Campylobacter,or pathogenic E. coli.
6. Soft-tissue/skin Infection: fever with cellulitis.
7. Urinary tract infection (UTI): urine white blood cell >15/high-power field with either positive urine gram stain or culture.
8. Intra-abdominal infections: diverticulitis, appendicitis, cholangitis, etc.
9. Other infections not covered above.
10. Fungal infections as a separate category.
