# Supplementary material for: ATTIRE: Albumin To prevenT Infection in chronic liveR failurE: study protocol for an interventional randomised controlled trial
Source: BMJ Open. 2018 Oct 21;8(10):e023754. doi: 10.1136/bmjopen-2018-023754 (PMC6196858; doi:10.1136/bmjopen-2018-023754)
Supplement: Supplementary file 2 [file bmjopen-2018-023754supp002.pdf]

Appendix 2 - Exploratory Objectives:

1. Effect of 20% HAS on the incidence, morbidity and mortality of the following infections:
    a. Bacterial
        i. Community acquired (diagnosed within 48 hours of admission in patients without previous hospitalisation in the last 6 months)
        ii. Nosocomial
        iii. Drug resistant pathogens
        iv. Culture negative sepsis (patients treated with antibiotics in the absence of microbiology evidence of infection)
        v. Clostridium Difficile infection
        vi. Source of infection (e.g. bacteraemia, respiratory, peritonitis, urine, skin etc.)
    b. Fungal
    c. Viral
2. Incidence, morbidity and mortality according to aetiology of Cirrhosis
3. Outcome for participants admitted to hospital for the first time with decompensated liver cirrhosis compared with participants who have previously been admitted to hospital with decompensated liver cirrhosis.
4. Requirement for ICU use of inotropes, vasopressors, hemofiltration and intubation and ventilation.
5. Analysis of samples from patients:
    a. Immune function studies using plasma samples – including but not limited to cell culture, qPCR, Albumin-ligand binding studies, western blot, flow cytometry and immunohistochemistry
    b. Plasma and urine lipidomic profile (in participants giving optional consent for urine samples)
6. Analysis of samples from patients providing additional optional consent (subject to further application for funding and ethical approval) :
    a. Stool microbiome analysis
    b. Genetic analysis of host response to infection
    c. Pathogen detection
