# Supplementary material for: ATTIRE: Albumin To prevenT Infection in chronic liveR failurE: study protocol for an interventional randomised controlled trial
Source: BMJ Open. 2018 Oct 21;8(10):e023754. doi: 10.1136/bmjopen-2018-023754 (PMC6196858; doi:10.1136/bmjopen-2018-023754)
Supplement: Supplementary file 3 [file bmjopen-2018-023754supp003.pdf]

Appendix 3: ATTIRE Patient Information Sheet (PIS)

ATTIRE: Stage 2

Albumin **T**o preven**T** **I**nfection in chronic live**R** failur**E**

The Problem

The most common cause of death in patients with liver disease is infection

➔ this is because in liver disease the immune system does not work well, especially when you are unwell in hospital.

What we know

Albumin (a protein found in your blood) is lower in patients with liver disease. Research looking at blood samples has suggested that increasing the albumin levels in the blood can potentially improve the immune system in patients with chronic liver disease.

What question is this study trying to answer?

1. Does albumin actually stop patients admitted to hospital with chronic liver disease from getting infections? (or help them fight them)

What additional treatment or investigations may occur if I take part?

If you decide to take part, you will be assigned to either the 'albumin group' or the 'standard of care group'. This allocation will be random.

1. In the 'standard of care group', you will receive exactly the same treatment as you would have done had you not been in the trial.
2. If in the 'albumin group' you will be given an intravenous infusion of albumin every day whilst you are in hospital **and** you will also receive all the other treatment that you would have done had you not been in the trial. Albumin is a very safe protein infusion that is already commonly used in liver patients who have kidney problems or ascites (fluid in the abdomen).
3. We will collect some blood samples (3 times) during your admission and if you are happy we will also collect urine (2) and stool sample (1). Where possible the blood will be taken when your other blood tests collection occurs to avoid any additional tests.

Throughout the trial you will be asked to complete some questionnaires; this will start when you enter the study and at 3 and 6 months after you are discharged from hospital. If you are happy we will collect one blood sample at either of these appointments.

What are the possible advantages?

You may not have any direct benefit from taking part in the study but you will be a part of a large study that will help to decide whether this is a beneficial treatment for patients with liver disease to help decrease infection.

What are the possible disadvantages?

You may be connected to a drip for 1-2 hours a day (this can be when other drip medication is given). The side effects of albumin are very rare (listed in detail in the following pages).

ATTIRE

Stage 2: Patient Information Sheet

Full study title:

Albumin **T**o preven**T** **I**nfection in chronic live**R** failur**E**.

A trial to investigate whether giving albumin to patients with advanced liver cirrhosis will reverse immune suppression and prevent infection.

Invitation to take part in research study

We would like to invite you to take part in a research trial. Before you decide we would like you to understand why the research is being done and what it involves. Please read this information sheet; one of the study team will answer any questions you may have. Talk to others about the study and ask us if there is anything that is not clear. Take time to decide whether or not you wish to take part.

The ATTIRE study is in two stages. The first stage included 80 patients and the second includes over 860 patients. They are very similar, but will look at slightly different outcomes.

This Patient Information Sheet is for Stage 2.

What is the purpose of the study?

Liver cirrhosis results in the liver tissue becoming scarred and developing lumps, these changes can prevent the liver from functioning properly. As the liver function worsens, symptoms such as fluid within the abdomen or legs, yellow skin, vomiting blood and confusion can develop. These are a result of advanced liver disease and are termed complications of cirrhosis. There are about 60,000 patient admissions to hospitals each year with such complications of liver cirrhosis. These patients are at higher risk of developing an infection during their hospital stay and this increase in risk is due to a weakened immune system. These infections can be very serious.

Giving fluid directly into the veins (intravenous) is known to be very important to help kidney function in patients with advanced liver disease. However, how much to give or indeed what is the best fluid to give is currently unknown. We have shown in laboratory testing, that albumin might be the best option as it can strengthen the immune system. However, it is unclear whether this will reduce the number of infections in hospital and that is what our trial wishes to investigate.

Albumin is found in the blood and is made in the liver. Patients with liver cirrhosis cannot make albumin as easily due to the damage to their liver. Albumin is already used to treat patients with liver cirrhosis and is known to be safe. In this stage of the study, patients will be given extra albumin to see if this helps their immune system to fight infections.

Why have I been chosen?

You are being asked to take part in this study as you have been admitted to hospital with complications of liver cirrhosis. Your doctor believes that you could be eligible to take part.

Do I have to take part?

It is up to you whether or not to take part in the study. If you decide not to take part, this will not affect the treatment you would normally receive. If you take part, you are free to withdraw at any time without giving a reason; this will not affect the care you receive.

What will happen to me if I take part?

If you take part in this study you will be either randomly allocated to the 'albumin group' or the 'standard of care group'.
If you are allocated to the 'albumin group', you will be given albumin solution as an intravenous fluid. Albumin has been shown to be safe in patients with liver disease. The amount of albumin that you are given will be decided by the amount that you already have in your blood. You will receive an infusion each day that you are in hospital, for a maximum of 14 days, until the levels in your blood have reached normal. You will also receive all of the other treatment that you would normally have had you not been in the study.
If you are in the 'standard of care group' you will have the treatment you would normally have, had you not been a part of the study, this may include albumin if you need it for another reason.
Regardless of which group you are in, you will have blood tests on most days while you are in hospital (as part of your normal care). At this time an additional sample (about 1 teaspoon) will be collected for up to 4 days during your time in the trial. You will also be asked to provide stool and urine samples while in the study, however this sample collection is optional and you can still take part in the study without providing these samples.
You also will be asked to complete some patient questionnaires during your time in the trial.
You will be contacted at 3 months and 6 months following your discharge from hospital to attend hospital visits, we will try to have these visits at the same time as your regular clinic appointments if possible.

What will I have to do?
If you agree to take part in the study you will be asked to sign a consent form. A member of the study team will then go through the initial assessments required for the study including collecting details of your medical history and any medication that you are currently taking. You will have tests (which will be part of your standard care) to look at your general health (e.g. heart rate and blood pressure), whether you have an infection, your liver function and the level of albumin already in your blood. Only if the level of albumin is below a certain level, will you be able to take part.

A research nurse will then come and record your blood tests and how well you are every day whilst you are in hospital. When you are discharged from hospital the research nurse will arrange to see you 3 months and 6 months after discharge to ask you some questions about how well you are feeling, this will be at the same time as your other clinic appointments if possible.

If you are a woman of child bearing potential, a pregnancy test will be carried out.

<u>Starting Treatment</u>

Once all of your tests have been completed and if you are still eligible to participate, a computer outside of the hospital will decide whether you will be in the 'albumin group' or the 'standard of care group', this is called randomisation. You will get exactly the same hospital care as you would have previously but with (or without) additional albumin infusions. This is so the trial investigators can see if there is an advantage, or disadvantage, of being given albumin.

The test for the amount of albumin in your blood will be used to work out how much albumin will be given, if you are in the 'albumin group', this will be given by a nurse or doctor. We expect the test to be performed anyway and so will still be done if you are in the 'standard of care group' as well. The albumin will be given directly into your blood through an intravenous cannula (a tube inserted into your vein to give fluid); the cannula will already be present as part of your standard care, an extra one will *not* be required. This will happen each day that you are on the study (a maximum of 14 days).

You will have blood tests on most days for liver function and to look for infections. These would normally be taken during your hospital stay. An extra blood sample (1 teaspoon) will be taken at the same time on three days throughout your admission while in the study and will be used to look at your immune system. You will also be asked to provide urine and stool samples whilst in the study, however these will be optional

samples. The stool samples and any samples left over will be stored for future research (for which further ethical committee review will be required).
If you do get an infection whilst in the study, you will be given the normal treatment for that type of infection that may include albumin. Taking part in the study will not affect the care that you normally get from your doctor.

<u>End of study treatment</u>
When you have taken part in the study for 14 days or your when your doctor feels you are ready to be discharged (if this is before 14 days of being on the study), you will stop your study treatments. This means that you will no longer be given albumin as part of the study or have any samples collected that are not part of your standard treatment. You will be asked to complete a questionnaire when you are discharged.
If you doctor feels that you still need albumin, you will continue to receive it.

The study team will then also contact you at 3 months and 6 months after you have been discharged from hospital, this will be at the same time as your other clinic appointments if possible. At these visits you will undergo tests that you would normally have as part of your standard of care and you will be asked to complete some questionnaires to see how you are feeling and what treatment you have had since being discharged from hospital. If you agree an extra blood sample (1 teaspoon, as above) will be taken at the same time as one of these appointments.

At the end of the study, if you agree, we will access information about you that is held by NHS digital, the Office of National Statistics (ONS) and other central NHS bodies regarding hospital admissions and mortality statistics for the six months following treatment. This is data that is collected as part of standard practice and will involve no additional involvement by you. The data, which is highly regulated, will be used to see if there are any long term effects of the treatment for you and the services that you may access.

When should I contact the study team?
You should contact a member of the study team or a doctor or nurse on your ward if you feel unwell at any time or have any incidents that affect your physical wellbeing. Contact details of the study team at your hospital and the Chief Investigator are available at the end of this leaflet.

What happens if I become pregnant?
All women of child-bearing potential will have a pregnancy test before entering the study. If you do become pregnant during treatment, you should inform your study team. It is not thought that albumin will affect pregnancy.

What are the alternative treatments if I don't take part?
If you do not take part in the study, standard treatment will be offered, which may include the use of albumin. Albumin is currently used for several reasons in patients with liver disease for example those who require extra fluid to support their kidneys, or to replace fluid that has been drained from the abdomen, or in those who develop infections.

What are the possible disadvantages and risks of taking part?
There are some possible risks and disadvantages that you should consider before taking part in this study:

- Wherever possible we will ensure that the blood tests needed for the study are taken at the same time as those needed for your routine care, but you may be asked to have additional samples taken if this is not possible. The risks of having a blood test include local bruising and discomfort.
- You may experience discomfort and bruising from fluid infusion.
- As the albumin is taken from human blood, in theory there is chance that you could get an infection (virus) from the person who has donated the blood. However there are no reports of patients getting viral infections from albumin that is made following European manufacturing standards. The albumin

given as part of the study is the same as that which would be given to you as part of your regular care by your doctor which you may have received as a treatment in the past.

- A cannula (very small plastic tube) will be used to give you the albumin. We would expect that you already will have one of these to give your other medications whilst in hospital and will use the same cannula. However if you don't have any medications or fluid that need to be given into the vein we will need to place a small cannula into the vein in order to give you the albumin.
- If in the albumin group you will be connected to an albumin infusion for around 1-2 hours a day. You can still move around the ward with this but will need to have a drip stand with you.

What are the possible benefits of taking part?
There may be no benefit to you from taking part, however, intravenous albumin may help your kidneys and raise blood pressure that can be low in patients with liver problems. Taking part may also provide additional information about patients with liver disease.

What are the side effects of any treatment received when taking part?
The side effects of albumin are rare but can include the following:

	**Very Common**	**Common**	**Uncommon**	**Rare**	**Very Rare**
Immune system disorders					Anaphylactic shock
Gastrointestinal disorders				Nausea	
Skin and subcutaneous tissue disorders				Flushing, skin rash	
General disorders and administration site conditions				Fever	

*__Very Common:__ in more than 1 in 10 patients treated; **Common:** in less than 1 in 10, but more than 1 in 100 patients treated; **Uncommon:** in less than 1 in 100, but more than 1 in 1000 patients; **Rare:** in less than 1 in 1000, but more than 1 in 10000 patients treated; **Very rare:** in less than 1 in 10000 patients treated, including isolated cases

Other side effects observed after placing Human Albumin on the market are: Hypersensitivity/Allergic reactions, Headache, Rapid heart beat, Abnormally low blood pressure, Breathlessness or breathing discomfort, Vomiting, Altered sense of taste, Hives, Itchiness, Chills, Heart attack, Irregular heart beat, Accumulation of fluid in the lung.

If any of the above reactions occur these will be treated as they would in normal standard care.

What if relevant new information becomes available?
Sometimes we get new information about the treatments being studied. If this happens, your study team will tell you about it and discuss how this may impact you. They will discuss whether you want to or should continue in the study.

What happens when the research study stops?
If you still require any treatment once the study stops, your doctor will prescribe the treatment most suitable for you.

Expenses and payments
You will not receive any payments for participating in this study.

What if something goes wrong?
If you have any concerns about the study, you should discuss them with your local study team first. You can also contact the Chief Investigator for the study, Dr Alastair O'Brien, on 020 7679 6851 who will be happy to discuss any of your concerns.

If you would like to discuss your concerns with someone not involved in the study, please contact your local Patient Advice and Liaison Service (PALS). Information on how to contact your local PALS can be found at the end of this information sheet.

Every care will be taken during this clinical study. However, in the unlikely event that you are injured by taking part, compensation may be available. If you suspect that the injury is the result of the Sponsor's (UCL) or the hospital's negligence then you may be able to claim compensation. After discussing it with your study team, please write to Dr Alastair O'Brien who is the Chief Investigator for the clinical study, about details of your claim. The Chief Investigator will then pass the claim to the Sponsor's Insurers, via the Sponsor's office. You may have to bear the costs of the legal action initially, and you should consult a lawyer about this. Contact details of the study team at your hospital and the Chief Investigator are available at the end of this leaflet.

You may also be able to claim compensation for injury caused by participation in this clinical study without the need to prove negligence on the part of University College London or another party. You should discuss this possibility with your study team in the same way as above.

Regardless of this, if you wish to complain, or have any concerns about any aspect of the way you have been approached or treated by members of staff or about any side effects (adverse events) you may have experienced due to your participation in the clinical study, the normal National Health Service complaints mechanisms are available to you. Please ask your study team if you would like more information on this. Details can also be obtained from the Department of Health website: http://www.dh.gov.uk.

Will my taking part in this study be kept confidential?
Yes, it will. We will request your approval to contact and inform your GP in the consent form about your participation in the study. We will send information relating to this research to the Comprehensive Clinical Trials Unit at University College London (UCL), where the study is being managed. Relevant information about you and all other patients in the study will be stored on password protected computers and in locked filing cabinets and will only be available to the staff working on the study. The information stored by the Comprehensive Clinical Trials Unit will contain a unique code, your initials, and month and year of birth. Your full name, address, contact details relevant medical history and relevant test results will be maintained at your local hospital. The information may be accessed by authorised personnel from UCL and the NHS hospital where you are being treated for auditing and monitoring purposes or by the regulatory authorities for inspection purposes to make sure the study is being carried out properly. Data collected during the study may be sent pseudo-anonymously (partially linked) to associated researchers in countries where the laws don't protect your privacy to the same extent as the law in the European Economic Area (EEA) but we will take all necessary steps to protect your privacy. Any data sent will only contain your unique code, your initials and month and year of birth. A data protection officer at UCL will be informed about any potential data transfer. You will be asked whether you agree to this when you sign the consent form.

If you withdraw consent from further study treatment, unless you object, your data and samples will remain on file and will be included in the final study analysis. In line with Good Clinical Practice guidelines, at the end of the study, your data will be securely archived for a minimum of 5 years. Arrangements for confidential destruction will then be made.

What will happen to the results of the research study?

If you choose to take part in the study, you will be given a unique trial identification code. All information collected from you for the study will be associated with this unique code, your initials, and month and year of birth. No other personal information will be sent to personnel outside your hospital and no one outside your hospital care team will be able to identify you from this information.

The blood, urine and stool samples collected for this study will be sent to the University College London, where they will be stored securely with your study specific identification number, initials, and month and year of birth. The blood and urine samples will be analysed at UCL to look at your immune system. The stool samples will be stored securely at UCL to be used for future research. Any future research carried out on the samples will be subject to further ethical committee review. It may be possible that the samples, or part of the samples, will be sent to countries outside the EEA where the laws for data protection differ from those in the UK. The urine and stool samples are optional samples and will not affect your participation in the study.

The Comprehensive Clinical Trials Unit will analyse the data collected on the patients in this stage of the study after all 866 patients have completed their treatment. The results of the study will be presented at a scientific conference and will be published in a scientific journal which will be accessible to the public. None of the research participants will be identified in any report or publication. Should you wish to see the results, or the publication, please ask your study team.

Who is conducting and funding the research?

This study is being managed by the Comprehensive Clinical Trials Unit at the University College London. Funding for the research has come from the Department of Health and Wellcome Trust.

Who has reviewed the study?

All research in the NHS is reviewed by a Research Ethics Committee and an agency of the Department of Health called the MHRA (the Medicines and Healthcare products Regulatory Agency). Both of these groups have confirmed that they are content for the study to go ahead and are regularly updated on its progress by the Comprehensive Clinical Trials Unit.

Who to contact for further information?

You are encouraged to ask any questions you wish, before, during or after your treatment. If you have any questions about the study, please speak to a member of the study team. If you wish to read the research on which this study is based, please ask your study team. If you require any further information or have any concerns while taking part in the study please contact one of the following people at your study site:

Doctor

Name *add name of PI* Tel. Number: *add Tel. number*

Study Nurse

Name *add name* Tel. Number: *add Tel. number*

PALS Add local PALS contact details

Or contact the Chief Investigator for the study:
Dr Alastair O'Brien at University College London, Tel. Number: 020 7 679 6851

Before you sign the informed consent form, you should ask questions about anything that you do not understand. The study staff will answer any questions before, during and after the study.

Thank you for taking the time to read this information sheet.

To be printed on hospital headed paper

ATTIRE

Stage 2: Patient Informed Consent Form

Albumin **T**o preven**T** **I**nfection in chronic live**R** failur**E** (ATTIRE)

PATIENT ID: ☐☐☐-☐☐☐-☐☐ SITE: ☐☐☐

MONTH AND YEAR OF BIRTH: ☐☐☐-☐☐☐☐ PATIENT INITIALS: ☐☐☐

M M M - Y Y Y Y

This Informed Consent Form is intended for consenting patients into Stage 2 of the ATTIRE study.

Please insert your **initials** in the boxes to confirm consent:

	**Initials**
1. I confirm that I have read and understood the Patient Information Sheet version 5.0 dated 15Jan2018 for the ATTIRE study and have had the opportunity to consider the information and ask questions, which have been answered to my satisfaction.	
2. I understand that my participation is voluntary and that I am free to withdraw at any time, without giving a reason and without my medical care or legal rights being affected.	
3. I understand that sections of my medical records may be looked at by properly authorised personnel involved in the running of the study or from regulatory authorities where it is relevant to my taking part in this study. I give permission for these individuals to have access to my records.	
4. I agree to provide blood samples.	
5. I agree to take part in the ATTIRE study.	
6. I agree to be contacted by the research team 3 and 6 months after my hospital discharge.	

Optional Consent:

The following are optional. Declining to any of the below will not prevent you from taking part in the trial. Please initial in the boxes to confirm consent:	Initials
7. I give permission for my study data to be sent to an associated researcher outside of the EEA.	
8. I agree to provide urine samples.	
9. I agree to provide a stool sample.	
10. I agree to my remaining samples, not used in this study, being used in future, ethically approved, research.	
11. I understand that the information held and maintained by NHS Digital, the Office of National Statistics and other central UK NHS bodies may be used to provide information about my health.	
12. I give permission for my GP to be informed about my participation.	

Patient/ Legal Representative's Name (score through as applicable)	Signature	Date
Person Taking Consent	Signature	Date
Witness (if applicable)	Signature	Date
