# Supplementary material for: ATTIRE: Albumin To prevenT Infection in chronic liveR failurE: study protocol for an interventional randomised controlled trial
Source: BMJ Open. 2018 Oct 21;8(10):e023754. doi: 10.1136/bmjopen-2018-023754 (PMC6196858; doi:10.1136/bmjopen-2018-023754)
Supplement: Supplementary file 4 [file bmjopen-2018-023754supp004.pdf]

Appendix 4

	***Screening/* Day 1 (Randomisation)** *(within 72hrs of admission)*	***Treatment Period*** *(Day 2- Day 14)*	***End of Treatment***	***Discharge***	***Follow up*** *(3 & 6 months +/- 1 month)*[d]
Eligibility screen	X				
Informed consent	X				
Medical history	X				
Blood tests and sample collection*	X	X	X		X
Clinical observations recorded	X	X	X		
Randomisation	X				
Dosing with HAS/Standard of care	X[b]	X			
Infection/new (change) in antibiotics	X	X	X		
Patient location recorded	X	X	X	X	X
Concomitant Medication	X	X	X	X	X
Pregnancy test (βhCG) (women of child bearing potential only)	X				
Survival		X	X		X
CSRI Questionnaire	X[c]				X
EQ-5D-5L Questionnaire	X			X	X

*Blood test include: FBC, U&ES, LFTs, CRP, INR, Serum Albumin concentrations. From patients who provide additional consent blood will be collected at randomisation (day 1) and on days 5 and 10 +/- 2 days. Urine sample will be collected on day 1 and day 5 +/- 2 days and A stool sample will be collected once during the patients treatment period. A blood sample from patients who consent will be taken at either the 3 or 6 month follow up visit.

[b] If randomised to receive the HAS treatment the volume of HAS given will be determined by the Serum Albumin results (see section 6.4.1.2)

[c] The CRSI should be completed before randomisation. If this is not possible, the questionnaire should be completed as soon as the patient is able, prior to discharge. The questionnaire should be completed in relation to the three months prior to admission – details of the current hospital admission should not be included.

[d] If the patient has been re-randomised into the study, before completing the follow up visits for the previous randomisation; the patient should not undergo the follow up activities relating to that randomisation The follow up visits should only be conducted for the current randomisation.
