# Supplementary material for: ATTIRE: Albumin To prevenT Infection in chronic liveR failurE: study protocol for an interventional randomised controlled trial
Source: BMJ Open. 2018 Oct 21;8(10):e023754. doi: 10.1136/bmjopen-2018-023754 (PMC6196858; doi:10.1136/bmjopen-2018-023754)
Supplement: Author's manuscript [file bmjopen-2018-023754.draft_revisions.pdf]

BMJ Open is committed to open peer review. As part of this commitment we make the peer review history of every article we publish publicly available.

When an article is published we post the peer reviewers' comments and the authors' responses online. We also post the versions of the paper that were used during peer review. These are the versions that the peer review comments apply to.

The versions of the paper that follow are the versions that were submitted during the peer review process. They are not the versions of record or the final published versions. They should not be cited or distributed as the published version of this manuscript.

BMJ Open is an open access journal and the full, final, typeset and author-corrected version of record of the manuscript is available on our site with no access controls, subscription charges or pay-per-view fees (http://bmjopen.bmj.com).

If you have any questions on BMJ Open's open peer review process please email info.bmjopen@bmj.com

BMJ Open

ATTIRE: Albumin To prevenT Infection in chronic liveR failurE: Study Protocol for an Interventional Randomised Controlled Trial

Journal:	*BMJ Open*
Manuscript ID	bmjopen-2018-023754
Article Type:	Protocol
Date Submitted by the Author:	10-May-2018
Complete List of Authors:	China, Louise; UCL, Division of Medicine<br>Skene, Simon; University of Surrey, Surrey Clinical Trials Unit<br>Bennett, Kate; UCL, Comprehensive Clinical Trials Unit<br>Shabir, Zainib; UCL, Comprehensive Clinical Trials Unit<br>Hamilton, Roseanna; UCL, Comprehensive Clinical Trials Unit<br>Bevan, Scott; UCL, Comprehensive Clinical Trials Unit<br>Chandler, Torsten; UCL, Comprehensive Clinical Trials Unit<br>Maini, Alex; UCL, Division of Medicine<br>Becares, Natalia; UCL, Division of Medicine<br>Gilroy, Derek; UCL, Division of Medicine<br>Forrest, Ewan; NHS Greater Glasgow and Clyde, Glasgow Royal Infirmary<br>O'Brien, Alastair; UCL, Division of Medicine
Keywords:	Adult gastroenterology < GASTROENTEROLOGY, Hepatobiliary disease < GASTROENTEROLOGY, Gastroenterology < INTERNAL MEDICINE

For peer review only

SCHOLARONE™
Manuscripts

ATTIRE: Albumin To prevenT Infection in chronic liveR failurE: Study Protocol for an Interventional Randomised Controlled Trial

Louise China*[1], Simon S Skene[2], Kate Bennett[3], Zainib Shabir[3], Roseanna Hamilton[3], Scott Bevan[3], Torsten Chandler[3], Alexander A Maini[1], Natalia Becares[1], Derek Gilroy[1], Ewan Forrest[4], Alastair O'Brien[1]

[1] Division of Medicine, University College London, University Street, London
[2]School of Biosciences and Medicine, University of Surrey
[3] Comprehensive Clinical Trials Unit, UCL, Gower Street, London, WC1E 6BT
[4]Department of Gastroenterology, Glasgow Royal Infirmary, Castle Street, Glasgow G4 0SF

*Correspondence: louise.china@ucl.ac.uk
s.skene@surrey.ac.uk
k.bennett@ucl.ac.uk
z.shabir@ucl.ac.uk
roseanna.hamilton@ucl.ac.uk
a.maini@ucl.ac.uk
natalia.becares.13@ucl.ac.uk
d.gilroy@ucl.ac.uk
Ewan.Forrest@ggc.scot.nhs.uk
a.o'brien@ucl.ac.uk

For peer review only

Abstract

Introduction

Circulating Prostaglandin E_2 levels are elevated in acutely decompensated cirrhosis and have been shown to contribute to immune suppression. Albumin binds to and inactivates this immune-suppressive lipid mediator. Human Albumin Solution (HAS) could thus be repurposed as an immune restorative drug in these patients.

This is a phase III randomised controlled trial to verify whether targeting a serum albumin level of ≥35g/l in hospitalised patients with decompensated cirrhosis using repeated intravenous infusions of 20% HAS will reduce incidence of infection, renal dysfunction and mortality for the treatment period (maximum 14 days or discharge if <14 days) compared to standard medical care.

Methods and Analysis

ATTIRE stage 2 is a multicentre, open label, interventional randomised controlled trial. Patients with decompensated cirrhosis admitted to hospital with a serum albumin of <30g/L are eligible, subject to exclusion criteria. Patients randomised to intravenous HAS will have this administered, according to serum albumin levels, for up to 14 days or discharge. The infusion protocol aims to increase serum albumin to near normal levels.

The composite primary endpoint is: new infection, renal dysfunction or mortality within the trial treatment period. Secondary endpoints include mortality at up to 6months, incidence of other organ failures, cost effectiveness and QOL outcomes and time to liver transplant. The trial will recruit 866 patients at more than 30 sites across the United Kingdom.

Ethics & Dissemination

Research ethics approval was given by the London-Brent research ethics committee (ref: 15/LO/0104). The clinical trials authorisation was issued by the medicines and healthcare products regulatory agency (ref: 20363/0350/001-0001). The trial is registered with the European Medicines Agency (EudraCT 2014-002300-24) and has been adopted by the NIHR (ISRCTN 14174793). This manuscript refers to version 6.0 of the protocol. Results will be disseminated through peer reviewed journals and international conferences. Recruitment of the first participant occurred on 25 Jan 2016.

For peer review only

Strengths and limitations of this study

- This is the first prospective, interventional RCT with the primary aim of assessing the effects of **targeted** 20% Human Albumin Solution (HAS) infusions on infection, organ failure and death in hospitalised patients with decompensation of liver cirrhosis.
- Study design was optimised by a prior 80 patient feasibility study
- Cost effectiveness and quality of life assessment will review whether any outcomes are sustainable and feasible in a national health service setting
- The study is un-blinded to site investigators due to lack of agreement regarding the control infusion fluid and high cost required
- Patients in the control arm may receive 20% HAS as part of current international guidance as it would be unethical to withhold this in certain situations.

Introduction

Liver disease is an increasing cause of mortality currently in the UK and is the fifth most common cause of death[1]. These deaths are predicted to double over the next 20 years[2]. Mortality is mainly seen in the middle-aged group. Liver disease has surpassed lung and breast cancer as the leading cause of years of working life lost, and is set to overtake ischaemic heart disease within 2–3 years[3].

Patients with complications of liver failure secondary to liver cirrhosis are described as decompensated patients. They are highly prone to bacterial infection[4] secondary to immune dysfunction[5], with nosocomial (hospital-acquired) infection rates of 35% compared to five per cent in non-cirrhotic patients[6,7]. Of those who develop infection with organ dysfunction, 60-95% will die, often following prolonged intensive care unit admission[8]. There is, however, no medical strategy to restore immune competence.

It has been demonstrated that elevated circulating Prostaglandin E_2 (PGE_2) levels contribute to immune suppression in acutely decompensated patients[9]. The plasma protein albumin binds and catalyses the inactivation of PGE_2[10]. The binding capacity of endogenous albumin is known to be defective in cirrhosis[11,12] and improves following albumin infusions[13]. Albumin is synthesised in the liver and levels fall as the synthetic function of the liver declines in advanced cirrhosis. Therefore low albumin levels, with defective binding function, make PGE_2 more bioavailable. We found a serum albumin of < 30g/L predicted plasma induced macrophage dysfunction in a small cohort of decompensated cirrhosis patients[9] and this was reversed when albumin levels were increased to ≥30g/L. This finding has recently been validated in a multicentre clinical trial[13], however it is not known if these ex-vivo observations translate into an improvement in clinical outcomes.

We propose a novel strategy to repurpose 20% Human Albumin Solution (HAS) as an immune restorative drug in patients with decompensated liver cirrhosis with the aim of maintaining serum albumin at near normal levels.

The ATTIRE phase II single arm, multicentre feasibility trial[14] (n=80) verified that daily intravenous human albumin infusions restored serum albumin levels to near normal in

For peer review only - http://bmjopen.bmj.com/site/about/guidelines.xhtml

hospitalised decompensated cirrhosis patients, that it was safe and that there was physician equipoise prior to proceeding to a large RCT[15]. It has also allowed refinement of our definitions of RCT outcomes.

This phase III randomised controlled trial (RCT) (n=866) will assess the impact of 20% HAS treatment on the incidence of nosocomial infections, organ dysfunction and mortality in patients admitted to hospital with decompensation of liver cirrhosis.

Methods and Analysis

Primary Objective:

To verify whether raising and maintaining serum albumin levels ≥30g/L in patients admitted with decompensated cirrhosis using repeated 20% HAS infusions will reduce incidence of infection, renal dysfunction and mortality for the treatment period (maximum 14 days or discharge if <14 days) compared to standard medical care (figure 1). To raise and maintain serum albumin levels above 30g/L, 20% HAS will be prescribed and administered in the intervention arm if levels are below 35g/L.

Secondary Objectives are to assess the impact of the intervention on development of other organ dysfunction (cardiac, respiratory, brain and liver), cost effectiveness; quality of life and sustainability of any outcomes over a 6 month follow up period.

Trial Design

This is a multicentre, open label, randomized controlled trial in which patients will either be treated with 20% HAS to raise and maintain serum albumin above 30g/L or will continue to receive their usual standard of care treatment. 866 sequential patients admitted to 30 UK participating hospitals with a clinical diagnosis of cirrhosis and decompensation will be screened using the inclusion and exclusion criteria (Table 1). Decompensation includes: jaundice, ascites, hepatic encephalopathy, variceal bleeding, coagulopathy and hepatorenal syndrome.

Patient Inclusion Criteria	Patient Exclusion Criteria
All patients admitted to hospital with acute onset or worsening of complications of cirrhosis	Advanced hepatocellular carcinoma with life expectancy of less than 8 weeks
Over 18 years of age	Patients who will receive palliative treatment only during their hospital admission
Predicted hospital admission ≥ 5 days at trial enrolment, which must be within 72 hours of admission	Patients who are pregnant
Serum albumin <30g/l at screening	Known or suspected severe cardiac dysfunction
Documented informed consent to participate (or consent given by a legal representative)	Any clinical condition which the investigator considers would make the patient unsuitable for the trial
	The patient has been involved in a clinical trial of Investigational Medicinal Products (IMPs) within the previous 30 days that would impact on their participation in this study
	Trial investigators unable to identify the patient (by NHS number)

Table 1. Patient Selection Criteria

As advanced liver disease requires frequent hospital re-admissions, patients may be enrolled in the RCT more than once, with a 30 day "washout period" following discharge after the first enrolment, during which time they cannot be enrolled for a second time. This is to account for albumin's half-life of 18-21 days[16]. Patients re-entering the trial in this way will be re-randomised, so that each enrolment will be considered as an independent patient 'presentation'[17].

Clinical trial endpoints

Primary endpoint

A composite endpoint comprising incidence of infection, renal dysfunction and mortality within the treatment period (for a maximum of 14 days OR when the patient is considered fit for discharge if < 14 days).

The three components of the composite endpoint are:

1. New Infection: indicated by clinician diagnosis and clinical evidence provided on completed infection case report form (CRF). Blinded data from a clinically representative sample of patients will be scrutinised for the presence of infection by a Clinical Trial Endpoint Review Committee. The committee will contain microbiologists who will validate the presence of infection according to peer reviewed criteria[18] (Appendix 1).
2. Renal Dysfunction: a serum creatinine increase of ≥50% as compared to serum creatinine at randomisation OR the patient initiated on renal replacement support (either haemodialysis or haemofiltration) OR a rise in serum creatinine of ≥26.5 µmol/L within 48hours.
3. Mortality

Primary endpoint data will be recorded throughout the treatment period; however only contributing events captured on the treatment day 3 case report form (CRF) through to the day 15 CRF (the end of treatment examination day) will contribute to the primary outcome. This allows the biological effect of albumin to be established at least 24 hours following initiation of treatment.

If the participant is discharged or deemed medically fit for discharge prior to day 15, no further primary outcome data will be measured after this date, as this will signal the end of the participant's treatment period.

Secondary Endpoints:

1. Mortality at 28 days post randomisation and at 3 & 6 months post discharge
2. Time to outcome (first event of infection/renal dysfunction/death)
3. Incidence of respiratory, circulatory and cerebral dysfunction during the treatment period.
4. Incidence of liver transplant within six months of treatment
5. Total amount of HAS administered during the treatment period
6. Duration of hospital stay
7. Change in prognostic score (assessed by MELD) between randomisation and end of treatment period.
8. Days in ICU during treatment period
9. Incremental cost and cost-effectiveness up to 6 months post discharge
10. Impact on quality of life (QOL) up to 6 months post discharge
11. Safety and tolerability of HAS as indicated by Serious Adverse Events (SAEs)
12. Use of terlipressin for (a) renal dysfunction, (b) hypotension, (c) variceal bleeding.

For peer review only

Patients in the RCT will be followed up 3 and 6 months (+/- 1 month) following discharge from hospital. Exploratory objectives are listed in Appendix 2.

Patient population

This will include all patients admitted to hospital with the complications of decompensated liver cirrhosis and serum albumin < 30 g/L, aged over 18 years with anticipated hospital length of stay of 5 or more days at trial enrolment, which should be no later than 72 hours from admission. This is subject to exclusion criteria as detailed in table 1. The diagnosis of cirrhosis will be made by the clinical team as per standard UK practice and does not require liver biopsy or imaging.

Consent

Patient information sheets (Appendix 3) will be given to and discussed with potential patients before consent is sought. Informed consent will be obtained from each participant or his or her legal representative. Patients who lack mental capacity, for any reason, are not excluded from the trial. An important subgroup of patients will have hepatic encephalopathy and these patients may lack capacity to consent, however these patients may be amongst those that receive maximum benefit from the intervention[19-21]. In this case consent will be sought from an appropriate legal representative independent of the research team as per current UK clinical trials regulations[22].

Intervention

After randomisation (when serum albumin is <30g/L) patients will receive either daily dose of 20% HAS intravenously if their serum albumin level is less than 35g/L (at approximately 100mls/hour) or standard medical care (which may include 20% HAS infusions for indications listed in established guidelines only – see below) for a maximum of 14 days or discharge (if < 14 days). The volume of HAS each day will be determined by the patient's serum albumin level on that day (or the closest previous measurement if there are no results from that day available).

Table 2 shows the suggested dosing protocol for albumin administration in the treatment arm group. This is a suggested regimen and responsible clinicians are given flexibility to alter this depending on the clinical situation. The effectiveness of this protocol, and approach, was verified in the ATTIRE feasibility study[15]. Differing regimens may be used to cover large volume paracentesis (8g of albumin per litre of ascites drained) or treat Hepatorenal syndrome (1g of albumin per kilogram of body weight) as per international guidelines[23,24] but HAS *must* be prescribed and given if serum albumin <35g/L unless there are any safety concerns**.** All variations will be recorded in the patient's daily Case Report Form (CRF).

Patient's Serum Albumin Level	Amount of 20% HAS to be administered
≥35 g/L	none
30-34 g/L	100mls
26-29 g/L	200mls
20-25 g/L	300mls
<20 g/L	400mls

Table 2. Treatment arm dosing protocol for 20% HAS administration (amounts per day) as advised by measured serum albumin level on that day.

20% HAS will only be given in the Standard of Care arm if the patient requires large volume paracentesis or has SBP or HRS (as per established guidelines[24-26]). This needs to be clearly

For peer review only - http://bmjopen.bmj.com/site/about/guidelines.xhtml

recorded in the patient's CRF and if HAS is given for any other indication in the Standard of Care arm this will be considered a protocol deviation. The administration of HAS in the Standard of Care arm will be closely monitored by the Independent Data Monitoring Committee (IDMC).

Randomisation will use a minimisation algorithm incorporating a random element, stratifying by centre, MELD score, and number of organ dysfunctions, serum albumin level and if antibiotics are currently being prescribed. To ensure maximum balance is achieved across the stratification factors, minimisation will be carried out on these factors separately.

Evaluations during and after treatment

Clinical, biochemical, microbiological, health economic and quality of life data will be collected during the trial treatment period (see Appendix 4) using information from hospital notes that is recorded as standard of care. There is follow up at 3 and 6 months. The blood, urine and stool samples collected for immune function tests will be analysed in a blinded fashion at a central site.

Patient and Public Involvement

Patients who have had a previous diagnosis of liver cirrhosis were involved in the development of the research question and outcome measures. Patient feedback led to the addition of quality of life evaluation. The study protocol and patient information leaflets were reviewed by our patient representatives with positive feedback. In addition an independent patient representative sat on the ethics approval panel. Results will be disseminated to patients via open access publication and our local trials teams.

Statistical considerations

Sample Size

A 30% incidence of nosocomial infection in decompensated cirrhosis patients is well documented with up to 30% of these patients developing organ dysfunction[35] and an overall mortality of 38% at 1 month[36]. These figures are supportive of 30% as a conservative estimate for the composite primary endpoint of incidence of new infection, renal dysfunction or mortality up to 14 days from randomisation.

We have assumed that the "immune-restorative" albumin treatment would reduce this rate by 30% to a rate of 21%, which would be considered clinically relevant. 389 patients per arm would be sufficient to detect such a difference with 80% power at a significance level of 0.05. Allowing for loss-to-follow-up/withdrawal of 10% from the trial, we will aim to recruit 433 to each arm (866 in total).

Statistical Evaluation

A detailed statistical analysis plan (SAP) will be written and approved by the Trial Steering Committee before the substantive analysis of unblinded trial data.

Baseline characteristics will be summarised by treatment using appropriate descriptive statistics; means and standard deviations for approximately normally distributed variables, medians and interquartile ranges for non-normally distributed variables and counts (percentages) for categorical variables.

Primary outcome

The primary outcome is the difference in event rates, according to treatment, of the composite endpoint of infection, renal dysfunction and mortality within the intervention

For peer review only

period (from ≥ 24 hours from the start of treatment/randomisation up to a maximum of fourteen days or up to discharge if this is prior to fourteen days).

Since the primary outcome has a binary classification, logistic regression will be used to determine whether there is any difference in rates due to treatment, by inclusion of a binary covariate indicating treatment. The results will be adjusted for pre-determined prognostic factors used as stratifying variables in the randomisation, which shall be included as additional covariates in the model. The model coefficient due to treatment will give an estimate of the difference in log odds, or equivalently (exponentiated to give) an estimate of the odds multiplier, i.e. the change in odds of a negative outcome on the composite endpoint due to treatment with albumin. It is expected that this effect will be negative, so that treatment with albumin is seen to reduce the odds of a negative outcome. A reduction from 30% to 21% would be associated with a reduction in odds of around 38%. Predicted probabilities will be presented for the composite outcome for each of the treatment arms, adjusted for the model covariates.

The effect of treatment on the individual components, infection, renal dysfunction and mortality will be reported separately.

Secondary and exploratory outcomes

Secondary and exploratory outcomes will be handled similarly. Continuous secondary outcome measures will be analysed using a linear regression model, and time-to-event outcomes using Kaplan Meier plots and Cox's proportional hazards models where covariate adjustment is necessary.

All statistical tests will use a 2-sided p-value of 0.05, unless otherwise specified, and all confidence intervals presented will be 95% and two-sided. All statistical analysis will be performed using Stata (StataCorp, College Station, TX, USA).

Economic Evaluation

The economic evaluation will consist of two parts. The primary analysis will be a decision analytic model comparing the expected lifetime costs and benefits of treatment reported in terms of the cost per incremental quality adjusted life years (QALYs) gained. QALYs will be calculated based on the health related quality of life (HRQL) and mortality data collected during the trial. HRQL will be measured according to the EQ-5D-5L (www.euroqol.org), which we will collect at baseline, discharge, 3 months and 6 months (+/- 1 month) for each individual patient. If patients are re-randomised they will have separate follow up events for each episode of re-randomisation, unless the patient is re-randomised within the follow up phase of the study. Utility scores will be calculated using UK-specific tariffs and adjusting for baseline differences in patients in the trial arms if necessary. These will be extrapolated over the expected lifetime of the patient and where necessary will draw on the existing literature. The final form of the decision analytic model will be determined during the trial period. Appropriate methods for estimating changes in health over time, such as state-transition models, will be given consideration.

The secondary economic analysis will be a 'within-trial' analysis, reporting on the incremental cost and benefits observed during the trial period and follow-up periods. The cost-effectiveness measures in the within-trial analysis will be the incremental cost per infection avoided and the incremental cost per QALY gained. Costs will be measured as described below. Infections avoided will be derived from the primary outcome in the trial.

For peer review only - http://bmjopen.bmj.com/site/about/guidelines.xhtml

Patient resource use will be assessed using hospital patient records and a self-complete resource use form, the client services receipt inventory (CSRI). The volume of resource use for each cost component will be measured directly in the trial from patient records and using the CSRI. A detailed economic evaluation analysis plan will be developed for approval by the TSC.

Discussion

ATTIRE is a UK multicentre trial that aims to evaluate the re-purposing of Human Albumin Solution (HAS) as an immune restorative drug. This protocol describes the RCT which will determine if administering daily 20% HAS infusions in order to increase serum albumin to near normal levels after admission to hospital will reduced rates of infection, renal failure and death in patients with decompensation of liver cirrhosis.

In liver cirrhosis current evidence based guidance[24-26] advocates the use of HAS in large volume paracentesis[27], hepatorenal syndrome[28] and spontaneous bacterial peritonitis [29,30]. The mechanism behind current usage of albumin is that of volume expansion. However many hepatologists believe albumin has other medicinal properties however a large-scale interventional clinical trial is yet to support this hypothesis. Ex vivo analysis from the ATTIRE feasibility study supported an immune restorative role for albumin[13], however it is unknown whether this correlates with clinical outcome. Our inclusion criteria are broad and designed to represent the decompensated cirrhotic population admitted to UK hospitals in order to make trial outcomes highly relevant in an era of soaring liver disease with few available treatment options. Preventing a liver cirrhosis related infection could potentially provide a bridge to liver transplantation, which represents the only curative option for these patients.

The design of this RCT has been refined based on the outcomes of an 80 patient interventional feasibility study[15]. Our targeted infusion protocol (table 2) was effective at increasing serum albumin, even in patients with a very low starting serum albumin. Many hepatology trials focus solely on 28-day survival however mortality requires large sample size and may be caused by other factors unrelated to infection. We considered that endpoints for interventions to prevent infection should be assessed at an earlier stage during the patient admission using ward based clinical measures and laboratory data. Multiple components of a primary composite endpoint were tested in our feasibility study[15]. Defining infection according to clinician diagnosis, triggering completion of an infection case report form, with a substantial proportion blindly scrutinized by a microbiology panel worked well in our feasibility study. Patients that developed renal dysfunction as assessed by an increase in serum creatinine had a poor prognosis, as expected. However only one patient developed cerebral dysfunction reflecting severe hepatic encephalopathy ($\geq$grade 3) suggesting under reporting and the majority of patients that solely triggered respiratory and cardiovascular endpoints had a good outcome. This is counterintuitive as organ dysfunction is a key predictor of poor prognosis and we consider our data cast significant doubt over whether these dysfunctions can be recorded accurately in largely ward-based patients across multiple sites. This precludes their use as part of our RCT primary composite endpoint; although these will be reported.

Studies evaluating the safety of HAS infusions have generally shown it to be a safe treatment[27,31-33]. The main concerns in the cirrhotic population are related to volume overload leading to pulmonary oedema and increase in portal pressure leading to variceal bleeding. A recent interventional trial in septic decompensated patients reported an 8.3% rate of pulmonary oedema in the albumin treatment group[34]. However the weight based albumin dosing regimen on day 1 and 3 in this study led to much larger volumes of albumin

For peer review only - http://bmjopen.bmj.com/site/about/guidelines.xhtml

prescribed than suggested in our protocol for these days. This and other studies in cirrhosis have not reported an increased incidence in variceal bleeding [27,30,35].

A challenge this study faces is the lack of a comparator infusion fluid in the control arm and hence the impossibility of site investigator and patient blinding. We had therefore considered comparison with another fluid such 0.9% sodium chloride however, this posed a number of problems. Physician preference for resuscitation fluid varies substantially and in preliminary discussions some objected to the additional salt load associated with 0.9% sodium chloride, in the non-albumin arm, if the patient did not require fluid resuscitation. Administration of 20% HAS for SBP, LVP and HRS is standard of care and therefore if patients in either arm developed these complications during their admission they would have to be unblinded to see if they were already receiving 20% HAS as part of the trial. As a significant number of patients do develop these complications, the consequent number of unblinded patients could have rendered the process invalid. Therefore the RCT had to be an open label study. This risks biasing adverse event reporting in the treatment arm, making albumin appear unsafe. We therefore felt that an open-label trial design versus a comparator arm of current standard of care was the only pragmatic solution. It could be argued that any improved outcome in the albumin treatment arm may simply be due to additional fluid resuscitation. The mean volume of 20% HAS infused over the treatment period (14 days) was 1litre in our feasibility study.

The regulation of HAS administration in the standard of care (control) arm poses a challenge as HAS is routinely used in clinical practice based on evidence based guidelines[24-26].The protocol allows use for these indications. If HAS is administered to control arm patients outside of current evidence based guidance this will be defined as a protocol deviation and the IDMC will be monitoring these events closely.

The ATTIRE RCT (n=866, up to 40 centres) began recruitment in April 2016 and aims to complete in December 2019. The medicinal properties of albumin in the hepatological community have long been questioned. The outcomes of this large, multicentre, randomised control trial will be highly relevant to improving the care of hospitalised patients with decompensation of chronic liver disease.

Ethics

This research group will involve a potentially vulnerable patient group that have hepatic encephalopathy and therefore lack the capacity to consent. Patients with encephalopathy are at high risk of infection and could be those that potentially receive maximal benefit from the intervention and therefore should not be denied access to the trial treatment. We have undertaken steps to ensure these patients are appropriately recruited to the trial (described in 'Consent' section) and provided individual site training.

Research Ethics positive opinion was given by the London-Brent Research Ethics Committee (ref: 15/LO/0104) which specialise in trials involving patients who lack the capacity to consent.The Clinical Trials Authorisation was issued by the Medicines and Healthcare products Regulatory Agency (MHRA, ref: 20363/0350/001-0001). The trial is registered with the European Medicines Agency (EudraCT 2014-002300-24) and has been adopted by the NIHR. Recruitment commenced in April 2016 and will finish by December 2018.

For peer review only

Data Sharing

Primary and secondary outcomes will be published in a peer reviewed journal as an open access paper. De-indentified participant data will be shared, as part of the trial results. The full study protocol and statistical analysis plans can be requested by emailing attire@ucl.ac.uk

Trial funding and Sponsor

The work is supported by the Health Innovation Challenge fund (Wellcome Trust and Department of Health) award number 164699. The trial sponsor is UCL with trial management activities conducted by the UCL Comprehensive Clinical Trials Unit.

Trial management and monitoring

Research Steering Group

The Research Steering Group (RSG) operates on behalf of the funders to ensure that appropriate milestones have been met in the delivery of the trial. It consists the CI, an independent expert and representatives of the Welcome Trust and Department of Health.

Trial Management Group

The Trial Management Group (TMG) comprises the CI, Clinical Research Fellow, Clinical Project Manager, Trial Statistician, Trial Manager, Data Manager, Health Economist and five trial site PIs. The TMG is responsible for developing the design, co-ordination and strategic management of the trial.

Trial Steering Committee

The Trial Steering Committee (TSC) is the independent group responsible for oversight of the trial in order to safeguard the interests of trial patients. The TSC provides advice to the CI, CTU, funder and sponsor on all aspects of the trial through its independent chair.

Independent Data Monitoring Committee

The Independent Data Monitoring Committee (IDMC) is responsible for safeguarding the interests of trial patients, monitoring the accumulating data and making recommendations to the TSC on whether the trial should continue as planned. It comprises a clinical chair (independent Hepatologist), independent Gastroenterologist and an independent statistician all with expertise in Clinical Trials.

List of abbreviations used

RCT: Randomised Control Trial
HAS: Human Albumin Solution
CRF: Case Report Form
HRS: Hepatorenal Syndrome
SBP: Spontaneous Bacterial Peritonitis
CI: Chief Investigator
PI: Principle Investigator
MDM: monocyte derived macrophage
LPS: lipopolysaccharide
TNFα: Tumour necrosis factor alpha
ICU: Intensive Care Unit
IV: intravenous

Competing interests

None

Authors' contributions

For peer review only

All authors read and approved the final manuscript.
LC: protocol development, writing of this manuscript
ZS, SB, CT, RB: protocol development, protocol implementation, manuscript review
AM, NBS, DG: protocol development
SS: protocol development, statistical oversight, manuscript review
KB: protocol development, statistical input, manuscript review
AOB: concept and design, protocol development, writing of this manuscript

Acknowledgements
We have had the support of the following individuals via trial oversight committees:
Professor Graeme Alexander
Professor Stephen Brett
Professor Mauro Bernardi
Professor Dominique Valla
Dr Vipul Jaraith
Mr Tim Clayton
Mr Brennan Kahan
Dr James O'Beirne
Dr Jim Portal
Dr Gavin Wright
Dr Ewan Forrest
Dr Stephen Ryder
Dr Yiannis Kallis
Mr John Crookenden (Patient representative)
Mr Antonie Bakhuijsen (Patient representative)
Ms Susan Tebbs
A list of trial sites and PIs can be obtained by contacting attire@ucl.ac.uk

References

1. Health Service Quarterly. Vol. No. 40 (ed. Statistics, O.f.N.) p59-60 (Winter 2008).
2. Williams, R., *et al.* Addressing liver disease in the UK: a blueprint for attaining excellence in health care and reducing premature mortality from lifestyle issues of excess consumption of alcohol, obesity, and viral hepatitis. *The Lancet* **384**, 1953-1997 (2014).
3. Williams, R., *et al.* Disease burden and costs from excess alcohol consumption, obesity, and viral hepatitis: fourth report of the Lancet Standing Commission on Liver Disease in the UK. *Lancet* **391**, 1097-1107 (2018).
4. Fernandez, J., *et al.* Bacterial infections in cirrhosis: epidemiological changes with invasive procedures and norfloxacin prophylaxis. *Hepatology* **35**, 140-148 (2002).
5. Rajkovic, I. & Williams, R. Abnormalities of neutrophil phagocytosis, intracellular killing and metabolic activity in alcoholic cirrhosis and hepatitis. *Hepatology* **6**, 252-262 (1986).
6. Acevedo, J. & Fernandez, J. New determinants of prognosis in bacterial infections in cirrhosis. *World journal of gastroenterology : WJG* **20**, 7252-7259 (2014).
7. Fernandez, J., *et al.* Prevalence and risk factors of infections by multiresistant bacteria in cirrhosis: a prospective study. *Hepatology* **55**, 1551-1561 (2012).
8. O'Brien, A.J., Welch, C.A., Singer, M. & Harrison, D.A. Prevalence and outcome of cirrhosis patients admitted to UK intensive care: a comparison against dialysis-dependent chronic renal failure patients. *Intensive care medicine* **38**, 991-1000 (2012).

9. O'Brien, A.J.*, et al.* Immunosuppression in acutely decompensated cirrhosis is mediated by prostaglandin E2. *Nature medicine* **20**, 518-523 (2014).
10. Yang, J., Petersen, C.E., Ha, C.E. & Bhagavan, N.V. Structural insights into human serum albumin-mediated prostaglandin catalysis. *Protein science : a publication of the Protein Society* **11**, 538-545 (2002).
11. Klammt, S.*, et al.* Albumin-binding function is reduced in patients with decompensated cirrhosis and correlates inversely with severity of liver disease assessed by model for end-stage liver disease. *Eur J Gastroenterol Hepatol.* **19**, 257-263 (2007).
12. Jalan, R.*, et al.* Alterations in the functional capacity of albumin in patients with decompensated cirrhosis is associated with increased mortality. *Hepatology* **50**, 555-564 (2009).
13. China, L.*, et al.* Albumin Counteracts Immune-Suppressive Effects of Lipid Mediators in Patients With Advanced Liver Disease. *Clinical gastroenterology and hepatology : the official clinical practice journal of the American Gastroenterological Association* (2017).
14. China, L.*, et al.* ATTIRE: Albumin To prevenT Infection in chronic liveR failurE: study protocol for a single-arm feasibility trial. *BMJ Open* **6**, e010132 (2016).
15. China, L.*, et al.* Administration of Albumin Solution Increases Serum Levels of Albumin in Patients With Chronic Liver Failure in a Single-Arm Feasibility Trial. *Clinical gastroenterology and hepatology : the official clinical practice journal of the American Gastroenterological Association* (2017).
16. Margarson MP, S.N. Serum albumin: touchstone or totem? *Anaesthesia* **53**, 789-803 (1998).
17. Kahan, B.C., Forbes, A.B., Dore, C.J. & Morris, T.P. A re-randomisation design for clinical trials. *BMC Med Res Methodol* **15**, 96 (2015).
18. Bajaj, J.S.*, et al.* Second infections independently increase mortality in hospitalized patients with cirrhosis: the North American consortium for the study of end-stage liver disease (NACSELD) experience. *Hepatology* **56**, 2328-2335 (2012).
19. Bajaj, J.S.*, et al.* Survival in infection-related acute-on-chronic liver failure is defined by extrahepatic organ failures. *Hepatology* **60**, 250-256 (2014).
20. Bajaj, J.S.*, et al.* Linkage of gut microbiome with cognition in hepatic encephalopathy. *American journal of physiology. Gastrointestinal and liver physiology* **302**, G168-175 (2012).
21. Simon-Talero, M.*, et al.* Effects of intravenous albumin in patients with cirrhosis and episodic hepatic encephalopathy: a randomized double-blind study. *Journal of hepatology* **59**, 1184-1192 (2013).
22. regulations, C. Medicines for Human Use (Clinical Trials Regulations) 2004 Informed consent in clinical trials. (ed. Authority, H.R.) (http://www.hra.nhs.uk/, 2008).
23. Angeli, P.*, et al.* Diagnosis and management of acute kidney injury in patients with cirrhosis: Revised consensus recommendations of the International Club of Ascites. *Journal of hepatology* (2015).
24. European Association for the Study of the, L. EASL clinical practice guidelines on the management of ascites, spontaneous bacterial peritonitis, and hepatorenal syndrome in cirrhosis. *Journal of hepatology* **53**, 397-417 (2010).
25. Moore, K.P. & Aithal, G.P. Guidelines on the management of ascites in cirrhosis. *Gut* **55 Suppl 6**, vi1-12 (2006).
26. Runyon, B.A. & Aasld. Introduction to the revised American Association for the Study of Liver Diseases Practice Guideline management of adult patients with ascites due to cirrhosis 2012. *Hepatology* **57**, 1651-1653 (2013).

For peer review only - http://bmjopen.bmj.com/site/about/guidelines.xhtml

27. Bernardi, M., Caraceni, P., Navickis, R.J. & Wilkes, M.M. Albumin infusion in patients undergoing large-volume paracentesis: a meta-analysis of randomized trials. *Hepatology* **55**, 1172-1181 (2012).
28. Martin-Llahi, M., *et al.* Terlipressin and albumin vs albumin in patients with cirrhosis and hepatorenal syndrome: a randomized study. *Gastroenterology* **134**, 1352-1359 (2008).
29. Fernandez, J., *et al.* Effect of intravenous albumin on systemic and hepatic hemodynamics and vasoactive neurohormonal systems in patients with cirrhosis and spontaneous bacterial peritonitis. *Journal of hepatology* **41**, 384-390 (2004).
30. Sort P, N.M., Arroyo V, Aldeguer X, Planas R, Ruiz-del-Arbol L, Castells L, Vargas V, Soriano G, Guevara M, Ginès P, Rodés J. Effect of intravenous albumin on renal impairment and mortality in patients with cirrhosis and spontaneous bacterial peritonitis. *N Engl J Med.* **341**, 403-409 (1999).
31. Patel, A., Laffan, M.A., Waheed, U. & Brett, S.J. Randomised trials of human albumin for adults with sepsis: systematic review and meta-analysis with trial sequential analysis of all-cause mortality. *Bmj* **349**, g4561 (2014).
32. Caironi, P., *et al.* Albumin replacement in patients with severe sepsis or septic shock. *The New England journal of medicine* **370**, 1412-1421 (2014).
33. Reviewers, C.I.G.A. Human albumin administration in critically ill patients: systematic review of randomised controlled trials. *Bmj* **317**, 235-240 (1998).
34. Thevenot, T., *et al.* Effect of albumin in cirrhotic patients with infection other than spontaneous bacterial peritonitis. A randomized trial. *Journal of hepatology* **62**, 822-830 (2015).
35. Guevara, M., *et al.* Albumin for bacterial infections other than spontaneous bacterial peritonitis in cirrhosis. A randomized, controlled study. *Journal of hepatology* **57**, 759-765 (2012).

For peer review only

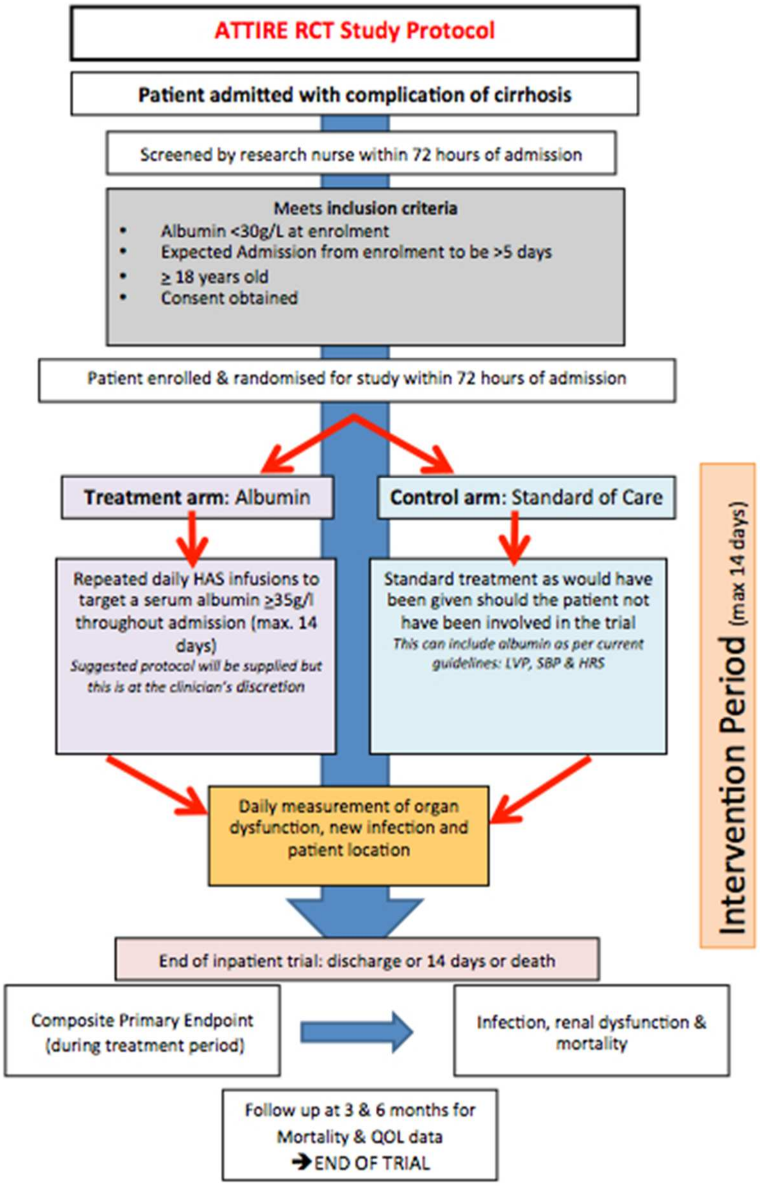

Figure 1. Overview of ATTIRE RCT Study Protocol

149x221mm (72 x 72 DPI)

Appendix 1 – Classification of infection

1. Spontaneous bacteraemia: positive blood cultures without a source of infection.
2. SBP: ascitic fluid polymorphonuclear cells >250 cells/mm^3
3. Lower respiratory tract infections: new pulmonary infiltrates in the presence of:
   i) at least one respiratory symptom (cough, sputum production, dyspnoea, pleuritic pain) with
   ii) at least one finding on auscultation (rales or crepitation) or one sign of infection (core body temperature >38°C or less than 36°C, shivering, or leukocyte count >10,000/mm3 or <4,000/mm3) in the absence of antibiotics.
4. Clostridium difficile Infection: diarrhoea with a positive C. difficile assay.
5. Bacterial entero-colitis: diarrhoea or dysentery with a positive stool culture for Salmonella, Shigella, Yersinia, Campylobacter,or pathogenic E. coli.
6. Soft-tissue/skin Infection: fever with cellulitis.
7. Urinary tract infection (UTI): urine white blood cell >15/high-power field with either positive urine gram stain or culture.
8. Intra-abdominal infections: diverticulitis, appendicitis, cholangitis, etc.
9. Other infections not covered above.
10. Fungal infections as a separate category.

For peer review only

Appendix 2 - Exploratory Objectives:

1. Effect of 20% HAS on the incidence, morbidity and mortality of the following infections:
    a. Bacterial
        i. Community acquired (diagnosed within 48 hours of admission in patients without previous hospitalisation in the last 6 months)
        ii. Nosocomial
        iii. Drug resistant pathogens
        iv. Culture negative sepsis (patients treated with antibiotics in the absence of microbiology evidence of infection)
        v. Clostridium Difficile infection
        vi. Source of infection (e.g. bacteraemia, respiratory, peritonitis, urine, skin etc.)
    b. Fungal
    c. Viral
2. Incidence, morbidity and mortality according to aetiology of Cirrhosis
3. Outcome for participants admitted to hospital for the first time with decompensated liver cirrhosis compared with participants who have previously been admitted to hospital with decompensated liver cirrhosis.
4. Requirement for ICU use of inotropes, vasopressors, hemofiltration and intubation and ventilation.
5. Analysis of samples from patients:
   a. Immune function studies using plasma samples – including but not limited to cell culture, qPCR, Albumin-ligand binding studies, western blot, flow cytometry and immunohistochemistry
   b. Plasma and urine lipidomic profile (in participants giving optional consent for urine samples)
6. Analysis of samples from patients providing additional optional consent (subject to further application for funding and ethical approval) :
   a. Stool microbiome analysis
   b. Genetic analysis of host response to infection
   c. Pathogen detection

For peer review only

To be printed on hospital headed paper

ATTIRE: Stage 2

Albumin To prevenT Infection in chronic liveR failurE

The Problem

The most common cause of death in patients with liver disease is infection
➔ this is because in liver disease the immune system does not work well, especially when you are unwell in hospital.

What we know

Albumin (a protein found in your blood) is lower in patients with liver disease. Research looking at blood samples has suggested that increasing the albumin levels in the blood can potentially improve the immune system in patients with chronic liver disease.

What question is this study trying to answer?

1. Does albumin actually stop patients admitted to hospital with chronic liver disease from getting infections? (or help them fight them)

What additional treatment or investigations may occur if I take part?

If you decide to take part, you will be assigned to either the 'albumin group' or the 'standard of care group'. This allocation will be random.

1. In the 'standard of care group', you will receive exactly the same treatment as you would have done had you not been in the trial.
2. If in the 'albumin group' you will be given an intravenous infusion of albumin every day whilst you are in hospital **and** you will also receive all the other treatment that you would have done had you not been in the trial. Albumin is a very safe protein infusion that is already commonly used in liver patients who have kidney problems or ascites (fluid in the abdomen).
3. We will collect some blood samples (3 times) during your admission and if you are happy we will also collect urine (2) and stool sample (1). Where possible the blood will be taken when your other blood tests collection occurs to avoid any additional tests.

Throughout the trial you will be asked to complete some questionnaires; this will start when you enter the study and at 3 and 6 months after you are discharged from hospital. If you are happy we will collect one blood sample at either of these appointments.

What are the possible advantages?

You may not have any direct benefit from taking part in the study but you will be a part of a large study that will help to decide whether this is a beneficial treatment for patients with liver disease to help decrease infection.

What are the possible disadvantages?

You may be connected to a drip for 1-2 hours a day (this can be when other drip medication is given). The side effects of albumin are very rare (listed in detail in the following pages).

For peer review only - http://bmjopen.bmj.com/site/about/guidelines.xhtml

To be printed on hospital headed paper

ATTIRE

Stage 2: Patient Information Sheet

Full study title:

Albumin **T**o preven**T** **I**nfection in chronic live**R** failur**E**.

A trial to investigate whether giving albumin to patients with advanced liver cirrhosis will reverse immune suppression and prevent infection.

Invitation to take part in research study

We would like to invite you to take part in a research trial. Before you decide we would like you to understand why the research is being done and what it involves. Please read this information sheet; one of the study team will answer any questions you may have. Talk to others about the study and ask us if there is anything that is not clear. Take time to decide whether or not you wish to take part.

The ATTIRE study is in two stages. The first stage included 80 patients and the second includes over 860 patients. They are very similar, but will look at slightly different outcomes.

This Patient Information Sheet is for Stage 2.

What is the purpose of the study?

Liver cirrhosis results in the liver tissue becoming scarred and developing lumps, these changes can prevent the liver from functioning properly. As the liver function worsens, symptoms such as fluid within the abdomen or legs, yellow skin, vomiting blood and confusion can develop. These are a result of advanced liver disease and are termed complications of cirrhosis. There are about 60,000 patient admissions to hospitals each year with such complications of liver cirrhosis. These patients are at higher risk of developing an infection during their hospital stay and this increase in risk is due to a weakened immune system. These infections can be very serious.

Giving fluid directly into the veins (intravenous) is known to be very important to help kidney function in patients with advanced liver disease. However, how much to give or indeed what is the best fluid to give is currently unknown. We have shown in laboratory testing, that albumin might be the best option as it can strengthen the immune system. However, it is unclear whether this will reduce the number of infections in hospital and that is what our trial wishes to investigate.

Albumin is found in the blood and is made in the liver. Patients with liver cirrhosis cannot make albumin as easily due to the damage to their liver. Albumin is already used to treat patients with liver cirrhosis and is known to be safe. In this stage of the study, patients will be given extra albumin to see if this helps their immune system to fight infections.

Why have I been chosen?

You are being asked to take part in this study as you have been admitted to hospital with complications of liver cirrhosis. Your doctor believes that you could be eligible to take part.

Do I have to take part?

It is up to you whether or not to take part in the study. If you decide not to take part, this will not affect the treatment you would normally receive. If you take part, you are free to withdraw at any time without giving a reason; this will not affect the care you receive.

For peer review only - http://bmjopen.bmj.com/site/about/guidelines.xhtml

What will happen to me if I take part?

If you take part in this study you will be either randomly allocated to the 'albumin group' or the 'standard of care group'.

If you are allocated to the 'albumin group', you will be given albumin solution as an intravenous fluid. Albumin has been shown to be safe in patients with liver disease. The amount of albumin that you are given will be decided by the amount that you already have in your blood. You will receive an infusion each day that you are in hospital, for a maximum of 14 days, until the levels in your blood have reached normal. You will also receive all of the other treatment that you would normally have had you not been in the study.

If you are in the 'standard of care group' you will have the treatment you would normally have, had you not been a part of the study, this may include albumin if you need it for another reason.

Regardless of which group you are in, you will have blood tests on most days while you are in hospital (as part of your normal care). At this time an additional sample (about 1 teaspoon) will be collected for up to 4 days during your time in the trial. You will also be asked to provide stool and urine samples while in the study, however this sample collection is optional and you can still take part in the study without providing these samples.

You also will be asked to complete some patient questionnaires during your time in the trial.

You will be contacted at 3 months and 6 months following your discharge from hospital to attend hospital visits, we will try to have these visits at the same time as your regular clinic appointments if possible.

What will I have to do?

If you agree to take part in the study you will be asked to sign a consent form. A member of the study team will then go through the initial assessments required for the study including collecting details of your medical history and any medication that you are currently taking. You will have tests (which will be part of your standard care) to look at your general health (e.g. heart rate and blood pressure), whether you have an infection, your liver function and the level of albumin already in your blood. Only if the level of albumin is below a certain level, will you be able to take part.

A research nurse will then come and record your blood tests and how well you are every day whilst you are in hospital. When you are discharged from hospital the research nurse will arrange to see you 3 months and 6 months after discharge to ask you some questions about how well you are feeling, this will be at the same time as your other clinic appointments if possible.

If you are a woman of child bearing potential, a pregnancy test will be carried out.

Starting Treatment

Once all of your tests have been completed and if you are still eligible to participate, a computer outside of the hospital will decide whether you will be in the 'albumin group' or the 'standard of care group', this is called randomisation. You will get exactly the same hospital care as you would have previously but with (or without) additional albumin infusions. This is so the trial investigators can see if there is an advantage, or disadvantage, of being given albumin.

The test for the amount of albumin in your blood will be used to work out how much albumin will be given, if you are in the 'albumin group', this will be given by a nurse or doctor. We expect the test to be performed anyway and so will still be done if you are in the 'standard of care group' as well. The albumin will be given directly into your blood through an intravenous cannula (a tube inserted into your vein to give fluid); the cannula will already be present as part of your standard care, an extra one will *not* be required. This will happen each day that you are on the study (a maximum of 14 days).

You will have blood tests on most days for liver function and to look for infections. These would normally be taken during your hospital stay. An extra blood sample (1 teaspoon) will be taken at the same time on

For peer review only - http://bmjopen.bmj.com/site/about/guidelines.xhtml

three days throughout your admission while in the study and will be used to look at your immune system. You will also be asked to provide urine and stool samples whilst in the study, however these will be optional samples. The stool samples and any samples left over will be stored for future research (for which further ethical committee review will be required).

If you do get an infection whilst in the study, you will be given the normal treatment for that type of infection that may include albumin. Taking part in the study will not affect the care that you normally get from your doctor.

End of study treatment

When you have taken part in the study for 14 days or your when your doctor feels you are ready to be discharged (if this is before 14 days of being on the study), you will stop your study treatments. This means that you will no longer be given albumin as part of the study or have any samples collected that are not part of your standard treatment. You will be asked to complete a questionnaire when you are discharged. If you doctor feels that you still need albumin, you will continue to receive it.

The study team will then also contact you at 3 months and 6 months after you have been discharged from hospital, this will be at the same time as your other clinic appointments if possible. At these visits you will undergo tests that you would normally have as part of your standard of care and you will be asked to complete some questionnaires to see how you are feeling and what treatment you have had since being discharged from hospital. If you agree an extra blood sample (1 teaspoon, as above) will be taken at the same time as one of these appointments.

At the end of the study, if you agree, we will access information about you that is held by NHS digital, the Office of National Statistics (ONS) and other central NHS bodies regarding hospital admissions and mortality statistics for the six months following treatment. This is data that is collected as part of standard practice and will involve no additional involvement by you. The data, which is highly regulated, will be used to see if there are any long term effects of the treatment for you and the services that you may access.

When should I contact the study team?

You should contact a member of the study team or a doctor or nurse on your ward if you feel unwell at any time or have any incidents that affect your physical wellbeing. Contact details of the study team at your hospital and the Chief Investigator are available at the end of this leaflet.

What happens if I become pregnant?

All women of child-bearing potential will have a pregnancy test before entering the study. If you do become pregnant during treatment, you should inform your study team. It is not thought that albumin will affect pregnancy.

What are the alternative treatments if I don't take part?

If you do not take part in the study, standard treatment will be offered, which may include the use of albumin. Albumin is currently used for several reasons in patients with liver disease for example those who require extra fluid to support their kidneys, or to replace fluid that has been drained from the abdomen, or in those who develop infections.

What are the possible disadvantages and risks of taking part?

There are some possible risks and disadvantages that you should consider before taking part in this study:

- Wherever possible we will ensure that the blood tests needed for the study are taken at the same time as those needed for your routine care, but you may be asked to have additional samples taken if this is not possible. The risks of having a blood test include local bruising and discomfort.
- You may experience discomfort and bruising from fluid infusion.
- As the albumin is taken from human blood, in theory there is chance that you could get an infection (virus) from the person who has donated the blood. However there are no reports of patients getting

For peer review only - http://bmjopen.bmj.com/site/about/guidelines.xhtml

viral infections from albumin that is made following European manufacturing standards. The albumin given as part of the study is the same as that which would be given to you as part of your regular care by your doctor which you may have received as a treatment in the past.

- A cannula (very small plastic tube) will be used to give you the albumin. We would expect that you already will have one of these to give your other medications whilst in hospital and will use the same cannula. However if you don't have any medications or fluid that need to be given into the vein we will need to place a small cannula into the vein in order to give you the albumin.
- If in the albumin group you will be connected to an albumin infusion for around 1-2 hours a day. You can still move around the ward with this but will need to have a drip stand with you.

What are the possible benefits of taking part?
There may be no benefit to you from taking part, however, intravenous albumin may help your kidneys and raise blood pressure that can be low in patients with liver problems. Taking part may also provide additional information about patients with liver disease.

What are the side effects of any treatment received when taking part?
The side effects of albumin are rare but can include the following:

	Very Common	Common	Uncommon	Rare	Very Rare
Immune system disorders					Anaphylactic shock
Gastrointestinal disorders				Nausea	
Skin and subcutaneous tissue disorders				Flushing, skin rash	
General disorders and administration site conditions				Fever	

*__Very Common:__ in more than 1 in 10 patients treated; **Common:** in less than 1 in 10, but more than 1 in 100 patients treated; **Uncommon:** in less than 1 in 100, but more than 1 in 1000 patients; **Rare:** in less than 1 in 1000, but more than 1 in 10000 patients treated; **Very rare:** in less than 1 in 10000 patients treated, including isolated cases

Other side effects observed after placing Human Albumin on the market are: Hypersensitivity/Allergic reactions, Headache, Rapid heart beat, Abnormally low blood pressure, Breathlessness or breathing discomfort, Vomiting, Altered sense of taste, Hives, Itchiness, Chills, Heart attack, Irregular heart beat, Accumulation of fluid in the lung.

If any of the above reactions occur these will be treated as they would in normal standard care.

What if relevant new information becomes available?
Sometimes we get new information about the treatments being studied. If this happens, your study team will tell you about it and discuss how this may impact you. They will discuss whether you want to or should continue in the study.

What happens when the research study stops?
If you still require any treatment once the study stops, your doctor will prescribe the treatment most suitable for you.

Expenses and payments
You will not receive any payments for participating in this study.

For peer review only - http://bmjopen.bmj.com/site/about/guidelines.xhtml

What if something goes wrong?

If you have any concerns about the study, you should discuss them with your local study team first. You can also contact the Chief Investigator for the study, Dr Alastair O'Brien, on 020 7679 6851 who will be happy to discuss any of your concerns.

If you would like to discuss your concerns with someone not involved in the study, please contact your local Patient Advice and Liaison Service (PALS). Information on how to contact your local PALS can be found at the end of this information sheet.

Every care will be taken during this clinical study. However, in the unlikely event that you are injured by taking part, compensation may be available. If you suspect that the injury is the result of the Sponsor's (UCL) or the hospital's negligence then you may be able to claim compensation. After discussing it with your study team, please write to Dr Alastair O'Brien who is the Chief Investigator for the clinical study, about details of your claim. The Chief Investigator will then pass the claim to the Sponsor's Insurers, via the Sponsor's office. You may have to bear the costs of the legal action initially, and you should consult a lawyer about this. Contact details of the study team at your hospital and the Chief Investigator are available at the end of this leaflet.

You may also be able to claim compensation for injury caused by participation in this clinical study without the need to prove negligence on the part of University College London or another party. You should discuss this possibility with your study team in the same way as above.

Regardless of this, if you wish to complain, or have any concerns about any aspect of the way you have been approached or treated by members of staff or about any side effects (adverse events) you may have experienced due to your participation in the clinical study, the normal National Health Service complaints mechanisms are available to you. Please ask your study team if you would like more information on this. Details can also be obtained from the Department of Health website: http://www.dh.gov.uk.

Will my taking part in this study be kept confidential?

Yes, it will. We will request your approval to contact and inform your GP in the consent form about your participation in the study. We will send information relating to this research to the Comprehensive Clinical Trials Unit at University College London (UCL), where the study is being managed. Relevant information about you and all other patients in the study will be stored on password protected computers and in locked filing cabinets and will only be available to the staff working on the study. The information stored by the Comprehensive Clinical Trials Unit will contain a unique code, your initials, and month and year of birth. Your full name, address, contact details relevant medical history and relevant test results will be maintained at your local hospital. The information may be accessed by authorised personnel from UCL and the NHS hospital where you are being treated for auditing and monitoring purposes or by the regulatory authorities for inspection purposes to make sure the study is being carried out properly. Data collected during the study may be sent pseudo-anonymously (partially linked) to associated researchers in countries where the laws don't protect your privacy to the same extent as the law in the European Economic Area (EEA) but we will take all necessary steps to protect your privacy. Any data sent will only contain your unique code, your initials and month and year of birth. A data protection officer at UCL will be informed about any potential data transfer. You will be asked whether you agree to this when you sign the consent form.

If you withdraw consent from further study treatment, unless you object, your data and samples will remain on file and will be included in the final study analysis. In line with Good Clinical Practice guidelines, at the end of the study, your data will be securely archived for a minimum of 5 years. Arrangements for confidential destruction will then be made.

What will happen to the results of the research study?

If you choose to take part in the study, you will be given a unique trial identification code. All information collected from you for the study will be associated with this unique code, your initials, and month and year

For peer review only - http://bmjopen.bmj.com/site/about/guidelines.xhtml

of birth. No other personal information will be sent to personnel outside your hospital and no one outside your hospital care team will be able to identify you from this information.

The blood, urine and stool samples collected for this study will be sent to the University College London, where they will be stored securely with your study specific identification number, initials, and month and year of birth. The blood and urine samples will be analysed at UCL to look at your immune system. The stool samples will be stored securely at UCL to be used for future research. Any future research carried out on the samples will be subject to further ethical committee review. It may be possible that the samples, or part of the samples, will be sent to countries outside the EEA where the laws for data protection differ from those in the UK. The urine and stool samples are optional samples and will not affect your participation in the study.

The Comprehensive Clinical Trials Unit will analyse the data collected on the patients in this stage of the study after all 866 patients have completed their treatment. The results of the study will be presented at a scientific conference and will be published in a scientific journal which will be accessible to the public. None of the research participants will be identified in any report or publication. Should you wish to see the results, or the publication, please ask your study team.

Who is conducting and funding the research?
This study is being managed by the Comprehensive Clinical Trials Unit at the University College London. Funding for the research has come from the Department of Health and Wellcome Trust.

Who has reviewed the study?
All research in the NHS is reviewed by a Research Ethics Committee and an agency of the Department of Health called the MHRA (the Medicines and Healthcare products Regulatory Agency). Both of these groups have confirmed that they are content for the study to go ahead and are regularly updated on its progress by the Comprehensive Clinical Trials Unit.

Who to contact for further information?
You are encouraged to ask any questions you wish, before, during or after your treatment. If you have any questions about the study, please speak to a member of the study team. If you wish to read the research on which this study is based, please ask your study team. If you require any further information or have any concerns while taking part in the study please contact one of the following people at your study site:

Doctor

Name *add name of PI* Tel. Number: *add Tel. number*

Study Nurse

Name *add name* Tel. Number: *add Tel. number*

PALS Add local PALS contact details

Or contact the Chief Investigator for the study:
Dr Alastair O'Brien at University College London, Tel. Number: 020 7 679 6851

Before you sign the informed consent form, you should ask questions about anything that you do not understand. The study staff will answer any questions before, during and after the study.

For peer review only - http://bmjopen.bmj.com/site/about/guidelines.xhtml

Thank you for taking the time to read this information sheet.

For peer review only

For peer review only - http://bmjopen.bmj.com/site/about/guidelines.xhtml

To be printed on hospital headed paper

ATTIRE

Stage 2: Patient Informed Consent Form

Albumin **T**o preven**T** **I**nfection in chronic live**R** failur**E** (ATTIRE)

PATIENT ID: ☐☐☐-☐☐☐-☐☐ SITE: ☐☐☐

MONTH AND YEAR OF BIRTH: ☐☐☐-☐☐☐☐ PATIENT INITIALS: ☐☐☐

M M M - Y Y Y Y

This Informed Consent Form is intended for consenting patients into Stage 2 of the ATTIRE study.

Please insert your **initials** in the boxes to confirm consent:

	**Initials**
1. I confirm that I have read and understood the Patient Information Sheet version 4.0 dated 14Jun2016 for the ATTIRE study and have had the opportunity to consider the information and ask questions, which have been answered to my satisfaction.	
2. I understand that my participation is voluntary and that I am free to withdraw at any time, without giving a reason and without my medical care or legal rights being affected.	
3. I understand that sections of my medical records may be looked at by properly authorised personnel involved in the running of the study or from regulatory authorities where it is relevant to my taking part in this study. I give permission for these individuals to have access to my records.	
4. I agree to provide blood samples.	
5. I agree to take part in the ATTIRE study.	
6. I agree to be contacted by the research team 3 and 6 months after my hospital discharge.	

For peer review only - http://bmjopen.bmj.com/site/about/guidelines.xhtml

Optional Consent:

The following are optional. Declining to any of the below will not prevent you from taking part in the trial. Please initial in the boxes to confirm consent:	Initials
7. I give permission for my study data to be sent to an associated researcher outside of the EEA.	
8. I agree to provide urine samples.	
9. I agree to provide a stool sample.	
10. I agree to my remaining samples, not used in this study, being used in future, ethically approved, research.	
11. I understand that the information held and maintained by NHS Digital, the Office of National Statistics and other central UK NHS bodies may be used to provide information about my health.	
12. I give permission for my GP to be informed about my participation.	

For peer review only

Patient/ Legal Representative's Name (score through as applicable)	Signature	Date
Person Taking Consent	Signature	Date
Witness (if applicable)	Signature	Date

For peer review only - http://bmjopen.bmj.com/site/about/guidelines.xhtml

Appendix 4

	***Screening/* Day 1 (Randomisation)** *(within 72hrs of admission)*	***Treatment Period*** *(Day 2- Day 14)*	***End of Treatment***	***Discharge***	***Follow up*** *(3 & 6 months +/- 1 month)[d]*
Eligibility screen	X				
Informed consent	X				
Medical history	X				
Blood tests and sample collection*	X	X	X		X
Clinical observations recorded	X	X	X		
Randomisation	X				
Dosing with HAS/Standard of care	X[b]	X			
Infection/new (change) in antibiotics	X	X	X		
Patient location recorded	X	X	X	X	X
Concomitant Medication	X	X	X	X	X
Pregnancy test (βhCG) (women of child bearing potential only)	X				
Survival		X	X		X
CSRI Questionnaire	X[c]				X
EQ-5D-5L Questionnaire	X			X	X

*Blood test include: FBC, U&ES, LFTs, CRP, INR, Serum Albumin concentrations. From patients who provide additional consent blood will be collected at randomisation (day 1) and on days 5 and 10 +/- 2 days. Urine sample will be collected on day 1 and day 5 +/- 2 days and A stool sample will be collected once during the patients treatment period. A blood sample from patients who consent will be taken at either the 3 or 6 month follow up visit.

[b] If randomised to receive the HAS treatment the volume of HAS given will be determined by the Serum Albumin results (see section 6.4.1.2)

[c] The CRSI should be completed before randomisation. If this is not possible, the questionnaire should be completed as soon as the patient is able, prior to discharge. The questionnaire should be completed in relation to the three months prior to admission – details of the current hospital admission should not be included.

[d] If the patient has been re-randomised into the study, before completing the follow up visits for the previous randomisation; the patient should not undergo the follow up activities relating to that randomisation The follow up visits should only be conducted for the current randomisation.

BMJ Open

ATTIRE: Albumin To prevenT Infection in chronic liveR failurE: Study Protocol for an Interventional Randomised Controlled Trial

Journal:	*BMJ Open*
Manuscript ID	bmjopen-2018-023754.R1
Article Type:	Protocol
Date Submitted by the Author:	15-Aug-2018
Complete List of Authors:	China, Louise; UCL, Division of Medicine<br>Skene, Simon; University of Surrey, Surrey Clinical Trials Unit<br>Bennett, Kate; UCL, Comprehensive Clinical Trials Unit<br>Shabir, Zainib; UCL, Comprehensive Clinical Trials Unit<br>Hamilton, Roseanna; UCL, Comprehensive Clinical Trials Unit<br>Bevan, Scott; UCL, Comprehensive Clinical Trials Unit<br>Chandler, Torsten; UCL, Comprehensive Clinical Trials Unit<br>Maini, Alex; UCL, Division of Medicine<br>Becares, Natalia; UCL, Division of Medicine<br>Gilroy, Derek; UCL, Division of Medicine<br>Forrest, Ewan; NHS Greater Glasgow and Clyde, Glasgow Royal Infirmary<br>O'Brien, Alastair; UCL, Division of Medicine
**Primary Subject Heading**:	Gastroenterology and hepatology
Secondary Subject Heading:	Evidence based practice, Health economics, Research methods
Keywords:	Adult gastroenterology < GASTROENTEROLOGY, Hepatobiliary disease < GASTROENTEROLOGY, Gastroenterology < INTERNAL MEDICINE

SCHOLARONE™ Manuscripts

ATTIRE: Albumin To prevenT Infection in chronic liveR failurE: Study Protocol for an Interventional Randomised Controlled Trial

Louise China*[1], Simon S Skene[2], Kate Bennett[3], Zainib Shabir[3], Roseanna Hamilton[3], Scott Bevan[3], Torsten Chandler[3], Alexander A Maini[1], Natalia Becares[1], Derek Gilroy[1], Ewan Forrest[4], Alastair O'Brien[1]

[1] Division of Medicine, University College London, University Street, London
[2]School of Biosciences and Medicine, University of Surrey
[3] Comprehensive Clinical Trials Unit, UCL, Gower Street, London, WC1E 6BT
[4]Department of Gastroenterology, Glasgow Royal Infirmary, Castle Street, Glasgow G4 0SF

[*]Correspondence: louise.china@ucl.ac.uk
s.skene@surrey.ac.uk
k.bennett@ucl.ac.uk
z.shabir@ucl.ac.uk
roseanna.hamilton@ucl.ac.uk
a.maini@ucl.ac.uk
natalia.becares.13@ucl.ac.uk
d.gilroy@ucl.ac.uk
Ewan.Forrest@ggc.scot.nhs.uk
a.o'brien@ucl.ac.uk

For peer review only

Abstract

Introduction
Circulating Prostaglandin E_2 levels are elevated in acutely decompensated cirrhosis and have been shown to contribute to immune suppression. Albumin binds to and inactivates this immune-suppressive lipid mediator. Human Albumin Solution (HAS) could thus be repurposed as an immune restorative drug in these patients.

This is a phase III randomised controlled trial to verify whether targeting a serum albumin level of $\geq$35g/l in hospitalised patients with decompensated cirrhosis using repeated intravenous infusions of 20% HAS will reduce incidence of infection, renal dysfunction and mortality for the treatment period (maximum 14 days or discharge if <14 days) compared to standard medical care.

Methods and Analysis
ATTIRE stage 2 is a multicentre, open label, interventional randomised controlled trial. Patients with decompensated cirrhosis admitted to hospital with a serum albumin of <30g/L are eligible, subject to exclusion criteria. Patients randomised to intravenous HAS will have this administered, according to serum albumin levels, for up to 14 days or discharge. The infusion protocol aims to increase serum albumin to near normal levels.

The composite primary endpoint is: new infection, renal dysfunction or mortality within the trial treatment period. Secondary endpoints include mortality at up to 6months, incidence of other organ failures, cost effectiveness and QOL outcomes and time to liver transplant. The trial will recruit 866 patients at more than 30 sites across the United Kingdom.

Ethics & Dissemination
Research ethics approval was given by the London-Brent research ethics committee (ref: 15/LO/0104). The clinical trials authorisation was issued by the medicines and healthcare products regulatory agency (ref: 20363/0350/001-0001). The trial is registered with the European Medicines Agency (EudraCT 2014-002300-24) and has been adopted by the NIHR (ISRCTN 14174793). This manuscript refers to version 6.0 of the protocol. Results will be disseminated through peer reviewed journals and international conferences. Recruitment of the first participant occurred on 25 Jan 2016.

For peer review only

Strengths and limitations of this study

- This is the first prospective, interventional RCT with the primary aim of assessing the effects of **targeted** 20% Human Albumin Solution (HAS) infusions on infection, organ failure and death in hospitalised patients with decompensation of liver cirrhosis.
- Study design was optimised by a prior 80 patient feasibility study
- Cost effectiveness and quality of life assessment will review whether any outcomes are sustainable and feasible in a national health service setting
- The study is un-blinded to site investigators due to lack of agreement regarding the control infusion fluid and high cost required
- Patients in the control arm may receive 20% HAS as part of current international guidance as it would be unethical to withhold this in certain situations.

Introduction

Liver disease is an increasing cause of mortality currently in the UK and is the fifth most common cause of death[1]. These deaths are predicted to double over the next 20 years[2]. Mortality is mainly seen in the middle-aged group. Liver disease has surpassed lung and breast cancer as the leading cause of years of working life lost, and is set to overtake ischaemic heart disease within 2–3 years[3].

Patients with complications of liver failure secondary to liver cirrhosis are described as decompensated patients. They are highly prone to bacterial infection[4] secondary to immune dysfunction[5], with nosocomial (hospital-acquired) infection rates of 35% compared to five per cent in non-cirrhotic patients[6,7]. Of those who develop infection with organ dysfunction, 60-95% will die, often following prolonged intensive care unit admission[8]. There is, however, no medical strategy to restore immune competence.

It has been demonstrated that elevated circulating Prostaglandin E_2 (PGE_2) levels contribute to immune suppression in acutely decompensated patients[9]. The plasma protein albumin binds and catalyses the inactivation of PGE_2[10]. The binding capacity of endogenous albumin is known to be defective in cirrhosis[11,12] and improves following albumin infusions[13]. Albumin is synthesised in the liver and levels fall as the synthetic function of the liver declines in advanced cirrhosis. Therefore low albumin levels, with defective binding function, make PGE_2 more bioavailable. We found a serum albumin of < 30g/L predicted plasma induced macrophage dysfunction in a small cohort of decompensated cirrhosis patients[9] and this was reversed when albumin levels were increased to ≥30g/L. This finding has recently been validated in a multicentre clinical trial[13], however it is not known if these ex-vivo observations translate into an improvement in clinical outcomes.

We propose a novel strategy to repurpose 20% Human Albumin Solution (HAS) as an immune restorative drug in patients with decompensated liver cirrhosis with the aim of maintaining serum albumin at near normal levels.

The ATTIRE phase II single arm, multicentre feasibility trial[14] (n=80) verified that daily intravenous human albumin infusions restored serum albumin levels to near normal in

hospitalised decompensated cirrhosis patients, that it was safe and that there was physician equipoise prior to proceeding to a large RCT[15]. It has also allowed refinement of our definitions of RCT outcomes.

This phase III randomised controlled trial (RCT) (n=866) will assess the impact of 20% HAS treatment on the incidence of nosocomial infections, organ dysfunction and mortality in patients admitted to hospital with decompensation of liver cirrhosis.

Methods and Analysis

Primary Objective:

To verify whether raising and maintaining serum albumin levels ≥30g/L in patients admitted with decompensated cirrhosis using repeated 20% HAS infusions will reduce incidence of infection, renal dysfunction and mortality for the treatment period (maximum 14 days or discharge if <14 days) compared to standard medical care (figure 1). To raise and maintain serum albumin levels above 30g/L, 20% HAS will be prescribed and administered in the intervention arm if levels are below 35g/L.

Secondary Objectives are to assess the impact of the intervention on development of other organ dysfunction (cardiac, respiratory, brain and liver), cost effectiveness; quality of life and sustainability of any outcomes over a 6 month follow up period.

Trial Design

This is a multicentre, open label, randomized controlled trial in which patients will either be treated with 20% HAS to raise and maintain serum albumin above 30g/L or will continue to receive their usual standard of care treatment. 866 sequential patients admitted to 30 UK participating hospitals with a clinical diagnosis of cirrhosis and decompensation will be screened using the inclusion and exclusion criteria (Table 1). Decompensation includes: jaundice, ascites, hepatic encephalopathy, variceal bleeding, coagulopathy and hepatorenal syndrome.

Patient Inclusion Criteria	Patient Exclusion Criteria
All patients admitted to hospital with acute onset or worsening of complications of cirrhosis	Advanced hepatocellular carcinoma with life expectancy of less than 8 weeks
Over 18 years of age	Patients who will receive palliative treatment only during their hospital admission
Predicted hospital admission ≥ 5 days at trial enrolment, which must be within 72 hours of admission	Patients who are pregnant
Serum albumin <30g/l at screening	Known or suspected severe cardiac dysfunction
Documented informed consent to participate (or consent given by a legal representative)	Any clinical condition which the investigator considers would make the patient unsuitable for the trial
	The patient has been involved in a clinical trial of Investigational Medicinal Products (IMPs) within the previous 30 days that would impact on their participation in this study
	Trial investigators unable to identify the patient (by NHS number)

Table 1. Patient Selection Criteria

For peer review only

As advanced liver disease requires frequent hospital re-admissions, patients may be enrolled in the RCT more than once, with a 30 day "washout period" following discharge after the first enrolment, during which time they cannot be enrolled for a second time. This is to account for albumin's half-life of 18-21 days[16]. Patients re-entering the trial in this way will be re-randomised, so that each enrolment will be considered as an independent patient 'presentation'[17].

To ensure homogeneity in the approach to patient recruitment, intervention and data collection all sites received introduction training and regular follow up re-training plus monitoring and support visits from the sponsor.

Clinical trial endpoints

Primary endpoint

A composite endpoint comprising incidence of infection, renal dysfunction and mortality within the treatment period (for a maximum of 14 days OR when the patient is considered fit for discharge if < 14 days).

The three components of the composite endpoint are:

1. New Infection: indicated by clinician diagnosis and clinical evidence provided on completed infection case report form (CRF). Blinded data from a clinically representative sample of patients will be scrutinised for the presence of infection by a Clinical Trial Endpoint Review Committee. The committee will contain microbiologists who will validate the presence of infection according to peer reviewed criteria[18] (Appendix 1).
2. Renal Dysfunction: a serum creatinine increase of ≥50% as compared to serum creatinine at randomisation OR the patient initiated on renal replacement support (either haemodialysis or haemofiltration) OR a rise in serum creatinine of ≥26.5 μmol/L within 48hours.
3. Mortality

Primary endpoint data will be recorded throughout the treatment period; however only contributing events captured on the treatment day 3 case report form (CRF) through to the day 15 CRF (the end of treatment examination day) will contribute to the primary outcome. This allows the biological effect of albumin to be established at least 24 hours following initiation of treatment.

If the participant is discharged or deemed medically fit for discharge prior to day 15, no further primary outcome data will be measured after this date, as this will signal the end of the participant's treatment period.

Secondary Endpoints:

1. Mortality at 28 days post randomisation and at 3 & 6 months post discharge
2. Time to outcome (first event of infection/renal dysfunction/death)
3. Incidence of respiratory, circulatory and cerebral dysfunction during the treatment period (based on modified components of the CLIF-SOFA[19] score).
4. Incidence of liver transplant within six months of treatment
5. Total amount of HAS administered during the treatment period
6. Duration of hospital stay
7. Change in prognostic score (assessed by MELD) between randomisation and end of treatment period.

For peer review only

8. Days in ICU during treatment period
9. Incremental cost and cost-effectiveness up to 6 months post discharge
10. Impact on quality of life (QOL) up to 6 months post discharge
11. Safety and tolerability of HAS as indicated by Serious Adverse Events (SAEs)
12. Use of terlipressin for (a) renal dysfunction, (b) hypotension, (c) variceal bleeding.

Patients in the RCT will be followed up 3 and 6 months (+/- 1 month) following discharge from hospital. Exploratory objectives are listed in Appendix 2.

Patient population

This will include all patients admitted to hospital with the complications of decompensated liver cirrhosis and serum albumin < 30 g/L, aged over 18 years with anticipated hospital length of stay of 5 or more days at trial enrolment, which should be no later than 72 hours from admission. This is subject to exclusion criteria as detailed in table 1. The diagnosis of cirrhosis will be made by the clinical team as per standard UK practice and does not require liver biopsy or imaging. Acute decompensation of liver cirrhosis associated with organ failures is termed ACLF (Acute on Chronic Liver Failure). ACLF has a number of definitions[20,21] based on the SOFA (sequential organ failure assessment) score, all are associated with a poor prognosis. This study will include patients with decompensated cirrhosis with and without ACLF, it will also record the development of ACLF during the study treatment period.

Consent

Patient information sheets (Appendix 3) will be given to and discussed with potential patients before consent is sought. Informed consent will be obtained from each participant or his or her legal representative. Patients who lack mental capacity, for any reason, are not excluded from the trial. An important subgroup of patients will have hepatic encephalopathy and these patients may lack capacity to consent, however these patients may be amongst those that receive maximum benefit from the intervention[21-23]. In this case consent will be sought from an appropriate legal representative independent of the research team as per current UK clinical trials regulations[24].

Intervention

After randomisation (when serum albumin is <30g/L) patients will receive either daily dose of 20% HAS intravenously if their serum albumin level is less than 35g/L (at approximately 100mls/hour) or standard medical care (which may include 20% HAS infusions for indications listed in established guidelines only – see below) for a maximum of 14 days or discharge (if < 14 days). The volume of HAS each day will be determined by the patient's serum albumin level on that day (or the closest previous measurement if there are no results from that day available).

Table 2 shows the suggested dosing protocol for albumin administration in the treatment arm group. This is a suggested regimen and responsible clinicians are given flexibility to alter this depending on the clinical situation. The effectiveness of this protocol, and approach, was verified in the ATTIRE feasibility study[15]. Differing regimens may be used to cover large volume paracentesis (8g of albumin per litre of ascites drained) or treat Hepatorenal syndrome (1g of albumin per kilogram of body weight) as per international guidelines[25,26] but HAS *must* be prescribed and given if serum albumin <35g/L unless there are any safety concerns**.** All variations will be recorded in the patient's daily Case Report Form (CRF).

For peer review only - http://bmjopen.bmj.com/site/about/guidelines.xhtml

Patient's Serum Albumin Level	Amount of 20% HAS to be administered
≥35 g/L	none
30-34 g/L	100mls
26-29 g/L	200mls
20-25 g/L	300mls
<20 g/L	400mls

Table 2. Treatment arm dosing protocol for 20% HAS administration (amounts per day) as advised by measured serum albumin level on that day.

20% HAS will only be given in the Standard of Care arm if the patient requires large volume paracentesis or has SBP or HRS (as per established guidelines[26-28]). This needs to be clearly recorded in the patient's CRF and if HAS is given for any other indication in the Standard of Care arm this will be considered a protocol deviation. The administration of HAS in the Standard of Care arm will be closely monitored by the Independent Data Monitoring Committee (IDMC).

Randomisation will use a minimisation algorithm incorporating a random element, stratifying by centre, MELD score, and number of organ dysfunctions, serum albumin level and if antibiotics are currently being prescribed. To ensure maximum balance is achieved across the stratification factors, minimisation will be carried out on these factors separately.

Evaluations during and after treatment

Clinical, biochemical, microbiological, health economic and quality of life data will be collected during the trial treatment period (see Appendix 4) using information from hospital notes that is recorded as standard of care. There is follow up at 3 and 6 months. The blood, urine and stool samples collected for immune function tests will be analysed in a blinded fashion at a central site.

Patient and Public Involvement

Patients who have had a previous diagnosis of liver cirrhosis were involved in the development of the research question and outcome measures. Patient feedback led to the addition of quality of life evaluation. The study protocol and patient information leaflets were reviewed by our patient representatives with positive feedback. In addition an independent patient representative sat on the ethics approval panel. Results will be disseminated to patients via open access publication and our local trials teams.

Statistical considerations

Sample Size

A 30% incidence of nosocomial infection in decompensated cirrhosis patients is well documented with up to 30% of these patients developing organ dysfunction[35] and an overall mortality of 38% at 1 month[36]. These figures are supportive of 30% as a conservative estimate for the composite primary endpoint of incidence of new infection, renal dysfunction or mortality up to 14 days from randomisation.

We have assumed that the "immune-restorative" albumin treatment would reduce this rate by 30% to a rate of 21%, which would be considered clinically relevant. 389 patients per arm would be sufficient to detect such a difference with 80% power at a significance level of 0.05. Allowing for loss-to-follow-up/withdrawal of 10% from the trial, we will aim to recruit 433 to each arm (866 in total).

For peer review only

Statistical Evaluation
A detailed statistical analysis plan (SAP) will be written and approved by the Trial Steering Committee before the substantive analysis of unblinded trial data.

Baseline characteristics will be summarised by treatment using appropriate descriptive statistics; means and standard deviations for approximately normally distributed variables, medians and interquartile ranges for non-normally distributed variables and counts (percentages) for categorical variables.

Primary outcome
The primary outcome is the difference in event rates, according to treatment, of the composite endpoint of infection, renal dysfunction and mortality within the intervention period (from ≥ 24 hours from the start of treatment/randomisation up to a maximum of fourteen days or up to discharge if this is prior to fourteen days).

Since the primary outcome has a binary classification, logistic regression will be used to determine whether there is any difference in rates due to treatment, by inclusion of a binary covariate indicating treatment. The results will be adjusted for pre-determined prognostic factors used as stratifying variables in the randomisation, which shall be included as additional covariates in the model. The model coefficient due to treatment will give an estimate of the difference in log odds, or equivalently (exponentiated to give) an estimate of the odds multiplier, i.e. the change in odds of a negative outcome on the composite endpoint due to treatment with albumin. It is expected that this effect will be negative, so that treatment with albumin is seen to reduce the odds of a negative outcome. A reduction from 30% to 21% would be associated with a reduction in odds of around 38%. Predicted probabilities will be presented for the composite outcome for each of the treatment arms, adjusted for the model covariates.

The effect of treatment on the individual components, infection, renal dysfunction and mortality will be reported separately.

Secondary and exploratory outcomes
Secondary and exploratory outcomes will be handled similarly. Continuous secondary outcome measures will be analysed using a linear regression model, and time-to-event outcomes using Kaplan Meier plots and Cox's proportional hazards models where covariate adjustment is necessary.

All statistical tests will use a 2-sided p-value of 0.05, unless otherwise specified, and all confidence intervals presented will be 95% and two-sided. All statistical analysis will be performed using Stata (StataCorp, College Station, TX, USA).

Economic Evaluation
The economic evaluation will consist of two parts. The primary analysis will be a decision analytic model comparing the expected lifetime costs and benefits of treatment reported in terms of the cost per incremental quality adjusted life years (QALYs) gained. QALYs will be calculated based on the health related quality of life (HRQL) and mortality data collected during the trial. HRQL will be measured according to the EQ-5D-5L (www.euroqol.org), which we will collect at baseline, discharge, 3 months and 6 months (+/- 1 month) for each individual patient. If patients are re-randomised they will have separate follow up events for each episode of re-randomisation, unless the patient is re-randomised within the follow up phase of the study. Utility scores will be calculated using UK-specific tariffs and adjusting for

For peer review only

baseline differences in patients in the trial arms if necessary. These will be extrapolated over the expected lifetime of the patient and where necessary will draw on the existing literature. The final form of the decision analytic model will be determined during the trial period. Appropriate methods for estimating changes in health over time, such as state-transition models, will be given consideration.

The secondary economic analysis will be a 'within-trial' analysis, reporting on the incremental cost and benefits observed during the trial period and follow-up periods. The cost-effectiveness measures in the within-trial analysis will be the incremental cost per infection avoided and the incremental cost per QALY gained. Costs will be measured as described below. Infections avoided will be derived from the primary outcome in the trial. Patient resource use will be assessed using hospital patient records and a self-complete resource use form, the client services receipt inventory (CSRI). The volume of resource use for each cost component will be measured directly in the trial from patient records and using the CSRI. A detailed economic evaluation analysis plan will be developed for approval by the TSC.

Discussion

ATTIRE is a UK multicentre trial that aims to evaluate the re-purposing of Human Albumin Solution (HAS) as an immune restorative drug. This protocol describes the RCT which will determine if administering daily 20% HAS infusions in order to increase serum albumin to near normal levels after admission to hospital will reduced rates of infection, renal failure and death in patients with decompensation of liver cirrhosis.

In liver cirrhosis current evidence based guidance[26-28] advocates the use of HAS in large volume paracentesis[29], hepatorenal syndrome[30] and spontaneous bacterial peritonitis [31,32]. The mechanism behind current usage of albumin is that of volume expansion. However many hepatologists believe albumin has other medicinal properties however a large-scale interventional clinical trial is yet to support this hypothesis. Ex vivo analysis from the ATTIRE feasibility study supported an immune restorative role for albumin[13], however it is unknown whether this correlates with clinical outcome. Our inclusion criteria are broad and designed to represent the decompensated cirrhotic population admitted to UK hospitals in order to make trial outcomes highly relevant in an era of soaring liver disease with few available treatment options. Preventing a liver cirrhosis related infection could potentially provide a bridge to liver transplantation, which represents the only curative option for these patients.

The design of this RCT has been refined based on the outcomes of an 80 patient interventional feasibility study[15]. Our targeted infusion protocol (table 2) was effective at increasing serum albumin, even in patients with a very low starting serum albumin. Many hepatology trials focus solely on 28-day survival however mortality requires large sample size and may be caused by other factors unrelated to infection. We considered that endpoints for interventions to prevent infection should be assessed at an earlier stage during the patient admission using ward based clinical measures and laboratory data. Multiple components of a primary composite endpoint were tested in our feasibility study[15]. Defining infection according to clinician diagnosis, triggering completion of an infection case report form, with a substantial proportion blindly scrutinized by a microbiology panel worked well in our feasibility study. Patients that developed renal dysfunction as assessed by an increase in serum creatinine had a poor prognosis, as expected. However only one patient developed cerebral dysfunction reflecting severe hepatic encephalopathy ($\geq$grade 3) suggesting under reporting and the majority of patients that solely triggered respiratory and cardiovascular endpoints had a good outcome. This is counterintuitive as organ dysfunction

For peer review only

is a key predictor of poor prognosis and we consider our data cast significant doubt over whether these dysfunctions can be recorded accurately in largely ward-based patients across multiple sites. This precludes their use as part of our RCT primary composite endpoint; although these will be reported.

Studies evaluating the safety of HAS infusions have generally shown it to be a safe treatment[29,33-35]. The main concerns in the cirrhotic population are related to volume overload leading to pulmonary oedema and increase in portal pressure leading to variceal bleeding. A recent interventional trial in septic decompensated patients reported an 8.3% rate of pulmonary oedema in the albumin treatment group[36]. However the weight based albumin dosing regimen on day 1 and 3 in this study led to much larger volumes of albumin prescribed than suggested in our protocol for these days. This and other studies in cirrhosis have not reported an increased incidence in variceal bleeding [29,32,37].

A challenge this study faces is the lack of a comparator infusion fluid in the control arm and hence the impossibility of site investigator and patient blinding. We had therefore considered comparison with another fluid such 0.9% sodium chloride however, this posed a number of problems. Physician preference for resuscitation fluid varies substantially and in preliminary discussions some objected to the additional salt load associated with 0.9% sodium chloride, in the non-albumin arm, if the patient did not require fluid resuscitation. Administration of 20% HAS for SBP, LVP and HRS is standard of care and therefore if patients in either arm developed these complications during their admission they would have to be unblinded to see if they were already receiving 20% HAS as part of the trial. As a significant number of patients do develop these complications, the consequent number of unblinded patients could have rendered the process invalid. Therefore the RCT had to be an open label study. This risks biasing adverse event reporting in the treatment arm, making albumin appear unsafe. We therefore felt that an open-label trial design versus a comparator arm of current standard of care was the only pragmatic solution. It could be argued that any improved outcome in the albumin treatment arm may simply be due to additional fluid resuscitation. The mean volume of 20% HAS infused over the treatment period (14 days) was 1litre in our feasibility study.

The regulation of HAS administration in the standard of care (control) arm poses a challenge as HAS is routinely used in clinical practice based on evidence based guidelines[26-28].The protocol allows use for these indications. If HAS is administered to control arm patients outside of current evidence based guidance this will be defined as a protocol deviation and the IDMC will be monitoring these events closely.

The ATTIRE RCT (n=866, up to 40 centres) began recruitment in April 2016 and aims to complete in December 2019. The medicinal properties of albumin in the hepatological community have long been questioned. The outcomes of this large, multicentre, randomised control trial will be highly relevant to improving the care of hospitalised patients with decompensation of chronic liver disease.

Ethics

This research group will involve a potentially vulnerable patient group that have hepatic encephalopathy and therefore lack the capacity to consent. Patients with encephalopathy are at high risk of infection and could be those that potentially receive maximal benefit from the intervention and therefore should not be denied access to the trial treatment. We have undertaken steps to ensure these patients are appropriately recruited to the trial (described in ‘Consent’ section) and provided individual site training.

For peer review only

Research Ethics positive opinion was given by the London-Brent Research Ethics Committee (ref: 15/LO/0104) which specialise in trials involving patients who lack the capacity to consent.The Clinical Trials Authorisation was issued by the Medicines and Healthcare products Regulatory Agency (MHRA, ref: 20363/0350/001-0001). The trial is registered with the European Medicines Agency (EudraCT 2014-002300-24) and has been adopted by the NIHR. Recruitment commenced in April 2016 and will finish by December 2018.

Data Sharing
Primary and secondary outcomes will be published in a peer reviewed journal as an open access paper. De-indentified participant data will be shared, as part of the trial results. The full study protocol and statistical analysis plans can be requested by emailing attire@ucl.ac.uk

Trial funding and Sponsor
The work is supported by the Health Innovation Challenge fund (Wellcome Trust and Department of Health) award number 164699. The trial sponsor is UCL with trial management activities conducted by the UCL Comprehensive Clinical Trials Unit.

<u>Trial management and monitoring</u>

Research Steering Group
The Research Steering Group (RSG) operates on behalf of the funders to ensure that appropriate milestones have been met in the delivery of the trial. It consists the CI, an independent expert and representatives of the Welcome Trust and Department of Health.

Trial Management Group
The Trial Management Group (TMG) comprises the CI, Clinical Research Fellow, Clinical Project Manager, Trial Statistician, Trial Manager, Data Manager, Health Economist and five trial site PIs. The TMG is responsible for developing the design, co-ordination and strategic management of the trial.

Trial Steering Committee
The Trial Steering Committee (TSC) is the independent group responsible for oversight of the trial in order to safeguard the interests of trial patients. The TSC provides advice to the CI, CTU, funder and sponsor on all aspects of the trial through its independent chair.

Independent Data Monitoring Committee
The Independent Data Monitoring Committee (IDMC) is responsible for safeguarding the interests of trial patients, monitoring the accumulating data and making recommendations to the TSC on whether the trial should continue as planned. It comprises a clinical chair (independent Hepatologist), independent Gastroenterologist and an independent statistician all with expertise in Clinical Trials.

<u>List of abbreviations used</u>
RCT: Randomised Control Trial
HAS: Human Albumin Solution
CRF: Case Report Form
HRS: Hepatorenal Syndrome
SBP: Spontaneous Bacterial Peritonitis
CI: Chief Investigator
PI: Principle Investigator
MDM: monocyte derived macrophage
LPS: lipopolysaccharide
TNFα: Tumour necrosis factor alpha

For peer review only

ICU: Intensive Care Unit
IV: intravenous

Competing interests
None

Authors' contributions
All authors read and approved the final manuscript.
LC: protocol development, writing of this manuscript
ZS, RH, SB, CT, RB: protocol development, protocol implementation, manuscript review
AM, NBS, DG: protocol development, scientific oversight
SS: protocol development, statistical oversight, manuscript review
KB: protocol development, statistical input, manuscript review
EF: protocol development, manuscript review
AOB: concept and design, protocol development, writing of this manuscript

Acknowledgements
We have had the support of the following individuals via trial oversight committees:
Professor Graeme Alexander
Professor Stephen Brett
Professor Mauro Bernardi
Professor Dominique Valla
Dr Vipul Jaraith
Mr Tim Clayton
Mr Brennan Kahan
Dr James O'Beirne
Dr Jim Portal
Dr Gavin Wright
Dr Ewan Forrest
Dr Stephen Ryder
Dr Yiannis Kallis
Dr Shahid Khan
Mr John Crookenden (Patient representative)
Mr Antonie Bakhuijsen (Patient representative)
Ms Susan Tebbs
A list of trial sites and PIs can be obtained by contacting attire@ucl.ac.uk

References

1. Health Service Quarterly. Vol. No. 40 (ed. Statistics, O.f.N.) p59-60 (Winter 2008).
2. Williams, R.*, et al.* Addressing liver disease in the UK: a blueprint for attaining excellence in health care and reducing premature mortality from lifestyle issues of excess consumption of alcohol, obesity, and viral hepatitis. *The Lancet* **384**, 1953-1997 (2014).
3. Williams, R.*, et al.* Disease burden and costs from excess alcohol consumption, obesity, and viral hepatitis: fourth report of the Lancet Standing Commission on Liver Disease in the UK. *Lancet* **391**, 1097-1107 (2018).
4. Fernandez, J.*, et al.* Bacterial infections in cirrhosis: epidemiological changes with invasive procedures and norfloxacin prophylaxis. *Hepatology* **35**, 140-148 (2002).
5. Rajkovic, I. & Williams, R. Abnormalities of neutrophil phagocytosis, intracellular killing and metabolic activity in alcoholic cirrhosis and hepatitis. *Hepatology* **6**, 252-262 (1986).
6. Acevedo, J. & Fernandez, J. New determinants of prognosis in bacterial infections in cirrhosis. *World journal of gastroenterology : WJG* **20**, 7252-7259 (2014).

For peer review only

7. Fernandez, J., *et al.* Prevalence and risk factors of infections by multiresistant bacteria in cirrhosis: a prospective study. *Hepatology* **55**, 1551-1561 (2012).
8. O'Brien, A.J., Welch, C.A., Singer, M. & Harrison, D.A. Prevalence and outcome of cirrhosis patients admitted to UK intensive care: a comparison against dialysis-dependent chronic renal failure patients. *Intensive care medicine* **38**, 991-1000 (2012).
9. O'Brien, A.J., *et al.* Immunosuppression in acutely decompensated cirrhosis is mediated by prostaglandin E2. *Nature medicine* **20**, 518-523 (2014).
10. Yang, J., Petersen, C.E., Ha, C.E. & Bhagavan, N.V. Structural insights into human serum albumin-mediated prostaglandin catalysis. *Protein science : a publication of the Protein Society* **11**, 538-545 (2002).
11. Klammt, S., *et al.* Albumin-binding function is reduced in patients with decompensated cirrhosis and correlates inversely with severity of liver disease assessed by model for end-stage liver disease. *Eur J Gastroenterol Hepatol.* **19**, 257-263 (2007).
12. Jalan, R., *et al.* Alterations in the functional capacity of albumin in patients with decompensated cirrhosis is associated with increased mortality. *Hepatology* **50**, 555-564 (2009).
13. China, L., *et al.* Albumin Counteracts Immune-Suppressive Effects of Lipid Mediators in Patients With Advanced Liver Disease. *Clinical gastroenterology and hepatology : the official clinical practice journal of the American Gastroenterological Association* (2017).
14. China, L., *et al.* ATTIRE: Albumin To prevenT Infection in chronic liveR failurE: study protocol for a single-arm feasibility trial. *BMJ Open* **6**, e010132 (2016).
15. China, L., *et al.* Administration of Albumin Solution Increases Serum Levels of Albumin in Patients With Chronic Liver Failure in a Single-Arm Feasibility Trial. *Clinical gastroenterology and hepatology : the official clinical practice journal of the American Gastroenterological Association* (2017).
16. Margarson MP, S.N. Serum albumin: touchstone or totem? *Anaesthesia* **53**, 789-803 (1998).
17. Kahan, B.C., Forbes, A.B., Dore, C.J. & Morris, T.P. A re-randomisation design for clinical trials. *BMC Med Res Methodol* **15**, 96 (2015).
18. Bajaj, J.S., *et al.* Second infections independently increase mortality in hospitalized patients with cirrhosis: the North American consortium for the study of end-stage liver disease (NACSELD) experience. *Hepatology* **56**, 2328-2335 (2012).
19. Jalan, R., *et al.* Development and validation of a prognostic score to predict mortality in patients with acute-on-chronic liver failure. *Journal of hepatology* **61**, 1038-1047 (2014).
20. Kim, T.Y., *et al.* Characteristics and Discrepancies in Acute-on-Chronic Liver Failure: Need for a Unified Definition. *PloS one* **11**, e0146745 (2016).
21. Bajaj, J.S., *et al.* Survival in infection-related acute-on-chronic liver failure is defined by extrahepatic organ failures. *Hepatology* **60**, 250-256 (2014).
22. Bajaj, J.S., *et al.* Linkage of gut microbiome with cognition in hepatic encephalopathy. *American journal of physiology. Gastrointestinal and liver physiology* **302**, G168-175 (2012).
23. Simon-Talero, M., *et al.* Effects of intravenous albumin in patients with cirrhosis and episodic hepatic encephalopathy: a randomized double-blind study. *Journal of hepatology* **59**, 1184-1192 (2013).
24. regulations, C. Medicines for Human Use (Clinical Trials Regulations) 2004 Informed consent in clinical trials. (ed. Authority, H.R.) (http://www.hra.nhs.uk/, 2008).

For peer review only

25. Angeli, P., *et al.* Diagnosis and management of acute kidney injury in patients with cirrhosis: Revised consensus recommendations of the International Club of Ascites. *Journal of hepatology* (2015).
26. European Association for the Study of the, L. EASL clinical practice guidelines on the management of ascites, spontaneous bacterial peritonitis, and hepatorenal syndrome in cirrhosis. *Journal of hepatology* **53**, 397-417 (2010).
27. Moore, K.P. & Aithal, G.P. Guidelines on the management of ascites in cirrhosis. *Gut* **55 Suppl 6**, vi1-12 (2006).
28. Runyon, B.A. & Aasld. Introduction to the revised American Association for the Study of Liver Diseases Practice Guideline management of adult patients with ascites due to cirrhosis 2012. *Hepatology* **57**, 1651-1653 (2013).
29. Bernardi, M., Caraceni, P., Navickis, R.J. & Wilkes, M.M. Albumin infusion in patients undergoing large-volume paracentesis: a meta-analysis of randomized trials. *Hepatology* **55**, 1172-1181 (2012).
30. Martin-Llahi, M., *et al.* Terlipressin and albumin vs albumin in patients with cirrhosis and hepatorenal syndrome: a randomized study. *Gastroenterology* **134**, 1352-1359 (2008).
31. Fernandez, J., *et al.* Effect of intravenous albumin on systemic and hepatic hemodynamics and vasoactive neurohormonal systems in patients with cirrhosis and spontaneous bacterial peritonitis. *Journal of hepatology* **41**, 384-390 (2004).
32. Sort P, N.M., Arroyo V, Aldeguer X, Planas R, Ruiz-del-Arbol L, Castells L, Vargas V, Soriano G, Guevara M, Ginès P, Rodés J. Effect of intravenous albumin on renal impairment and mortality in patients with cirrhosis and spontaneous bacterial peritonitis. *N Engl J Med.* **341**, 403-409 (1999).
33. Patel, A., Laffan, M.A., Waheed, U. & Brett, S.J. Randomised trials of human albumin for adults with sepsis: systematic review and meta-analysis with trial sequential analysis of all-cause mortality. *Bmj* **349**, g4561 (2014).
34. Caironi, P., *et al.* Albumin replacement in patients with severe sepsis or septic shock. *The New England journal of medicine* **370**, 1412-1421 (2014).
35. Reviewers, C.I.G.A. Human albumin administration in critically ill patients: systematic review of randomised controlled trials. *Bmj* **317**, 235-240 (1998).
36. Thevenot, T., *et al.* Effect of albumin in cirrhotic patients with infection other than spontaneous bacterial peritonitis. A randomized trial. *Journal of hepatology* **62**, 822-830 (2015).
37. Guevara, M., *et al.* Albumin for bacterial infections other than spontaneous bacterial peritonitis in cirrhosis. A randomized, controlled study. *Journal of hepatology* **57**, 759-765 (2012).

Figure 1. Overview of ATTIRE RCT Study Protocol

For peer review only

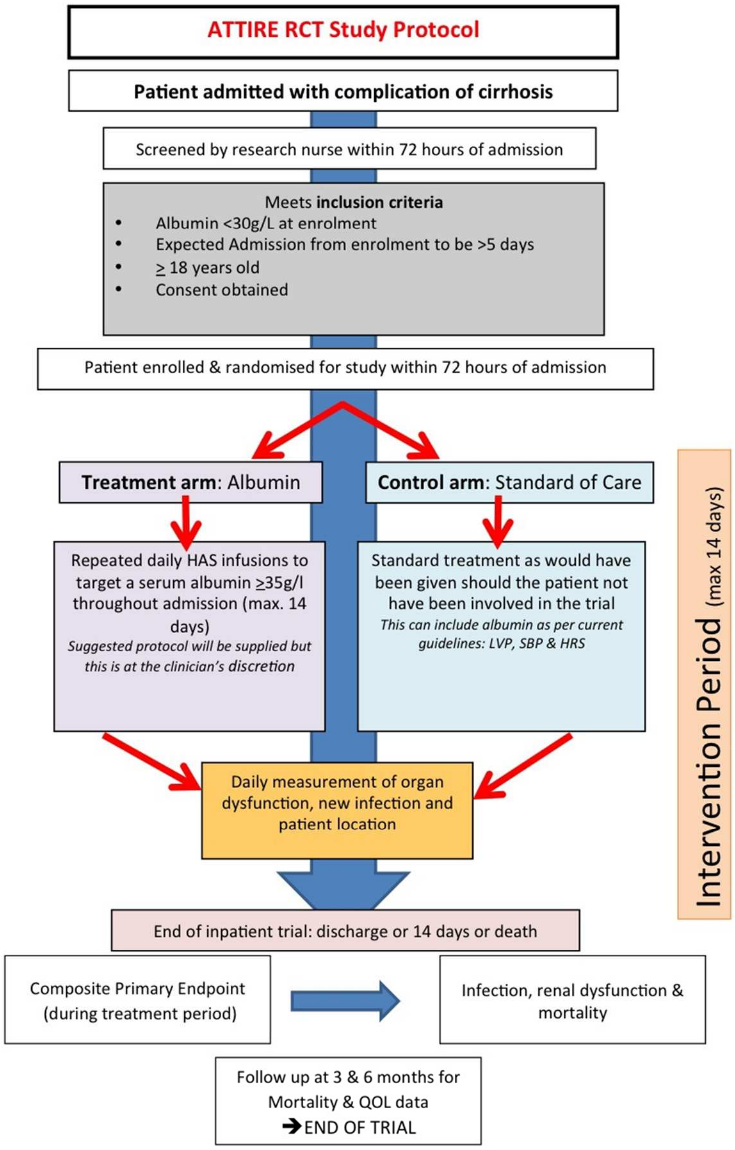

Figure 1. Overview of ATTIRE RCT Study Protocol

317x458mm (72 x 72 DPI)

Appendix 1 – Classification of infection

1. Spontaneous bacteraemia: positive blood cultures without a source of infection.
2. SBP: ascitic fluid polymorphonuclear cells >250 cells/mm^3
3. Lower respiratory tract infections: new pulmonary infiltrates in the presence of:
    i) at least one respiratory symptom (cough, sputum production, dyspnoea, pleuritic pain) with
    ii) at least one finding on auscultation (rales or crepitation) or one sign of infection (core body temperature >38°C or less than 36°C, shivering, or leukocyte count >10,000/mm3 or <4,000/mm3) in the absence of antibiotics.
4. Clostridium difficile Infection: diarrhoea with a positive C. difficile assay.
5. Bacterial entero-colitis: diarrhoea or dysentery with a positive stool culture for Salmonella, Shigella, Yersinia, Campylobacter,or pathogenic E. coli.
6. Soft-tissue/skin Infection: fever with cellulitis.
7. Urinary tract infection (UTI): urine white blood cell >15/high-power field with either positive urine gram stain or culture.
8. Intra-abdominal infections: diverticulitis, appendicitis, cholangitis, etc.
9. Other infections not covered above.
10. Fungal infections as a separate category.

For peer review only

Appendix 2 - Exploratory Objectives:

1. Effect of 20% HAS on the incidence, morbidity and mortality of the following infections:
    a. Bacterial
        i. Community acquired (diagnosed within 48 hours of admission in patients without previous hospitalisation in the last 6 months)
        ii. Nosocomial
        iii. Drug resistant pathogens
        iv. Culture negative sepsis (patients treated with antibiotics in the absence of microbiology evidence of infection)
        v. Clostridium Difficile infection
        vi. Source of infection (e.g. bacteraemia, respiratory, peritonitis, urine, skin etc.)
    b. Fungal
    c. Viral
2. Incidence, morbidity and mortality according to aetiology of Cirrhosis
3. Outcome for participants admitted to hospital for the first time with decompensated liver cirrhosis compared with participants who have previously been admitted to hospital with decompensated liver cirrhosis.
4. Requirement for ICU use of inotropes, vasopressors, hemofiltration and intubation and ventilation.
5. Analysis of samples from patients:
    a. Immune function studies using plasma samples – including but not limited to cell culture, qPCR, Albumin-ligand binding studies, western blot, flow cytometry and immunohistochemistry
    b. Plasma and urine lipidomic profile (in participants giving optional consent for urine samples)
6. Analysis of samples from patients providing additional optional consent (subject to further application for funding and ethical approval) :
    a. Stool microbiome analysis
    b. Genetic analysis of host response to infection
    c. Pathogen detection

For peer review only

Appendix 3: ATTIRE Patient Information Sheet (PIS)

ATTIRE: Stage 2

Albumin **T**o preven**T** **I**nfection in chronic live**R** failur**E**

The Problem

The most common cause of death in patients with liver disease is infection

➔ this is because in liver disease the immune system does not work well, especially when you are unwell in hospital.

What we know

Albumin (a protein found in your blood) is lower in patients with liver disease. Research looking at blood samples has suggested that increasing the albumin levels in the blood can potentially improve the immune system in patients with chronic liver disease.

What question is this study trying to answer?

1. Does albumin actually stop patients admitted to hospital with chronic liver disease from getting infections? (or help them fight them)

What additional treatment or investigations may occur if I take part?

If you decide to take part, you will be assigned to either the 'albumin group' or the 'standard of care group'. This allocation will be random.

1. In the 'standard of care group', you will receive exactly the same treatment as you would have done had you not been in the trial.
2. If in the 'albumin group' you will be given an intravenous infusion of albumin every day whilst you are in hospital **and** you will also receive all the other treatment that you would have done had you not been in the trial. Albumin is a very safe protein infusion that is already commonly used in liver patients who have kidney problems or ascites (fluid in the abdomen).
3. We will collect some blood samples (3 times) during your admission and if you are happy we will also collect urine (2) and stool sample (1). Where possible the blood will be taken when your other blood tests collection occurs to avoid any additional tests.

Throughout the trial you will be asked to complete some questionnaires; this will start when you enter the study and at 3 and 6 months after you are discharged from hospital. If you are happy we will collect one blood sample at either of these appointments.

What are the possible advantages?

You may not have any direct benefit from taking part in the study but you will be a part of a large study that will help to decide whether this is a beneficial treatment for patients with liver disease to help decrease infection.

What are the possible disadvantages?

You may be connected to a drip for 1-2 hours a day (this can be when other drip medication is given). The side effects of albumin are very rare (listed in detail in the following pages).

For peer review only - http://bmjopen.bmj.com/site/about/guidelines.xhtml

ATTIRE

Stage 2: Patient Information Sheet

Full study title:

Albumin **T**o preven**T** **I**nfection in chronic live**R** failur**E**.

A trial to investigate whether giving albumin to patients with advanced liver cirrhosis will reverse immune suppression and prevent infection.

Invitation to take part in research study

We would like to invite you to take part in a research trial. Before you decide we would like you to understand why the research is being done and what it involves. Please read this information sheet; one of the study team will answer any questions you may have. Talk to others about the study and ask us if there is anything that is not clear. Take time to decide whether or not you wish to take part.

The ATTIRE study is in two stages. The first stage included 80 patients and the second includes over 860 patients. They are very similar, but will look at slightly different outcomes.

This Patient Information Sheet is for Stage 2.

What is the purpose of the study?

Liver cirrhosis results in the liver tissue becoming scarred and developing lumps, these changes can prevent the liver from functioning properly. As the liver function worsens, symptoms such as fluid within the abdomen or legs, yellow skin, vomiting blood and confusion can develop. These are a result of advanced liver disease and are termed complications of cirrhosis. There are about 60,000 patient admissions to hospitals each year with such complications of liver cirrhosis. These patients are at higher risk of developing an infection during their hospital stay and this increase in risk is due to a weakened immune system. These infections can be very serious.

Giving fluid directly into the veins (intravenous) is known to be very important to help kidney function in patients with advanced liver disease. However, how much to give or indeed what is the best fluid to give is currently unknown. We have shown in laboratory testing, that albumin might be the best option as it can strengthen the immune system. However, it is unclear whether this will reduce the number of infections in hospital and that is what our trial wishes to investigate.

Albumin is found in the blood and is made in the liver. Patients with liver cirrhosis cannot make albumin as easily due to the damage to their liver. Albumin is already used to treat patients with liver cirrhosis and is known to be safe. In this stage of the study, patients will be given extra albumin to see if this helps their immune system to fight infections.

Why have I been chosen?

You are being asked to take part in this study as you have been admitted to hospital with complications of liver cirrhosis. Your doctor believes that you could be eligible to take part.

Do I have to take part?

It is up to you whether or not to take part in the study. If you decide not to take part, this will not affect the treatment you would normally receive. If you take part, you are free to withdraw at any time without giving a reason; this will not affect the care you receive.

What will happen to me if I take part?

For peer review only - http://bmjopen.bmj.com/site/about/guidelines.xhtml

If you take part in this study you will be either randomly allocated to the 'albumin group' or the 'standard of care group'.
If you are allocated to the 'albumin group', you will be given albumin solution as an intravenous fluid. Albumin has been shown to be safe in patients with liver disease. The amount of albumin that you are given will be decided by the amount that you already have in your blood. You will receive an infusion each day that you are in hospital, for a maximum of 14 days, until the levels in your blood have reached normal. You will also receive all of the other treatment that you would normally have had you not been in the study.
If you are in the 'standard of care group' you will have the treatment you would normally have, had you not been a part of the study, this may include albumin if you need it for another reason.
Regardless of which group you are in, you will have blood tests on most days while you are in hospital (as part of your normal care). At this time an additional sample (about 1 teaspoon) will be collected for up to 4 days during your time in the trial. You will also be asked to provide stool and urine samples while in the study, however this sample collection is optional and you can still take part in the study without providing these samples.
You also will be asked to complete some patient questionnaires during your time in the trial.
You will be contacted at 3 months and 6 months following your discharge from hospital to attend hospital visits, we will try to have these visits at the same time as your regular clinic appointments if possible.

What will I have to do?
If you agree to take part in the study you will be asked to sign a consent form. A member of the study team will then go through the initial assessments required for the study including collecting details of your medical history and any medication that you are currently taking. You will have tests (which will be part of your standard care) to look at your general health (e.g. heart rate and blood pressure), whether you have an infection, your liver function and the level of albumin already in your blood. Only if the level of albumin is below a certain level, will you be able to take part.

A research nurse will then come and record your blood tests and how well you are every day whilst you are in hospital. When you are discharged from hospital the research nurse will arrange to see you 3 months and 6 months after discharge to ask you some questions about how well you are feeling, this will be at the same time as your other clinic appointments if possible.

If you are a woman of child bearing potential, a pregnancy test will be carried out.

<u>**Starting Treatment**</u>

Once all of your tests have been completed and if you are still eligible to participate, a computer outside of the hospital will decide whether you will be in the 'albumin group' or the 'standard of care group', this is called randomisation. You will get exactly the same hospital care as you would have previously but with (or without) additional albumin infusions. This is so the trial investigators can see if there is an advantage, or disadvantage, of being given albumin.

The test for the amount of albumin in your blood will be used to work out how much albumin will be given, if you are in the 'albumin group', this will be given by a nurse or doctor. We expect the test to be performed anyway and so will still be done if you are in the 'standard of care group' as well. The albumin will be given directly into your blood through an intravenous cannula (a tube inserted into your vein to give fluid); the cannula will already be present as part of your standard care, an extra one will *not* be required. This will happen each day that you are on the study (a maximum of 14 days).

You will have blood tests on most days for liver function and to look for infections. These would normally be taken during your hospital stay. An extra blood sample (1 teaspoon) will be taken at the same time on three days throughout your admission while in the study and will be used to look at your immune system. You will also be asked to provide urine and stool samples whilst in the study, however these will be optional

For peer review only - http://bmjopen.bmj.com/site/about/guidelines.xhtml

samples. The stool samples and any samples left over will be stored for future research (for which further ethical committee review will be required).
If you do get an infection whilst in the study, you will be given the normal treatment for that type of infection that may include albumin. Taking part in the study will not affect the care that you normally get from your doctor.

End of study treatment

When you have taken part in the study for 14 days or your when your doctor feels you are ready to be discharged (if this is before 14 days of being on the study), you will stop your study treatments. This means that you will no longer be given albumin as part of the study or have any samples collected that are not part of your standard treatment. You will be asked to complete a questionnaire when you are discharged.
If you doctor feels that you still need albumin, you will continue to receive it.

The study team will then also contact you at 3 months and 6 months after you have been discharged from hospital, this will be at the same time as your other clinic appointments if possible. At these visits you will undergo tests that you would normally have as part of your standard of care and you will be asked to complete some questionnaires to see how you are feeling and what treatment you have had since being discharged from hospital. If you agree an extra blood sample (1 teaspoon, as above) will be taken at the same time as one of these appointments.

At the end of the study, if you agree, we will access information about you that is held by NHS digital, the Office of National Statistics (ONS) and other central NHS bodies regarding hospital admissions and mortality statistics for the six months following treatment. This is data that is collected as part of standard practice and will involve no additional involvement by you. The data, which is highly regulated, will be used to see if there are any long term effects of the treatment for you and the services that you may access.

When should I contact the study team?

You should contact a member of the study team or a doctor or nurse on your ward if you feel unwell at any time or have any incidents that affect your physical wellbeing. Contact details of the study team at your hospital and the Chief Investigator are available at the end of this leaflet.

What happens if I become pregnant?

All women of child-bearing potential will have a pregnancy test before entering the study. If you do become pregnant during treatment, you should inform your study team. It is not thought that albumin will affect pregnancy.

What are the alternative treatments if I don't take part?

If you do not take part in the study, standard treatment will be offered, which may include the use of albumin. Albumin is currently used for several reasons in patients with liver disease for example those who require extra fluid to support their kidneys, or to replace fluid that has been drained from the abdomen, or in those who develop infections.

What are the possible disadvantages and risks of taking part?

There are some possible risks and disadvantages that you should consider before taking part in this study:

- Wherever possible we will ensure that the blood tests needed for the study are taken at the same time as those needed for your routine care, but you may be asked to have additional samples taken if this is not possible. The risks of having a blood test include local bruising and discomfort.
- You may experience discomfort and bruising from fluid infusion.
- As the albumin is taken from human blood, in theory there is chance that you could get an infection (virus) from the person who has donated the blood. However there are no reports of patients getting viral infections from albumin that is made following European manufacturing standards. The albumin

For peer review only - http://bmjopen.bmj.com/site/about/guidelines.xhtml

given as part of the study is the same as that which would be given to you as part of your regular care by your doctor which you may have received as a treatment in the past.

- A cannula (very small plastic tube) will be used to give you the albumin. We would expect that you already will have one of these to give your other medications whilst in hospital and will use the same cannula. However if you don't have any medications or fluid that need to be given into the vein we will need to place a small cannula into the vein in order to give you the albumin.
- If in the albumin group you will be connected to an albumin infusion for around 1-2 hours a day. You can still move around the ward with this but will need to have a drip stand with you.

What are the possible benefits of taking part?
There may be no benefit to you from taking part, however, intravenous albumin may help your kidneys and raise blood pressure that can be low in patients with liver problems. Taking part may also provide additional information about patients with liver disease.

What are the side effects of any treatment received when taking part?
The side effects of albumin are rare but can include the following:

	Very Common	Common	Uncommon	Rare	Very Rare
Immune system disorders					Anaphylactic shock
Gastrointestinal disorders				Nausea	
Skin and subcutaneous tissue disorders				Flushing, skin rash	
General disorders and administration site conditions				Fever	

*__Very Common:__ in more than 1 in 10 patients treated; **Common:** in less than 1 in 10, but more than 1 in 100 patients treated; **Uncommon:** in less than 1 in 100, but more than 1 in 1000 patients; **Rare:** in less than 1 in 1000, but more than 1 in 10000 patients treated; **Very rare:** in less than 1 in 10000 patients treated, including isolated cases

Other side effects observed after placing Human Albumin on the market are: Hypersensitivity/Allergic reactions, Headache, Rapid heart beat, Abnormally low blood pressure, Breathlessness or breathing discomfort, Vomiting, Altered sense of taste, Hives, Itchiness, Chills, Heart attack, Irregular heart beat, Accumulation of fluid in the lung.

If any of the above reactions occur these will be treated as they would in normal standard care.

What if relevant new information becomes available?
Sometimes we get new information about the treatments being studied. If this happens, your study team will tell you about it and discuss how this may impact you. They will discuss whether you want to or should continue in the study.

What happens when the research study stops?
If you still require any treatment once the study stops, your doctor will prescribe the treatment most suitable for you.

Expenses and payments
You will not receive any payments for participating in this study.

For peer review only - http://bmjopen.bmj.com/site/about/guidelines.xhtml

What if something goes wrong?

If you have any concerns about the study, you should discuss them with your local study team first. You can also contact the Chief Investigator for the study, Dr Alastair O'Brien, on 020 7679 6851 who will be happy to discuss any of your concerns.

If you would like to discuss your concerns with someone not involved in the study, please contact your local Patient Advice and Liaison Service (PALS). Information on how to contact your local PALS can be found at the end of this information sheet.

Every care will be taken during this clinical study. However, in the unlikely event that you are injured by taking part, compensation may be available. If you suspect that the injury is the result of the Sponsor's (UCL) or the hospital's negligence then you may be able to claim compensation. After discussing it with your study team, please write to Dr Alastair O'Brien who is the Chief Investigator for the clinical study, about details of your claim. The Chief Investigator will then pass the claim to the Sponsor's Insurers, via the Sponsor's office. You may have to bear the costs of the legal action initially, and you should consult a lawyer about this. Contact details of the study team at your hospital and the Chief Investigator are available at the end of this leaflet.

You may also be able to claim compensation for injury caused by participation in this clinical study without the need to prove negligence on the part of University College London or another party. You should discuss this possibility with your study team in the same way as above.

Regardless of this, if you wish to complain, or have any concerns about any aspect of the way you have been approached or treated by members of staff or about any side effects (adverse events) you may have experienced due to your participation in the clinical study, the normal National Health Service complaints mechanisms are available to you. Please ask your study team if you would like more information on this. Details can also be obtained from the Department of Health website: http://www.dh.gov.uk.

Will my taking part in this study be kept confidential?

Yes, it will. We will request your approval to contact and inform your GP in the consent form about your participation in the study. We will send information relating to this research to the Comprehensive Clinical Trials Unit at University College London (UCL), where the study is being managed. Relevant information about you and all other patients in the study will be stored on password protected computers and in locked filing cabinets and will only be available to the staff working on the study. The information stored by the Comprehensive Clinical Trials Unit will contain a unique code, your initials, and month and year of birth. Your full name, address, contact details relevant medical history and relevant test results will be maintained at your local hospital. The information may be accessed by authorised personnel from UCL and the NHS hospital where you are being treated for auditing and monitoring purposes or by the regulatory authorities for inspection purposes to make sure the study is being carried out properly. Data collected during the study may be sent pseudo-anonymously (partially linked) to associated researchers in countries where the laws don't protect your privacy to the same extent as the law in the European Economic Area (EEA) but we will take all necessary steps to protect your privacy. Any data sent will only contain your unique code, your initials and month and year of birth. A data protection officer at UCL will be informed about any potential data transfer. You will be asked whether you agree to this when you sign the consent form.

If you withdraw consent from further study treatment, unless you object, your data and samples will remain on file and will be included in the final study analysis. In line with Good Clinical Practice guidelines, at the end of the study, your data will be securely archived for a minimum of 5 years. Arrangements for confidential destruction will then be made.

For peer review only - http://bmjopen.bmj.com/site/about/guidelines.xhtml

What will happen to the results of the research study?
If you choose to take part in the study, you will be given a unique trial identification code. All information collected from you for the study will be associated with this unique code, your initials, and month and year of birth. No other personal information will be sent to personnel outside your hospital and no one outside your hospital care team will be able to identify you from this information.

The blood, urine and stool samples collected for this study will be sent to the University College London, where they will be stored securely with your study specific identification number, initials, and month and year of birth. The blood and urine samples will be analysed at UCL to look at your immune system. The stool samples will be stored securely at UCL to be used for future research. Any future research carried out on the samples will be subject to further ethical committee review. It may be possible that the samples, or part of the samples, will be sent to countries outside the EEA where the laws for data protection differ from those in the UK. The urine and stool samples are optional samples and will not affect your participation in the study.

The Comprehensive Clinical Trials Unit will analyse the data collected on the patients in this stage of the study after all 866 patients have completed their treatment. The results of the study will be presented at a scientific conference and will be published in a scientific journal which will be accessible to the public. None of the research participants will be identified in any report or publication. Should you wish to see the results, or the publication, please ask your study team.

Who is conducting and funding the research?
This study is being managed by the Comprehensive Clinical Trials Unit at the University College London. Funding for the research has come from the Department of Health and Wellcome Trust.

Who has reviewed the study?
All research in the NHS is reviewed by a Research Ethics Committee and an agency of the Department of Health called the MHRA (the Medicines and Healthcare products Regulatory Agency). Both of these groups have confirmed that they are content for the study to go ahead and are regularly updated on its progress by the Comprehensive Clinical Trials Unit.

Who to contact for further information?
You are encouraged to ask any questions you wish, before, during or after your treatment. If you have any questions about the study, please speak to a member of the study team. If you wish to read the research on which this study is based, please ask your study team. If you require any further information or have any concerns while taking part in the study please contact one of the following people at your study site:

Doctor

Name *add name of PI* Tel. Number: *add Tel. number*

Study Nurse

Name *add name* Tel. Number: *add Tel. number*

PALS Add local PALS contact details

Or contact the Chief Investigator for the study:
Dr Alastair O'Brien at University College London, Tel. Number: 020 7 679 6851

Before you sign the informed consent form, you should ask questions about anything that you do not understand. The study staff will answer any questions before, during and after the study.

For peer review only - http://bmjopen.bmj.com/site/about/guidelines.xhtml

Thank you for taking the time to read this information sheet.

For peer review only

For peer review only - http://bmjopen.bmj.com/site/about/guidelines.xhtml

To be printed on hospital headed paper

ATTIRE

Stage 2: Patient Informed Consent Form

Albumin **To** prevenT **I**nfection in chronic liveR failurE (ATTIRE)

PATIENT ID: ☐☐☐-☐☐☐-☐☐   SITE: ☐☐☐

MONTH AND YEAR OF BIRTH: ☐☐☐-☐☐☐☐   PATIENT INITIALS: ☐☐☐

M M M - Y Y Y Y

For peer review only

This Informed Consent Form is intended for consenting patients into Stage 2 of the ATTIRE study.

Please insert your **initials** in the boxes to confirm consent:

	**Initials**
1. I confirm that I have read and understood the Patient Information Sheet version 5.0 dated 15Jan2018 for the ATTIRE study and have had the opportunity to consider the information and ask questions, which have been answered to my satisfaction.	
2. I understand that my participation is voluntary and that I am free to withdraw at any time, without giving a reason and without my medical care or legal rights being affected.	
3. I understand that sections of my medical records may be looked at by properly authorised personnel involved in the running of the study or from regulatory authorities where it is relevant to my taking part in this study. I give permission for these individuals to have access to my records.	
4. I agree to provide blood samples.	
5. I agree to take part in the ATTIRE study.	
6. I agree to be contacted by the research team 3 and 6 months after my hospital discharge.	

For peer review only - http://bmjopen.bmj.com/site/about/guidelines.xhtml

Optional Consent:

The following are optional. Declining to any of the below will not prevent you from taking part in the trial. Please initial in the boxes to confirm consent:	Initials
7. I give permission for my study data to be sent to an associated researcher outside of the EEA.	
8. I agree to provide urine samples.	
9. I agree to provide a stool sample.	
10. I agree to my remaining samples, not used in this study, being used in future, ethically approved, research.	
11. I understand that the information held and maintained by NHS Digital, the Office of National Statistics and other central UK NHS bodies may be used to provide information about my health.	
12. I give permission for my GP to be informed about my participation.	

Patient/ Legal Representative's Name (score through as applicable)	Signature	Date
Person Taking Consent	Signature	Date
Witness (if applicable)	Signature	Date

For peer review only - http://bmjopen.bmj.com/site/about/guidelines.xhtml

Appendix 4

	***Screening/* Day 1 (Randomisation)** *(within 72hrs of admission)*	***Treatment Period*** *(Day 2- Day 14)*	***End of Treatment***	***Discharge***	***Follow up*** *(3 & 6 months +/- 1 month)*[d]
Eligibility screen	X				
Informed consent	X				
Medical history	X				
Blood tests and sample collection*	X	X	X		X
Clinical observations recorded	X	X	X		
Randomisation	X				
Dosing with HAS/Standard of care	X[b]	X			
Infection/new (change) in antibiotics	X	X	X		
Patient location recorded	X	X	X	X	X
Concomitant Medication	X	X	X	X	X
Pregnancy test (βhCG) (women of child bearing potential only)	X				
Survival		X	X		X
CSRI Questionnaire	X[c]				X
EQ-5D-5L Questionnaire	X			X	X

*Blood test include: FBC, U&ES, LFTs, CRP, INR, Serum Albumin concentrations. From patients who provide additional consent blood will be collected at randomisation (day 1) and on days 5 and 10 +/- 2 days. Urine sample will be collected on day 1 and day 5 +/- 2 days and A stool sample will be collected once during the patients treatment period. A blood sample from patients who consent will be taken at either the 3 or 6 month follow up visit.

[b] If randomised to receive the HAS treatment the volume of HAS given will be determined by the Serum Albumin results (see section 6.4.1.2)

[c] The CRSI should be completed before randomisation. If this is not possible, the questionnaire should be completed as soon as the patient is able, prior to discharge. The questionnaire should be completed in relation to the three months prior to admission – details of the current hospital admission should not be included.

[d] If the patient has been re-randomised into the study, before completing the follow up visits for the previous randomisation; the patient should not undergo the follow up activities relating to that randomisation The follow up visits should only be conducted for the current randomisation.

BMJ Open

ATTIRE: Albumin To prevenT Infection in chronic liveR failurE: Study Protocol for an Interventional Randomised Controlled Trial

Journal:	*BMJ Open*
Manuscript ID	bmjopen-2018-023754.R2
Article Type:	Protocol
Date Submitted by the Author:	10-Sep-2018
Complete List of Authors:	China, Louise; UCL, Division of Medicine<br>Skene, Simon; University of Surrey, Surrey Clinical Trials Unit<br>Bennett, Kate; UCL, Comprehensive Clinical Trials Unit<br>Shabir, Zainib; UCL, Comprehensive Clinical Trials Unit<br>Hamilton, Roseanna; UCL, Comprehensive Clinical Trials Unit<br>Bevan, Scott; UCL, Comprehensive Clinical Trials Unit<br>Chandler, Torsten; UCL, Comprehensive Clinical Trials Unit<br>Maini, Alex; UCL, Division of Medicine<br>Becares, Natalia; UCL, Division of Medicine<br>Gilroy, Derek; UCL, Division of Medicine<br>Forrest, Ewan; NHS Greater Glasgow and Clyde, Glasgow Royal Infirmary<br>O'Brien, Alastair; UCL, Division of Medicine
**Primary Subject Heading**:	Gastroenterology and hepatology
Secondary Subject Heading:	Evidence based practice, Health economics, Research methods
Keywords:	Adult gastroenterology < GASTROENTEROLOGY, Hepatobiliary disease < GASTROENTEROLOGY, Gastroenterology < INTERNAL MEDICINE

SCHOLARONE™
Manuscripts

ATTIRE: Albumin To prevenT Infection in chronic liveR failurE: Study Protocol for an Interventional Randomised Controlled Trial

Louise China*[1], Simon S Skene[2], Kate Bennett[3], Zainib Shabir[3], Roseanna Hamilton[3], Scott Bevan[3], Torsten Chandler[3], Alexander A Maini[1], Natalia Becares[1], Derek Gilroy[1], Ewan Forrest[4], Alastair O'Brien[1]

[1] Division of Medicine, University College London, University Street, London
[2]School of Biosciences and Medicine, University of Surrey
[3] Comprehensive Clinical Trials Unit, UCL, Gower Street, London, WC1E 6BT
[4]Department of Gastroenterology, Glasgow Royal Infirmary, Castle Street, Glasgow G4 0SF

*Correspondence: louise.china@ucl.ac.uk
s.skene@surrey.ac.uk
k.bennett@ucl.ac.uk
z.shabir@ucl.ac.uk
roseanna.hamilton@ucl.ac.uk
a.maini@ucl.ac.uk
natalia.becares.13@ucl.ac.uk
d.gilroy@ucl.ac.uk
Ewan.Forrest@ggc.scot.nhs.uk
a.o'brien@ucl.ac.uk

For peer review only

Abstract

Introduction

Circulating Prostaglandin E_2 levels are elevated in acutely decompensated cirrhosis and have been shown to contribute to immune suppression. Albumin binds to and inactivates this immune-suppressive lipid mediator. Human Albumin Solution (HAS) could thus be repurposed as an immune restorative drug in these patients.

This is a phase III randomised controlled trial to verify whether targeting a serum albumin level of $\geq$35g/l in hospitalised patients with decompensated cirrhosis using repeated intravenous infusions of 20% HAS will reduce incidence of infection, renal dysfunction and mortality for the treatment period (maximum 14 days or discharge if <14 days) compared to standard medical care.

Methods and Analysis

ATTIRE stage 2 is a multicentre, open label, interventional randomised controlled trial. Patients with decompensated cirrhosis admitted to hospital with a serum albumin of <30g/L are eligible, subject to exclusion criteria. Patients randomised to intravenous HAS will have this administered, according to serum albumin levels, for up to 14 days or discharge. The infusion protocol aims to increase serum albumin to near normal levels.

The composite primary endpoint is: new infection, renal dysfunction or mortality within the trial treatment period. Secondary endpoints include mortality at up to 6months, incidence of other organ failures, cost effectiveness and QOL outcomes and time to liver transplant. The trial will recruit 866 patients at more than 30 sites across the United Kingdom.

Ethics & Dissemination

Research ethics approval was given by the London-Brent research ethics committee (ref: 15/LO/0104). The clinical trials authorisation was issued by the medicines and healthcare products regulatory agency (ref: 20363/0350/001-0001). The trial is registered with the European Medicines Agency (EudraCT 2014-002300-24) and has been adopted by the NIHR (ISRCTN 14174793). This manuscript refers to version 6.0 of the protocol. Results will be disseminated through peer reviewed journals and international conferences. Recruitment of the first participant occurred on 25 Jan 2016.

For peer review only

Strengths and limitations of this study

- This is the first prospective, interventional RCT with the primary aim of assessing the effects of **targeted** 20% Human Albumin Solution (HAS) infusions on infection, organ failure and death in hospitalised patients with decompensation of liver cirrhosis.
- Study design was optimised by a prior 80 patient feasibility study
- Cost effectiveness and quality of life assessment will review whether any outcomes are sustainable and feasible in a national health service setting
- The study is un-blinded to site investigators due to lack of agreement regarding the control infusion fluid and high cost required
- Patients in the control arm may receive 20% HAS as part of current international guidance as it would be unethical to withhold this in certain situations.

For peer review only

Introduction

Liver disease is an increasing cause of mortality currently in the UK and is the fifth most common cause of death[1]. These deaths are predicted to double over the next 20 years[2]. Mortality is mainly seen in the middle-aged group. Liver disease has surpassed lung and breast cancer as the leading cause of years of working life lost, and is set to overtake ischaemic heart disease within 2–3 years[3].

Patients with complications of liver failure secondary to liver cirrhosis are described as decompensated patients. They are highly prone to bacterial infection[4] secondary to immune dysfunction[5], with nosocomial (hospital-acquired) infection rates of 35% compared to five per cent in non-cirrhotic patients[6,7]. Of those who develop infection with organ dysfunction, 60-95% will die, often following prolonged intensive care unit admission[8]. There is, however, no medical strategy to restore immune competence.

It has been demonstrated that elevated circulating Prostaglandin E_2 (PGE_2) levels contribute to immune suppression in acutely decompensated patients[9]. The plasma protein albumin binds and catalyses the inactivation of PGE_2[10]. The binding capacity of endogenous albumin is known to be defective in cirrhosis[11,12] and improves following albumin infusions[13]. Albumin is synthesised in the liver and levels fall as the synthetic function of the liver declines in advanced cirrhosis. Therefore low albumin levels, with defective binding function, make PGE_2 more bioavailable. We found a serum albumin of < 30g/L predicted plasma induced macrophage dysfunction in a small cohort of decompensated cirrhosis patients[9] and this was reversed when albumin levels were increased to ≥30g/L. This finding has recently been validated in a multicentre clinical trial[13], however it is not known if these ex-vivo observations translate into an improvement in clinical outcomes.

We propose a novel strategy to repurpose 20% Human Albumin Solution (HAS) as an immune restorative drug in patients with decompensated liver cirrhosis with the aim of maintaining serum albumin at near normal levels.

The ATTIRE phase II single arm, multicentre feasibility trial[14] (n=80) verified that daily intravenous human albumin infusions restored serum albumin levels to near normal in

hospitalised decompensated cirrhosis patients, that it was safe and that there was physician equipoise prior to proceeding to a large RCT[15]. It has also allowed refinement of our definitions of RCT outcomes.

This phase III randomised controlled trial (RCT) (n=866) will assess the impact of 20% HAS treatment on the incidence of nosocomial infections, organ dysfunction and mortality in patients admitted to hospital with decompensation of liver cirrhosis.

Methods and Analysis

Primary Objective:

To verify whether raising and maintaining serum albumin levels ≥30g/L in patients admitted with decompensated cirrhosis using repeated 20% HAS infusions will reduce incidence of infection, renal dysfunction and mortality for the treatment period (maximum 14 days or discharge if <14 days) compared to standard medical care (figure 1). To raise and maintain serum albumin levels above 30g/L, 20% HAS will be prescribed and administered in the intervention arm if levels are below 35g/L.

Secondary Objectives are to assess the impact of the intervention on development of other organ dysfunction (cardiac, respiratory, brain and liver), cost effectiveness; quality of life and sustainability of any outcomes over a 6 month follow up period.

Trial Design

This is a multicentre, open label, randomized controlled trial in which patients will either be treated with 20% HAS to raise and maintain serum albumin above 30g/L or will continue to receive their usual standard of care treatment. 866 sequential patients admitted to 30 UK participating hospitals with a clinical diagnosis of cirrhosis and decompensation will be screened using the inclusion and exclusion criteria (Table 1). Decompensation includes: jaundice, ascites, hepatic encephalopathy, variceal bleeding, coagulopathy and hepatorenal syndrome.

Patient Inclusion Criteria	Patient Exclusion Criteria
All patients admitted to hospital with acute onset or worsening of complications of cirrhosis	Advanced hepatocellular carcinoma with life expectancy of less than 8 weeks
Over 18 years of age	Patients who will receive palliative treatment only during their hospital admission
Predicted hospital admission ≥ 5 days at trial enrolment, which must be within 72 hours of admission	Patients who are pregnant
Serum albumin <30g/l at screening	Known or suspected severe cardiac dysfunction
Documented informed consent to participate (or consent given by a legal representative)	Any clinical condition which the investigator considers would make the patient unsuitable for the trial
	The patient has been involved in a clinical trial of Investigational Medicinal Products (IMPs) within the previous 30 days that would impact on their participation in this study
	Trial investigators unable to identify the patient (by NHS number)

Table 1. Patient Selection Criteria

For peer review only

As advanced liver disease requires frequent hospital re-admissions, patients may be enrolled in the RCT more than once, with a 30 day "washout period" following discharge after the first enrolment, during which time they cannot be enrolled for a second time. This is to account for albumin's half-life of 18-21 days[16]. Patients re-entering the trial in this way will be re-randomised, so that each enrolment will be considered as an independent patient 'presentation'[17].

To ensure homogeneity in the approach to patient recruitment, intervention and data collection all sites received introduction training and regular follow up re-training plus monitoring and support visits from the sponsor.

Clinical trial endpoints

Primary endpoint

A composite endpoint comprising incidence of infection, renal dysfunction and mortality within the treatment period (for a maximum of 14 days OR when the patient is considered fit for discharge if < 14 days).

The three components of the composite endpoint are:

1. New Infection: indicated by clinician diagnosis and clinical evidence provided on completed infection case report form (CRF). Blinded data from a clinically representative sample of patients will be scrutinised for the presence of infection by a Clinical Trial Endpoint Review Committee. The committee will contain microbiologists who will validate the presence of infection according to peer reviewed criteria[18] (Appendix 1).
2. Renal Dysfunction: a serum creatinine increase of ≥50% as compared to serum creatinine at randomisation OR the patient initiated on renal replacement support (either haemodialysis or haemofiltration) OR a rise in serum creatinine of ≥26.5 µmol/L within 48hours.
3. Mortality

Primary endpoint data will be recorded throughout the treatment period; however only contributing events captured on the treatment day 3 case report form (CRF) through to the day 15 CRF (the end of treatment examination day) will contribute to the primary outcome. This allows the biological effect of albumin to be established at least 24 hours following initiation of treatment.

If the participant is discharged or deemed medically fit for discharge prior to day 15, no further primary outcome data will be measured after this date, as this will signal the end of the participant's treatment period.

Secondary Endpoints:

1. Mortality at 28 days post randomisation and at 3 & 6 months post discharge
2. Time to outcome (first event of infection/renal dysfunction/death)
3. Incidence of respiratory, circulatory and cerebral dysfunction during the treatment period (based on modified components of the CLIF-SOFA[19] score).
4. Incidence of liver transplant within six months of treatment
5. Total amount of HAS administered during the treatment period
6. Duration of hospital stay
7. Change in prognostic score (assessed by MELD) between randomisation and end of treatment period.

For peer review only

8. Days in ICU during treatment period
9. Incremental cost and cost-effectiveness up to 6 months post discharge
10. Impact on quality of life (QOL) up to 6 months post discharge
11. Safety and tolerability of HAS as indicated by Serious Adverse Events (SAEs)
12. Use of terlipressin for (a) renal dysfunction, (b) hypotension, (c) variceal bleeding.

Patients in the RCT will be followed up 3 and 6 months (+/- 1 month) following discharge from hospital. Exploratory objectives are listed in Appendix 2.

Patient population

This will include all patients admitted to hospital with the complications of decompensated liver cirrhosis and serum albumin < 30 g/L, aged over 18 years with anticipated hospital length of stay of 5 or more days at trial enrolment, which should be no later than 72 hours from admission. This is subject to exclusion criteria as detailed in table 1. The diagnosis of cirrhosis will be made by the clinical team as per standard UK practice and does not require liver biopsy or imaging. Acute decompensation of liver cirrhosis associated with organ failures is termed ACLF (Acute on Chronic Liver Failure). ACLF has a number of definitions[20,21] based on the SOFA (sequential organ failure assessment) score, all are associated with a poor prognosis. This study will include patients with decompensated cirrhosis with and without ACLF, it will also record the development of ACLF during the study treatment period.

Consent

Patient information sheets (Appendix 3) will be given to and discussed with potential patients before consent is sought. Informed consent will be obtained from each participant or his or her legal representative. Patients who lack mental capacity, for any reason, are not excluded from the trial. An important subgroup of patients will have hepatic encephalopathy and these patients may lack capacity to consent, however these patients may be amongst those that receive maximum benefit from the intervention[21-23]. In this case consent will be sought from an appropriate legal representative independent of the research team as per current UK clinical trials regulations[24].

Intervention

After randomisation (when serum albumin is <30g/L) patients will receive either daily dose of 20% HAS intravenously if their serum albumin level is less than 35g/L (at approximately 100mls/hour) or standard medical care (which may include 20% HAS infusions for indications listed in established guidelines only – see below) for a maximum of 14 days or discharge (if < 14 days). The volume of HAS each day will be determined by the patient's serum albumin level on that day (or the closest previous measurement if there are no results from that day available).

Table 2 shows the suggested dosing protocol for albumin administration in the treatment arm group. This is a suggested regimen and responsible clinicians are given flexibility to alter this depending on the clinical situation. The effectiveness of this protocol, and approach, was verified in the ATTIRE feasibility study[15]. Differing regimens may be used to cover large volume paracentesis (8g of albumin per litre of ascites drained) or treat Hepatorenal syndrome (1g of albumin per kilogram of body weight) as per international guidelines[25,26] but HAS *must* be prescribed and given if serum albumin <35g/L unless there are any safety concerns**.** All variations will be recorded in the patient's daily Case Report Form (CRF).

Patient's Serum Albumin Level	Amount of 20% HAS to be administered
≥35 g/L	none
30-34 g/L	100mls
26-29 g/L	200mls
20-25 g/L	300mls
<20 g/L	400mls

Table 2. Treatment arm dosing protocol for 20% HAS administration (amounts per day) as advised by measured serum albumin level on that day.

20% HAS will only be given in the Standard of Care arm if the patient requires large volume paracentesis or has SBP or HRS (as per established guidelines[26-28]). This needs to be clearly recorded in the patient's CRF and if HAS is given for any other indication in the Standard of Care arm this will be considered a protocol deviation. The administration of HAS in the Standard of Care arm will be closely monitored by the Independent Data Monitoring Committee (IDMC).

Randomisation will use a minimisation algorithm incorporating a random element, stratifying by centre, MELD score, and number of organ dysfunctions, serum albumin level and if antibiotics are currently being prescribed. To ensure maximum balance is achieved across the stratification factors, minimisation will be carried out on these factors separately.

Evaluations during and after treatment

Clinical, biochemical, microbiological, health economic and quality of life data will be collected during the trial treatment period (see Appendix 4) using information from hospital notes that is recorded as standard of care. There is follow up at 3 and 6 months. The blood, urine and stool samples collected for immune function tests will be analysed in a blinded fashion at a central site.

Patient and Public Involvement

Patients who have had a previous diagnosis of liver cirrhosis were involved in the development of the research question and outcome measures. Patient feedback led to the addition of quality of life evaluation. The study protocol and patient information leaflets were reviewed by our patient representatives with positive feedback. In addition an independent patient representative sat on the ethics approval panel. Results will be disseminated to patients via open access publication and our local trials teams.

Statistical considerations

Sample Size

A 30% incidence of nosocomial infection in decompensated cirrhosis patients is well documented with up to 30% of these patients developing organ dysfunction[21,29] and an overall mortality of 38% at 1 month[18,21,29]. These figures are supportive of 30% as a conservative estimate for the composite primary endpoint of incidence of new infection, renal dysfunction or mortality up to 14 days from randomisation.

We have assumed that the "immune-restorative" albumin treatment would reduce this rate by 30% to a rate of 21%, which would be considered clinically relevant. 389 patients per arm would be sufficient to detect such a difference with 80% power at a significance level of 0.05. Allowing for loss-to-follow-up/withdrawal of 10% from the trial, we will aim to recruit 433 to each arm (866 in total).

For peer review only

Statistical Evaluation

A detailed statistical analysis plan (SAP) will be written and approved by the Trial Steering Committee before the substantive analysis of unblinded trial data.

Baseline characteristics will be summarised by treatment using appropriate descriptive statistics; means and standard deviations for approximately normally distributed variables, medians and interquartile ranges for non-normally distributed variables and counts (percentages) for categorical variables.

Primary outcome

The primary outcome is the difference in event rates, according to treatment, of the composite endpoint of infection, renal dysfunction and mortality within the intervention period (from ≥ 24 hours from the start of treatment/randomisation up to a maximum of fourteen days or up to discharge if this is prior to fourteen days).

Since the primary outcome has a binary classification, logistic regression will be used to determine whether there is any difference in rates due to treatment, by inclusion of a binary covariate indicating treatment. The results will be adjusted for pre-determined prognostic factors used as stratifying variables in the randomisation, which shall be included as additional covariates in the model. The model coefficient due to treatment will give an estimate of the difference in log odds, or equivalently (exponentiated to give) an estimate of the odds multiplier, i.e. the change in odds of a negative outcome on the composite endpoint due to treatment with albumin. It is expected that this effect will be negative, so that treatment with albumin is seen to reduce the odds of a negative outcome. A reduction from 30% to 21% would be associated with a reduction in odds of around 38%. Predicted probabilities will be presented for the composite outcome for each of the treatment arms, adjusted for the model covariates.

The effect of treatment on the individual components, infection, renal dysfunction and mortality will be reported separately.

Secondary and exploratory outcomes

Secondary and exploratory outcomes will be handled similarly. Continuous secondary outcome measures will be analysed using a linear regression model, and time-to-event outcomes using Kaplan Meier plots and Cox's proportional hazards models where covariate adjustment is necessary.

All statistical tests will use a 2-sided p-value of 0.05, unless otherwise specified, and all confidence intervals presented will be 95% and two-sided. All statistical analysis will be performed using Stata (StataCorp, College Station, TX, USA).

Economic Evaluation

The economic evaluation will consist of two parts. The primary analysis will be a decision analytic model comparing the expected lifetime costs and benefits of treatment reported in terms of the cost per incremental quality adjusted life years (QALYs) gained. QALYs will be calculated based on the health related quality of life (HRQL) and mortality data collected during the trial. HRQL will be measured according to the EQ-5D-5L (www.euroqol.org), which we will collect at baseline, discharge, 3 months and 6 months (+/- 1 month) for each individual patient. If patients are re-randomised they will have separate follow up events for each episode of re-randomisation, unless the patient is re-randomised within the follow up phase of the study. Utility scores will be calculated using UK-specific tariffs and adjusting for

For peer review only

baseline differences in patients in the trial arms if necessary. These will be extrapolated over the expected lifetime of the patient and where necessary will draw on the existing literature. The final form of the decision analytic model will be determined during the trial period. Appropriate methods for estimating changes in health over time, such as state-transition models, will be given consideration.

The secondary economic analysis will be a 'within-trial' analysis, reporting on the incremental cost and benefits observed during the trial period and follow-up periods. The cost-effectiveness measures in the within-trial analysis will be the incremental cost per infection avoided and the incremental cost per QALY gained. Costs will be measured as described below. Infections avoided will be derived from the primary outcome in the trial. Patient resource use will be assessed using hospital patient records and a self-complete resource use form, the client services receipt inventory (CSRI). The volume of resource use for each cost component will be measured directly in the trial from patient records and using the CSRI. A detailed economic evaluation analysis plan will be developed for approval by the TSC.

Discussion

ATTIRE is a UK multicentre trial that aims to evaluate the re-purposing of Human Albumin Solution (HAS) as an immune restorative drug. This protocol describes the RCT which will determine if administering daily 20% HAS infusions in order to increase serum albumin to near normal levels after admission to hospital will reduced rates of infection, renal failure and death in patients with decompensation of liver cirrhosis.

In liver cirrhosis current evidence based guidance[26-28] advocates the use of HAS in large volume paracentesis[30], hepatorenal syndrome[31] and spontaneous bacterial peritonitis [32,33]. The mechanism behind current usage of albumin is that of volume expansion. However many hepatologists believe albumin has other medicinal properties however a large-scale interventional clinical trial is yet to support this hypothesis. Ex vivo analysis from the ATTIRE feasibility study supported an immune restorative role for albumin[13], however it is unknown whether this correlates with clinical outcome. Our inclusion criteria are broad and designed to represent the decompensated cirrhotic population admitted to UK hospitals in order to make trial outcomes highly relevant in an era of soaring liver disease with few available treatment options. Preventing a liver cirrhosis related infection could potentially provide a bridge to liver transplantation, which represents the only curative option for these patients.

The design of this RCT has been refined based on the outcomes of an 80 patient interventional feasibility study[15]. Our targeted infusion protocol (table 2) was effective at increasing serum albumin, even in patients with a very low starting serum albumin. Many hepatology trials focus solely on 28-day survival however mortality requires large sample size and may be caused by other factors unrelated to infection. We considered that endpoints for interventions to prevent infection should be assessed at an earlier stage during the patient admission using ward based clinical measures and laboratory data. Multiple components of a primary composite endpoint were tested in our feasibility study[15]. Defining infection according to clinician diagnosis, triggering completion of an infection case report form, with a substantial proportion blindly scrutinized by a microbiology panel worked well in our feasibility study. Patients that developed renal dysfunction as assessed by an increase in serum creatinine had a poor prognosis, as expected. However only one patient developed cerebral dysfunction reflecting severe hepatic encephalopathy ($\geq$grade 3) suggesting under reporting and the majority of patients that solely triggered respiratory and cardiovascular endpoints had a good outcome. This is counterintuitive as organ dysfunction

For peer review only

is a key predictor of poor prognosis and we consider our data cast significant doubt over whether these dysfunctions can be recorded accurately in largely ward-based patients across multiple sites. This precludes their use as part of our RCT primary composite endpoint; although these will be reported.

Studies evaluating the safety of HAS infusions have generally shown it to be a safe treatment[30,34-36]. The main concerns in the cirrhotic population are related to volume overload leading to pulmonary oedema and increase in portal pressure leading to variceal bleeding. A recent interventional trial in septic decompensated patients reported an 8.3% rate of pulmonary oedema in the albumin treatment group[37]. However the weight based albumin dosing regimen on day 1 and 3 in this study led to much larger volumes of albumin prescribed than suggested in our protocol for these days. This and other studies in cirrhosis have not reported an increased incidence in variceal bleeding [30,33,38].

A challenge this study faces is the lack of a comparator infusion fluid in the control arm and hence the impossibility of site investigator and patient blinding. We had therefore considered comparison with another fluid such 0.9% sodium chloride however, this posed a number of problems. Physician preference for resuscitation fluid varies substantially and in preliminary discussions some objected to the additional salt load associated with 0.9% sodium chloride, in the non-albumin arm, if the patient did not require fluid resuscitation. Administration of 20% HAS for SBP, LVP and HRS is standard of care and therefore if patients in either arm developed these complications during their admission they would have to be unblinded to see if they were already receiving 20% HAS as part of the trial. As a significant number of patients do develop these complications, the consequent number of unblinded patients could have rendered the process invalid. Therefore the RCT had to be an open label study. This risks biasing adverse event reporting in the treatment arm, making albumin appear unsafe. We therefore felt that an open-label trial design versus a comparator arm of current standard of care was the only pragmatic solution. It could be argued that any improved outcome in the albumin treatment arm may simply be due to additional fluid resuscitation. The mean volume of 20% HAS infused over the treatment period (14 days) was 1litre in our feasibility study.

The regulation of HAS administration in the standard of care (control) arm poses a challenge as HAS is routinely used in clinical practice based on evidence based guidelines[26-28].The protocol allows use for these indications. If HAS is administered to control arm patients outside of current evidence based guidance this will be defined as a protocol deviation and the IDMC will be monitoring these events closely.

The ATTIRE RCT (n=866, up to 40 centres) began recruitment in April 2016 and aims to complete in December 2019. The medicinal properties of albumin in the hepatological community have long been questioned. The outcomes of this large, multicentre, randomised control trial will be highly relevant to improving the care of hospitalised patients with decompensation of chronic liver disease.

Ethics

This research group will involve a potentially vulnerable patient group that have hepatic encephalopathy and therefore lack the capacity to consent. Patients with encephalopathy are at high risk of infection and could be those that potentially receive maximal benefit from the intervention and therefore should not be denied access to the trial treatment. We have undertaken steps to ensure these patients are appropriately recruited to the trial (described in ‘Consent’ section) and provided individual site training.

For peer review only

Research Ethics positive opinion was given by the London-Brent Research Ethics Committee (ref: 15/LO/0104) which specialise in trials involving patients who lack the capacity to consent.The Clinical Trials Authorisation was issued by the Medicines and Healthcare products Regulatory Agency (MHRA, ref: 20363/0350/001-0001). The trial is registered with the European Medicines Agency (EudraCT 2014-002300-24) and has been adopted by the NIHR. Recruitment commenced in April 2016 and will finish by December 2018.

Data Sharing

Primary and secondary outcomes will be published in a peer reviewed journal as an open access paper. De-indentified participant data will be shared, as part of the trial results. The full study protocol and statistical analysis plans can be requested by emailing attire@ucl.ac.uk

Trial funding and Sponsor

The work is supported by the Health Innovation Challenge fund (Wellcome Trust and Department of Health) award number 164699. The trial sponsor is UCL with trial management activities conducted by the UCL Comprehensive Clinical Trials Unit.

Trial management and monitoring

Research Steering Group

The Research Steering Group (RSG) operates on behalf of the funders to ensure that appropriate milestones have been met in the delivery of the trial. It consists the CI, an independent expert and representatives of the Welcome Trust and Department of Health.

Trial Management Group

The Trial Management Group (TMG) comprises the CI, Clinical Research Fellow, Clinical Project Manager, Trial Statistician, Trial Manager, Data Manager, Health Economist and five trial site PIs. The TMG is responsible for developing the design, co-ordination and strategic management of the trial.

Trial Steering Committee

The Trial Steering Committee (TSC) is the independent group responsible for oversight of the trial in order to safeguard the interests of trial patients. The TSC provides advice to the CI, CTU, funder and sponsor on all aspects of the trial through its independent chair.

Independent Data Monitoring Committee

The Independent Data Monitoring Committee (IDMC) is responsible for safeguarding the interests of trial patients, monitoring the accumulating data and making recommendations to the TSC on whether the trial should continue as planned. It comprises a clinical chair (independent Hepatologist), independent Gastroenterologist and an independent statistician all with expertise in Clinical Trials.

List of abbreviations used

RCT: Randomised Control Trial
HAS: Human Albumin Solution
CRF: Case Report Form
HRS: Hepatorenal Syndrome
SBP: Spontaneous Bacterial Peritonitis
CI: Chief Investigator
PI: Principle Investigator
MDM: monocyte derived macrophage
LPS: lipopolysaccharide
TNFα: Tumour necrosis factor alpha

For peer review only

ICU: Intensive Care Unit
IV: intravenous

Competing interests
None

Authors' contributions
All authors read and approved the final manuscript.
LC: protocol development, writing of this manuscript
ZS, RH, SB, CT: protocol development, protocol implementation, manuscript review
AM, NBS, DG: protocol development, scientific oversight
SS: protocol development, statistical oversight, manuscript review
KB: protocol development, statistical input, manuscript review
EF: protocol development, manuscript review
AOB: concept and design, protocol development, writing of this manuscript

Acknowledgements
We have had the support of the following individuals via trial oversight committees:
Professor Graeme Alexander
Professor Stephen Brett
Professor Mauro Bernardi
Professor Dominique Valla
Dr Vipul Jaraith
Mr Tim Clayton
Mr Brennan Kahan
Dr James O'Beirne
Dr Jim Portal
Dr Gavin Wright
Dr Ewan Forrest
Dr Stephen Ryder
Dr Yiannis Kallis
Dr Shahid Khan
Mr John Crookenden (Patient representative)
Mr Antonie Bakhuijsen (Patient representative)
Ms Susan Tebbs
A list of trial sites and PIs can be obtained by contacting attire@ucl.ac.uk

References

1. Health Service Quarterly. Vol. No. 40 (ed. Statistics, O.f.N.) p59-60 (Winter 2008).
2. Williams, R.*, et al.* Addressing liver disease in the UK: a blueprint for attaining excellence in health care and reducing premature mortality from lifestyle issues of excess consumption of alcohol, obesity, and viral hepatitis. *The Lancet* **384**, 1953-1997 (2014).
3. Williams, R.*, et al.* Disease burden and costs from excess alcohol consumption, obesity, and viral hepatitis: fourth report of the Lancet Standing Commission on Liver Disease in the UK. *Lancet* **391**, 1097-1107 (2018).
4. Fernandez, J.*, et al.* Bacterial infections in cirrhosis: epidemiological changes with invasive procedures and norfloxacin prophylaxis. *Hepatology* **35**, 140-148 (2002).
5. Rajkovic, I. & Williams, R. Abnormalities of neutrophil phagocytosis, intracellular killing and metabolic activity in alcoholic cirrhosis and hepatitis. *Hepatology* **6**, 252-262 (1986).
6. Acevedo, J. & Fernandez, J. New determinants of prognosis in bacterial infections in cirrhosis. *World journal of gastroenterology : WJG* **20**, 7252-7259 (2014).

For peer review only

7. Fernandez, J., *et al.* Prevalence and risk factors of infections by multiresistant bacteria in cirrhosis: a prospective study. *Hepatology* **55**, 1551-1561 (2012).
8. O'Brien, A.J., Welch, C.A., Singer, M. & Harrison, D.A. Prevalence and outcome of cirrhosis patients admitted to UK intensive care: a comparison against dialysis-dependent chronic renal failure patients. *Intensive care medicine* **38**, 991-1000 (2012).
9. O'Brien, A.J., *et al.* Immunosuppression in acutely decompensated cirrhosis is mediated by prostaglandin E2. *Nature medicine* **20**, 518-523 (2014).
10. Yang, J., Petersen, C.E., Ha, C.E. & Bhagavan, N.V. Structural insights into human serum albumin-mediated prostaglandin catalysis. *Protein science : a publication of the Protein Society* **11**, 538-545 (2002).
11. Klammt, S., *et al.* Albumin-binding function is reduced in patients with decompensated cirrhosis and correlates inversely with severity of liver disease assessed by model for end-stage liver disease. *Eur J Gastroenterol Hepatol.* **19**, 257-263 (2007).
12. Jalan, R., *et al.* Alterations in the functional capacity of albumin in patients with decompensated cirrhosis is associated with increased mortality. *Hepatology* **50**, 555-564 (2009).
13. China, L., *et al.* Albumin Counteracts Immune-Suppressive Effects of Lipid Mediators in Patients With Advanced Liver Disease. *Clinical gastroenterology and hepatology : the official clinical practice journal of the American Gastroenterological Association* (2017).
14. China, L., *et al.* ATTIRE: Albumin To prevenT Infection in chronic liveR failurE: study protocol for a single-arm feasibility trial. *BMJ Open* **6**, e010132 (2016).
15. China, L., *et al.* Administration of Albumin Solution Increases Serum Levels of Albumin in Patients With Chronic Liver Failure in a Single-Arm Feasibility Trial. *Clinical gastroenterology and hepatology : the official clinical practice journal of the American Gastroenterological Association* (2017).
16. Margarson MP, S.N. Serum albumin: touchstone or totem? *Anaesthesia* **53**, 789-803 (1998).
17. Kahan, B.C., Forbes, A.B., Dore, C.J. & Morris, T.P. A re-randomisation design for clinical trials. *BMC Med Res Methodol* **15**, 96 (2015).
18. Bajaj, J.S., *et al.* Second infections independently increase mortality in hospitalized patients with cirrhosis: the North American consortium for the study of end-stage liver disease (NACSELD) experience. *Hepatology* **56**, 2328-2335 (2012).
19. Jalan, R., *et al.* Development and validation of a prognostic score to predict mortality in patients with acute-on-chronic liver failure. *Journal of hepatology* **61**, 1038-1047 (2014).
20. Kim, T.Y., *et al.* Characteristics and Discrepancies in Acute-on-Chronic Liver Failure: Need for a Unified Definition. *PloS one* **11**, e0146745 (2016).
21. Bajaj, J.S., *et al.* Survival in infection-related acute-on-chronic liver failure is defined by extrahepatic organ failures. *Hepatology* **60**, 250-256 (2014).
22. Bajaj, J.S., *et al.* Linkage of gut microbiome with cognition in hepatic encephalopathy. *American journal of physiology. Gastrointestinal and liver physiology* **302**, G168-175 (2012).
23. Simon-Talero, M., *et al.* Effects of intravenous albumin in patients with cirrhosis and episodic hepatic encephalopathy: a randomized double-blind study. *Journal of hepatology* **59**, 1184-1192 (2013).
24. regulations, C. Medicines for Human Use (Clinical Trials Regulations) 2004 Informed consent in clinical trials. (ed. Authority, H.R.) (http://www.hra.nhs.uk/, 2008).

For peer review only

25. Angeli, P.*, et al.* Diagnosis and management of acute kidney injury in patients with cirrhosis: Revised consensus recommendations of the International Club of Ascites. *Journal of hepatology* (2015).
26. European Association for the Study of the, L. EASL clinical practice guidelines on the management of ascites, spontaneous bacterial peritonitis, and hepatorenal syndrome in cirrhosis. *Journal of hepatology* **53**, 397-417 (2010).
27. Moore, K.P. & Aithal, G.P. Guidelines on the management of ascites in cirrhosis. *Gut* **55 Suppl 6**, vi1-12 (2006).
28. Runyon, B.A. & Aasld. Introduction to the revised American Association for the Study of Liver Diseases Practice Guideline management of adult patients with ascites due to cirrhosis 2012. *Hepatology* **57**, 1651-1653 (2013).
29. Arvaniti, V.*, et al.* Infections in patients with cirrhosis increase mortality four-fold and should be used in determining prognosis. *Gastroenterology* **139**, 1246-1256, 1256 e1241-1245 (2010).
30. Bernardi, M., Caraceni, P., Navickis, R.J. & Wilkes, M.M. Albumin infusion in patients undergoing large-volume paracentesis: a meta-analysis of randomized trials. *Hepatology* **55**, 1172-1181 (2012).
31. Martin-Llahi, M.*, et al.* Terlipressin and albumin vs albumin in patients with cirrhosis and hepatorenal syndrome: a randomized study. *Gastroenterology* **134**, 1352-1359 (2008).
32. Fernandez, J.*, et al.* Effect of intravenous albumin on systemic and hepatic hemodynamics and vasoactive neurohormonal systems in patients with cirrhosis and spontaneous bacterial peritonitis. *Journal of hepatology* **41**, 384-390 (2004).
33. Sort P, N.M., Arroyo V, Aldeguer X, Planas R, Ruiz-del-Arbol L, Castells L, Vargas V, Soriano G, Guevara M, Ginès P, Rodés J. Effect of intravenous albumin on renal impairment and mortality in patients with cirrhosis and spontaneous bacterial peritonitis. *N Engl J Med.* **341**, 403-409 (1999).
34. Patel, A., Laffan, M.A., Waheed, U. & Brett, S.J. Randomised trials of human albumin for adults with sepsis: systematic review and meta-analysis with trial sequential analysis of all-cause mortality. *Bmj* **349**, g4561 (2014).
35. Caironi, P.*, et al.* Albumin replacement in patients with severe sepsis or septic shock. *The New England journal of medicine* **370**, 1412-1421 (2014).
36. Reviewers, C.I.G.A. Human albumin administration in critically ill patients: systematic review of randomised controlled trials. *Bmj* **317**, 235-240 (1998).
37. Thevenot, T.*, et al.* Effect of albumin in cirrhotic patients with infection other than spontaneous bacterial peritonitis. A randomized trial. *Journal of hepatology* **62**, 822-830 (2015).
38. Guevara, M.*, et al.* Albumin for bacterial infections other than spontaneous bacterial peritonitis in cirrhosis. A randomized, controlled study. *Journal of hepatology* **57**, 759-765 (2012).

Figure 1. Overview of ATTIRE RCT Study Protocol

For peer review only

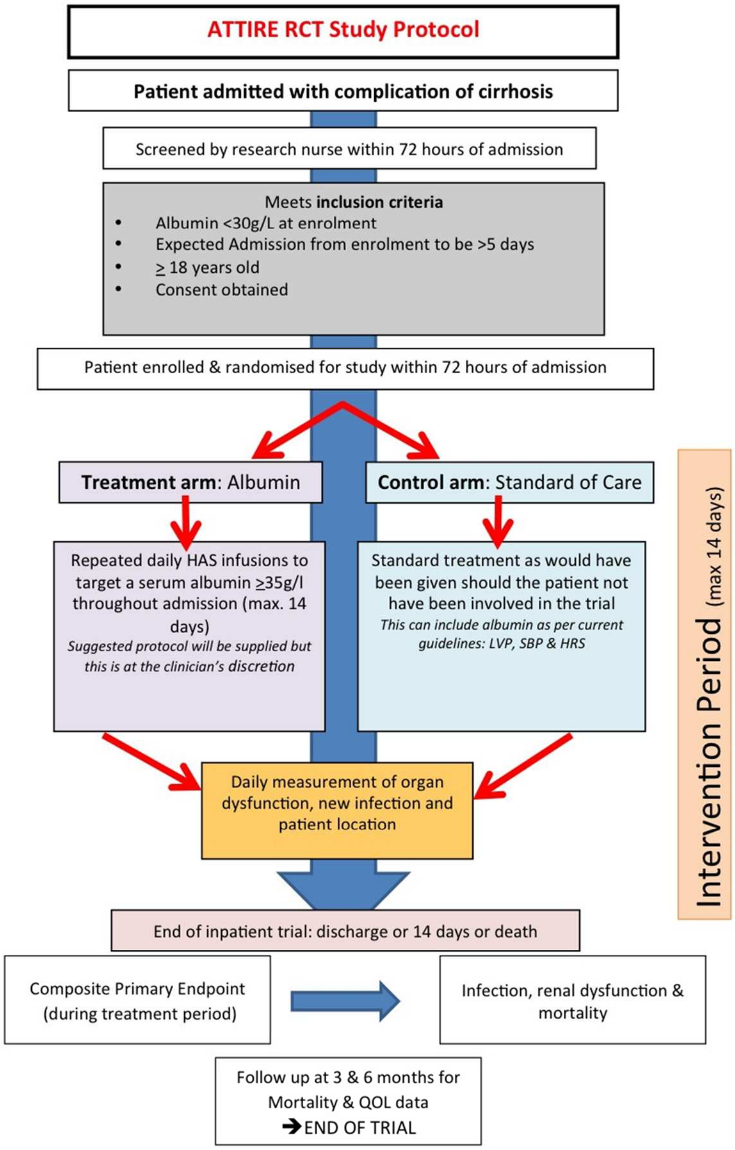

Figure 1. Overview of ATTIRE RCT Study Protocol

317x458mm (72 x 72 DPI)

Appendix 1 – Classification of infection

1. Spontaneous bacteraemia: positive blood cultures without a source of infection.
2. SBP: ascitic fluid polymorphonuclear cells >250 cells/mm^3
3. Lower respiratory tract infections: new pulmonary infiltrates in the presence of:
   i) at least one respiratory symptom (cough, sputum production, dyspnoea, pleuritic pain) with
   ii) at least one finding on auscultation (rales or crepitation) or one sign of infection (core body temperature >38°C or less than 36°C, shivering, or leukocyte count >10,000/mm3 or <4,000/mm3) in the absence of antibiotics.
4. Clostridium difficile Infection: diarrhoea with a positive C. difficile assay.
5. Bacterial entero-colitis: diarrhoea or dysentery with a positive stool culture for Salmonella, Shigella, Yersinia, Campylobacter,or pathogenic E. coli.
6. Soft-tissue/skin Infection: fever with cellulitis.
7. Urinary tract infection (UTI): urine white blood cell >15/high-power field with either positive urine gram stain or culture.
8. Intra-abdominal infections: diverticulitis, appendicitis, cholangitis, etc.
9. Other infections not covered above.
10. Fungal infections as a separate category.

For peer review only

Appendix 2 - Exploratory Objectives:

1. Effect of 20% HAS on the incidence, morbidity and mortality of the following infections:
    a. Bacterial
        i. Community acquired (diagnosed within 48 hours of admission in patients without previous hospitalisation in the last 6 months)
        ii. Nosocomial
        iii. Drug resistant pathogens
        iv. Culture negative sepsis (patients treated with antibiotics in the absence of microbiology evidence of infection)
        v. Clostridium Difficile infection
        vi. Source of infection (e.g. bacteraemia, respiratory, peritonitis, urine, skin etc.)
    b. Fungal
    c. Viral
2. Incidence, morbidity and mortality according to aetiology of Cirrhosis
3. Outcome for participants admitted to hospital for the first time with decompensated liver cirrhosis compared with participants who have previously been admitted to hospital with decompensated liver cirrhosis.
4. Requirement for ICU use of inotropes, vasopressors, hemofiltration and intubation and ventilation.
5. Analysis of samples from patients:
   a. Immune function studies using plasma samples – including but not limited to cell culture, qPCR, Albumin-ligand binding studies, western blot, flow cytometry and immunohistochemistry
   b. Plasma and urine lipidomic profile (in participants giving optional consent for urine samples)
6. Analysis of samples from patients providing additional optional consent (subject to further application for funding and ethical approval) :
   a. Stool microbiome analysis
   b. Genetic analysis of host response to infection
   c. Pathogen detection

For peer review only

Appendix 3: ATTIRE Patient Information Sheet (PIS)

ATTIRE: Stage 2

Albumin **T**o preven**T** **I**nfection in chronic live**R** failur**E**

The Problem

The most common cause of death in patients with liver disease is infection

➔ this is because in liver disease the immune system does not work well, especially when you are unwell in hospital.

What we know

Albumin (a protein found in your blood) is lower in patients with liver disease. Research looking at blood samples has suggested that increasing the albumin levels in the blood can potentially improve the immune system in patients with chronic liver disease.

What question is this study trying to answer?

1. Does albumin actually stop patients admitted to hospital with chronic liver disease from getting infections? (or help them fight them)

What additional treatment or investigations may occur if I take part?

If you decide to take part, you will be assigned to either the 'albumin group' or the 'standard of care group'. This allocation will be random.

1. In the 'standard of care group', you will receive exactly the same treatment as you would have done had you not been in the trial.
2. If in the 'albumin group' you will be given an intravenous infusion of albumin every day whilst you are in hospital **and** you will also receive all the other treatment that you would have done had you not been in the trial. Albumin is a very safe protein infusion that is already commonly used in liver patients who have kidney problems or ascites (fluid in the abdomen).
3. We will collect some blood samples (3 times) during your admission and if you are happy we will also collect urine (2) and stool sample (1). Where possible the blood will be taken when your other blood tests collection occurs to avoid any additional tests.

Throughout the trial you will be asked to complete some questionnaires; this will start when you enter the study and at 3 and 6 months after you are discharged from hospital. If you are happy we will collect one blood sample at either of these appointments.

What are the possible advantages?

You may not have any direct benefit from taking part in the study but you will be a part of a large study that will help to decide whether this is a beneficial treatment for patients with liver disease to help decrease infection.

What are the possible disadvantages?

You may be connected to a drip for 1-2 hours a day (this can be when other drip medication is given). The side effects of albumin are very rare (listed in detail in the following pages).

For peer review only - http://bmjopen.bmj.com/site/about/guidelines.xhtml

ATTIRE

Stage 2: Patient Information Sheet

Full study title:

Albumin To prevenT Infection in chronic liveR failurE.

A trial to investigate whether giving albumin to patients with advanced liver cirrhosis will reverse immune suppression and prevent infection.

Invitation to take part in research study

We would like to invite you to take part in a research trial. Before you decide we would like you to understand why the research is being done and what it involves. Please read this information sheet; one of the study team will answer any questions you may have. Talk to others about the study and ask us if there is anything that is not clear. Take time to decide whether or not you wish to take part.

The ATTIRE study is in two stages. The first stage included 80 patients and the second includes over 860 patients. They are very similar, but will look at slightly different outcomes.

This Patient Information Sheet is for Stage 2.

What is the purpose of the study?

Liver cirrhosis results in the liver tissue becoming scarred and developing lumps, these changes can prevent the liver from functioning properly. As the liver function worsens, symptoms such as fluid within the abdomen or legs, yellow skin, vomiting blood and confusion can develop. These are a result of advanced liver disease and are termed complications of cirrhosis. There are about 60,000 patient admissions to hospitals each year with such complications of liver cirrhosis. These patients are at higher risk of developing an infection during their hospital stay and this increase in risk is due to a weakened immune system. These infections can be very serious.

Giving fluid directly into the veins (intravenous) is known to be very important to help kidney function in patients with advanced liver disease. However, how much to give or indeed what is the best fluid to give is currently unknown. We have shown in laboratory testing, that albumin might be the best option as it can strengthen the immune system. However, it is unclear whether this will reduce the number of infections in hospital and that is what our trial wishes to investigate.

Albumin is found in the blood and is made in the liver. Patients with liver cirrhosis cannot make albumin as easily due to the damage to their liver. Albumin is already used to treat patients with liver cirrhosis and is known to be safe. In this stage of the study, patients will be given extra albumin to see if this helps their immune system to fight infections.

Why have I been chosen?

You are being asked to take part in this study as you have been admitted to hospital with complications of liver cirrhosis. Your doctor believes that you could be eligible to take part.

Do I have to take part?

It is up to you whether or not to take part in the study. If you decide not to take part, this will not affect the treatment you would normally receive. If you take part, you are free to withdraw at any time without giving a reason; this will not affect the care you receive.

What will happen to me if I take part?

For peer review only - http://bmjopen.bmj.com/site/about/guidelines.xhtml

If you take part in this study you will be either randomly allocated to the 'albumin group' or the 'standard of care group'.
If you are allocated to the 'albumin group', you will be given albumin solution as an intravenous fluid. Albumin has been shown to be safe in patients with liver disease. The amount of albumin that you are given will be decided by the amount that you already have in your blood. You will receive an infusion each day that you are in hospital, for a maximum of 14 days, until the levels in your blood have reached normal. You will also receive all of the other treatment that you would normally have had you not been in the study.
If you are in the 'standard of care group' you will have the treatment you would normally have, had you not been a part of the study, this may include albumin if you need it for another reason.
Regardless of which group you are in, you will have blood tests on most days while you are in hospital (as part of your normal care). At this time an additional sample (about 1 teaspoon) will be collected for up to 4 days during your time in the trial. You will also be asked to provide stool and urine samples while in the study, however this sample collection is optional and you can still take part in the study without providing these samples.
You also will be asked to complete some patient questionnaires during your time in the trial.
You will be contacted at 3 months and 6 months following your discharge from hospital to attend hospital visits, we will try to have these visits at the same time as your regular clinic appointments if possible.

What will I have to do?
If you agree to take part in the study you will be asked to sign a consent form. A member of the study team will then go through the initial assessments required for the study including collecting details of your medical history and any medication that you are currently taking. You will have tests (which will be part of your standard care) to look at your general health (e.g. heart rate and blood pressure), whether you have an infection, your liver function and the level of albumin already in your blood. Only if the level of albumin is below a certain level, will you be able to take part.

A research nurse will then come and record your blood tests and how well you are every day whilst you are in hospital. When you are discharged from hospital the research nurse will arrange to see you 3 months and 6 months after discharge to ask you some questions about how well you are feeling, this will be at the same time as your other clinic appointments if possible.

If you are a woman of child bearing potential, a pregnancy test will be carried out.

Starting Treatment

Once all of your tests have been completed and if you are still eligible to participate, a computer outside of the hospital will decide whether you will be in the 'albumin group' or the 'standard of care group', this is called randomisation. You will get exactly the same hospital care as you would have previously but with (or without) additional albumin infusions. This is so the trial investigators can see if there is an advantage, or disadvantage, of being given albumin.

The test for the amount of albumin in your blood will be used to work out how much albumin will be given, if you are in the 'albumin group', this will be given by a nurse or doctor. We expect the test to be performed anyway and so will still be done if you are in the 'standard of care group' as well. The albumin will be given directly into your blood through an intravenous cannula (a tube inserted into your vein to give fluid); the cannula will already be present as part of your standard care, an extra one will *not* be required. This will happen each day that you are on the study (a maximum of 14 days).

You will have blood tests on most days for liver function and to look for infections. These would normally be taken during your hospital stay. An extra blood sample (1 teaspoon) will be taken at the same time on three days throughout your admission while in the study and will be used to look at your immune system. You will also be asked to provide urine and stool samples whilst in the study, however these will be optional

For peer review only - http://bmjopen.bmj.com/site/about/guidelines.xhtml

samples. The stool samples and any samples left over will be stored for future research (for which further ethical committee review will be required).
If you do get an infection whilst in the study, you will be given the normal treatment for that type of infection that may include albumin. Taking part in the study will not affect the care that you normally get from your doctor.

End of study treatment
When you have taken part in the study for 14 days or your when your doctor feels you are ready to be discharged (if this is before 14 days of being on the study), you will stop your study treatments. This means that you will no longer be given albumin as part of the study or have any samples collected that are not part of your standard treatment. You will be asked to complete a questionnaire when you are discharged.
If you doctor feels that you still need albumin, you will continue to receive it.

The study team will then also contact you at 3 months and 6 months after you have been discharged from hospital, this will be at the same time as your other clinic appointments if possible. At these visits you will undergo tests that you would normally have as part of your standard of care and you will be asked to complete some questionnaires to see how you are feeling and what treatment you have had since being discharged from hospital. If you agree an extra blood sample (1 teaspoon, as above) will be taken at the same time as one of these appointments.

At the end of the study, if you agree, we will access information about you that is held by NHS digital, the Office of National Statistics (ONS) and other central NHS bodies regarding hospital admissions and mortality statistics for the six months following treatment. This is data that is collected as part of standard practice and will involve no additional involvement by you. The data, which is highly regulated, will be used to see if there are any long term effects of the treatment for you and the services that you may access.

When should I contact the study team?
You should contact a member of the study team or a doctor or nurse on your ward if you feel unwell at any time or have any incidents that affect your physical wellbeing. Contact details of the study team at your hospital and the Chief Investigator are available at the end of this leaflet.

What happens if I become pregnant?
All women of child-bearing potential will have a pregnancy test before entering the study. If you do become pregnant during treatment, you should inform your study team. It is not thought that albumin will affect pregnancy.

What are the alternative treatments if I don't take part?
If you do not take part in the study, standard treatment will be offered, which may include the use of albumin. Albumin is currently used for several reasons in patients with liver disease for example those who require extra fluid to support their kidneys, or to replace fluid that has been drained from the abdomen, or in those who develop infections.

What are the possible disadvantages and risks of taking part?
There are some possible risks and disadvantages that you should consider before taking part in this study:

- Wherever possible we will ensure that the blood tests needed for the study are taken at the same time as those needed for your routine care, but you may be asked to have additional samples taken if this is not possible. The risks of having a blood test include local bruising and discomfort.
- You may experience discomfort and bruising from fluid infusion.
- As the albumin is taken from human blood, in theory there is chance that you could get an infection (virus) from the person who has donated the blood. However there are no reports of patients getting viral infections from albumin that is made following European manufacturing standards. The albumin

For peer review only - http://bmjopen.bmj.com/site/about/guidelines.xhtml

given as part of the study is the same as that which would be given to you as part of your regular care by your doctor which you may have received as a treatment in the past.

- A cannula (very small plastic tube) will be used to give you the albumin. We would expect that you already will have one of these to give your other medications whilst in hospital and will use the same cannula. However if you don't have any medications or fluid that need to be given into the vein we will need to place a small cannula into the vein in order to give you the albumin.
- If in the albumin group you will be connected to an albumin infusion for around 1-2 hours a day. You can still move around the ward with this but will need to have a drip stand with you.

What are the possible benefits of taking part?
There may be no benefit to you from taking part, however, intravenous albumin may help your kidneys and raise blood pressure that can be low in patients with liver problems. Taking part may also provide additional information about patients with liver disease.

What are the side effects of any treatment received when taking part?
The side effects of albumin are rare but can include the following:

	Very Common	Common	Uncommon	Rare	Very Rare
Immune system disorders					Anaphylactic shock
Gastrointestinal disorders				Nausea	
Skin and subcutaneous tissue disorders				Flushing, skin rash	
General disorders and administration site conditions				Fever	

*__Very Common:__ in more than 1 in 10 patients treated; __Common:__ in less than 1 in 10, but more than 1 in 100 patients treated; __Uncommon:__ in less than 1 in 100, but more than 1 in 1000 patients; __Rare:__ in less than 1 in 1000, but more than 1 in 10000 patients treated; __Very rare:__ in less than 1 in 10000 patients treated, including isolated cases

Other side effects observed after placing Human Albumin on the market are: Hypersensitivity/Allergic reactions, Headache, Rapid heart beat, Abnormally low blood pressure, Breathlessness or breathing discomfort, Vomiting, Altered sense of taste, Hives, Itchiness, Chills, Heart attack, Irregular heart beat, Accumulation of fluid in the lung.

If any of the above reactions occur these will be treated as they would in normal standard care.

What if relevant new information becomes available?
Sometimes we get new information about the treatments being studied. If this happens, your study team will tell you about it and discuss how this may impact you. They will discuss whether you want to or should continue in the study.

What happens when the research study stops?
If you still require any treatment once the study stops, your doctor will prescribe the treatment most suitable for you.

Expenses and payments
You will not receive any payments for participating in this study.

For peer review only - http://bmjopen.bmj.com/site/about/guidelines.xhtml

What if something goes wrong?

If you have any concerns about the study, you should discuss them with your local study team first. You can also contact the Chief Investigator for the study, Dr Alastair O'Brien, on 020 7679 6851 who will be happy to discuss any of your concerns.

If you would like to discuss your concerns with someone not involved in the study, please contact your local Patient Advice and Liaison Service (PALS). Information on how to contact your local PALS can be found at the end of this information sheet.

Every care will be taken during this clinical study. However, in the unlikely event that you are injured by taking part, compensation may be available. If you suspect that the injury is the result of the Sponsor's (UCL) or the hospital's negligence then you may be able to claim compensation. After discussing it with your study team, please write to Dr Alastair O'Brien who is the Chief Investigator for the clinical study, about details of your claim. The Chief Investigator will then pass the claim to the Sponsor's Insurers, via the Sponsor's office. You may have to bear the costs of the legal action initially, and you should consult a lawyer about this. Contact details of the study team at your hospital and the Chief Investigator are available at the end of this leaflet.

You may also be able to claim compensation for injury caused by participation in this clinical study without the need to prove negligence on the part of University College London or another party. You should discuss this possibility with your study team in the same way as above.

Regardless of this, if you wish to complain, or have any concerns about any aspect of the way you have been approached or treated by members of staff or about any side effects (adverse events) you may have experienced due to your participation in the clinical study, the normal National Health Service complaints mechanisms are available to you. Please ask your study team if you would like more information on this. Details can also be obtained from the Department of Health website: http://www.dh.gov.uk.

Will my taking part in this study be kept confidential?

Yes, it will. We will request your approval to contact and inform your GP in the consent form about your participation in the study. We will send information relating to this research to the Comprehensive Clinical Trials Unit at University College London (UCL), where the study is being managed. Relevant information about you and all other patients in the study will be stored on password protected computers and in locked filing cabinets and will only be available to the staff working on the study. The information stored by the Comprehensive Clinical Trials Unit will contain a unique code, your initials, and month and year of birth. Your full name, address, contact details relevant medical history and relevant test results will be maintained at your local hospital. The information may be accessed by authorised personnel from UCL and the NHS hospital where you are being treated for auditing and monitoring purposes or by the regulatory authorities for inspection purposes to make sure the study is being carried out properly. Data collected during the study may be sent pseudo-anonymously (partially linked) to associated researchers in countries where the laws don't protect your privacy to the same extent as the law in the European Economic Area (EEA) but we will take all necessary steps to protect your privacy. Any data sent will only contain your unique code, your initials and month and year of birth. A data protection officer at UCL will be informed about any potential data transfer. You will be asked whether you agree to this when you sign the consent form.

If you withdraw consent from further study treatment, unless you object, your data and samples will remain on file and will be included in the final study analysis. In line with Good Clinical Practice guidelines, at the end of the study, your data will be securely archived for a minimum of 5 years. Arrangements for confidential destruction will then be made.

For peer review only - http://bmjopen.bmj.com/site/about/guidelines.xhtml

What will happen to the results of the research study?
If you choose to take part in the study, you will be given a unique trial identification code. All information collected from you for the study will be associated with this unique code, your initials, and month and year of birth. No other personal information will be sent to personnel outside your hospital and no one outside your hospital care team will be able to identify you from this information.

The blood, urine and stool samples collected for this study will be sent to the University College London, where they will be stored securely with your study specific identification number, initials, and month and year of birth. The blood and urine samples will be analysed at UCL to look at your immune system. The stool samples will be stored securely at UCL to be used for future research. Any future research carried out on the samples will be subject to further ethical committee review. It may be possible that the samples, or part of the samples, will be sent to countries outside the EEA where the laws for data protection differ from those in the UK. The urine and stool samples are optional samples and will not affect your participation in the study.

The Comprehensive Clinical Trials Unit will analyse the data collected on the patients in this stage of the study after all 866 patients have completed their treatment. The results of the study will be presented at a scientific conference and will be published in a scientific journal which will be accessible to the public. None of the research participants will be identified in any report or publication. Should you wish to see the results, or the publication, please ask your study team.

Who is conducting and funding the research?
This study is being managed by the Comprehensive Clinical Trials Unit at the University College London. Funding for the research has come from the Department of Health and Wellcome Trust.

Who has reviewed the study?
All research in the NHS is reviewed by a Research Ethics Committee and an agency of the Department of Health called the MHRA (the Medicines and Healthcare products Regulatory Agency). Both of these groups have confirmed that they are content for the study to go ahead and are regularly updated on its progress by the Comprehensive Clinical Trials Unit.

Who to contact for further information?
You are encouraged to ask any questions you wish, before, during or after your treatment. If you have any questions about the study, please speak to a member of the study team. If you wish to read the research on which this study is based, please ask your study team. If you require any further information or have any concerns while taking part in the study please contact one of the following people at your study site:

Doctor

Name *add name of PI* Tel. Number: *add Tel. number*

Study Nurse

Name *add name* Tel. Number: *add Tel. number*

PALS Add local PALS contact details

Or contact the Chief Investigator for the study:
Dr Alastair O'Brien at University College London, Tel. Number: 020 7 679 6851

Before you sign the informed consent form, you should ask questions about anything that you do not understand. The study staff will answer any questions before, during and after the study.

For peer review only - http://bmjopen.bmj.com/site/about/guidelines.xhtml

Thank you for taking the time to read this information sheet.

For peer review only

For peer review only - http://bmjopen.bmj.com/site/about/guidelines.xhtml

To be printed on hospital headed paper

ATTIRE

Stage 2: Patient Informed Consent Form

Albumin **T**o preven**T** **I**nfection in chronic live**R** failur**E** (ATTIRE)

PATIENT ID: [ ][ ][ ]-[ ][ ][ ]-[ ][ ] SITE: [ ][ ][ ]

MONTH AND YEAR OF BIRTH: [ ][ ][ ]-[ ][ ][ ][ ] PATIENT INITIALS: [ ][ ][ ]

MMM - YYYY

This Informed Consent Form is intended for consenting patients into Stage 2 of the ATTIRE study.

Please insert your **initials** in the boxes to confirm consent:

	**Initials**
1. I confirm that I have read and understood the Patient Information Sheet version 5.0 dated 15Jan2018 for the ATTIRE study and have had the opportunity to consider the information and ask questions, which have been answered to my satisfaction.	
2. I understand that my participation is voluntary and that I am free to withdraw at any time, without giving a reason and without my medical care or legal rights being affected.	
3. I understand that sections of my medical records may be looked at by properly authorised personnel involved in the running of the study or from regulatory authorities where it is relevant to my taking part in this study. I give permission for these individuals to have access to my records.	
4. I agree to provide blood samples.	
5. I agree to take part in the ATTIRE study.	
6. I agree to be contacted by the research team 3 and 6 months after my hospital discharge.	

For peer review only - http://bmjopen.bmj.com/site/about/guidelines.xhtml

Optional Consent:

The following are optional. Declining to any of the below will not prevent you from taking part in the trial. Please initial in the boxes to confirm consent:	Initials
7. I give permission for my study data to be sent to an associated researcher outside of the EEA.	
8. I agree to provide urine samples.	
9. I agree to provide a stool sample.	
10. I agree to my remaining samples, not used in this study, being used in future, ethically approved, research.	
11. I understand that the information held and maintained by NHS Digital, the Office of National Statistics and other central UK NHS bodies may be used to provide information about my health.	
12. I give permission for my GP to be informed about my participation.	

Patient/ Legal Representative's Name (score through as applicable)	Signature	Date
Person Taking Consent	Signature	Date
Witness (if applicable)	Signature	Date

For peer review only - http://bmjopen.bmj.com/site/about/guidelines.xhtml

Appendix 4

	***Screening/* Day 1 (Randomisation)** *(within 72hrs of admission)*	***Treatment Period*** *(Day 2- Day 14)*	***End of Treatment***	***Discharge***	***Follow up*** *(3 & 6 months +/- 1 month)[d]*
Eligibility screen	X				
Informed consent	X				
Medical history	X				
Blood tests and sample collection*	X	X	X		X
Clinical observations recorded	X	X	X		
Randomisation	X				
Dosing with HAS/Standard of care	X[b]	X			
Infection/new (change) in antibiotics	X	X	X		
Patient location recorded	X	X	X	X	X
Concomitant Medication	X	X	X	X	X
Pregnancy test (βhCG) (women of child bearing potential only)	X				
Survival		X	X		X
CSRI Questionnaire	X[c]				X
EQ-5D-5L Questionnaire	X			X	X

*Blood test include: FBC, U&ES, LFTs, CRP, INR, Serum Albumin concentrations. From patients who provide additional consent blood will be collected at randomisation (day 1) and on days 5 and 10 +/- 2 days. Urine sample will be collected on day 1 and day 5 +/- 2 days and A stool sample will be collected once during the patients treatment period. A blood sample from patients who consent will be taken at either the 3 or 6 month follow up visit.

[b] If randomised to receive the HAS treatment the volume of HAS given will be determined by the Serum Albumin results (see section 6.4.1.2)

[c] The CRSI should be completed before randomisation. If this is not possible, the questionnaire should be completed as soon as the patient is able, prior to discharge. The questionnaire should be completed in relation to the three months prior to admission – details of the current hospital admission should not be included.

[d] If the patient has been re-randomised into the study, before completing the follow up visits for the previous randomisation; the patient should not undergo the follow up activities relating to that randomisation The follow up visits should only be conducted for the current randomisation.

STANDARD PROTOCOL ITEMS: RECOMMENDATIONS FOR INTERVENTIONAL TRIALS

SPIRIT 2013 Checklist: Recommended items to address in a clinical trial protocol and related documents*

Section/item	Item No	Description	Addressed on page number
**Administrative information**			
Title	1	Descriptive title identifying the study design, population, interventions, and, if applicable, trial acronym	1
Trial registration	2a	Trial identifier and registry name. If not yet registered, name of intended registry	2
	2b	All items from the World Health Organization Trial Registration Data Set	2 (& registry)
Protocol version	3	Date and version identifier	2
Funding	4	Sources and types of financial, material, and other support	11
Roles and responsibilities	5a	Names, affiliations, and roles of protocol contributors	1, 12
	5b	Name and contact information for the trial sponsor	11 (& registry)
	5c	Role of study sponsor and funders, if any, in study design; collection, management, analysis, and interpretation of data; writing of the report; and the decision to submit the report for publication, including whether they will have ultimate authority over any of these activities	11
	5d	Composition, roles, and responsibilities of the coordinating centre, steering committee, endpoint adjudication committee, data management team, and other individuals or groups overseeing the trial, if applicable (see Item 21a for data monitoring committee)	11

For peer review only

For peer review only - http://bmjopen.bmj.com/site/about/guidelines.xhtml

Introduction

Background and rationale	6a	Description of research question and justification for undertaking the trial, including summary of relevant studies (published and unpublished) examining benefits and harms for each intervention	3, 4
	6b	Explanation for choice of comparators	8, 10
Objectives	7	Specific objectives or hypotheses	4
Trial design	8	Description of trial design including type of trial (eg, parallel group, crossover, factorial, single group), allocation ratio, and framework (eg, superiority, equivalence, noninferiority, exploratory)	4

Methods: Participants, interventions, and outcomes

Study setting	9	Description of study settings (eg, community clinic, academic hospital) and list of countries where data will be collected. Reference to where list of study sites can be obtained	5, 12
Eligibility criteria	10	Inclusion and exclusion criteria for participants. If applicable, eligibility criteria for study centres and individuals who will perform the interventions (eg, surgeons, psychotherapists)	4
Interventions	11a	Interventions for each group with sufficient detail to allow replication, including how and when they will be administered	6, 7
	11b	Criteria for discontinuing or modifying allocated interventions for a given trial participant (eg, drug dose change in response to harms, participant request, or improving/worsening disease)	6
	11c	Strategies to improve adherence to intervention protocols, and any procedures for monitoring adherence (eg, drug tablet return, laboratory tests)	6
	11d	Relevant concomitant care and interventions that are permitted or prohibited during the trial	7
Outcomes	12	Primary, secondary, and other outcomes, including the specific measurement variable (eg, systolic blood pressure), analysis metric (eg, change from baseline, final value, time to event), method of aggregation (eg, median, proportion), and time point for each outcome. Explanation of the clinical relevance of chosen efficacy and harm outcomes is strongly recommended	5
Participant timeline	13	Time schedule of enrolment, interventions (including any run-ins and washouts), assessments, and visits for participants. A schematic diagram is highly recommended (see Figure)	Fig 1

For peer review only - http://bmjopen.bmj.com/site/about/guidelines.xhtml

Sample size	14	Estimated number of participants needed to achieve study objectives and how it was determined, including clinical and statistical assumptions supporting any sample size calculations	7
Recruitment	15	Strategies for achieving adequate participant enrolment to reach target sample size	5
**Methods: Assignment of interventions (for controlled trials)**			
Allocation:			
Sequence generation	16a	Method of generating the allocation sequence (eg, computer-generated random numbers), and list of any factors for stratification. To reduce predictability of a random sequence, details of any planned restriction (eg, blocking) should be provided in a separate document that is unavailable to those who enrol participants or assign interventions	7
Allocation concealment mechanism	16b	Mechanism of implementing the allocation sequence (eg, central telephone; sequentially numbered, opaque, sealed envelopes), describing any steps to conceal the sequence until interventions are assigned	7
Implementation	16c	Who will generate the allocation sequence, who will enrol participants, and who will assign participants to interventions	7
Blinding (masking)	17a	Who will be blinded after assignment to interventions (eg, trial participants, care providers, outcome assessors, data analysts), and how	4, 7
	17b	If blinded, circumstances under which unblinding is permissible, and procedure for revealing a participant's allocated intervention during the trial	N/A
**Methods: Data collection, management, and analysis**			
Data collection methods	18a	Plans for assessment and collection of outcome, baseline, and other trial data, including any related processes to promote data quality (eg, duplicate measurements, training of assessors) and a description of study instruments (eg, questionnaires, laboratory tests) along with their reliability and validity, if known. Reference to where data collection forms can be found, if not in the protocol	5
	18b	Plans to promote participant retention and complete follow-up, including list of any outcome data to be collected for participants who discontinue or deviate from intervention protocols	5

For peer review only - http://bmjopen.bmj.com/site/about/guidelines.xhtml

Data management	19	Plans for data entry, coding, security, and storage, including any related processes to promote data quality (eg, double data entry; range checks for data values). Reference to where details of data management procedures can be found, if not in the protocol	online trial protocol
Statistical methods	20a	Statistical methods for analysing primary and secondary outcomes. Reference to where other details of the statistical analysis plan can be found, if not in the protocol	8
	20b	Methods for any additional analyses (eg, subgroup and adjusted analyses)	8
	20c	Definition of analysis population relating to protocol non-adherence (eg, as randomised analysis), and any statistical methods to handle missing data (eg, multiple imputation)	8
**Methods: Monitoring**			
Data monitoring	21a	Composition of data monitoring committee (DMC); summary of its role and reporting structure; statement of whether it is independent from the sponsor and competing interests; and reference to where further details about its charter can be found, if not in the protocol. Alternatively, an explanation of why a DMC is not needed	11, 12 (online trial protocol)
	21b	Description of any interim analyses and stopping guidelines, including who will have access to these interim results and make the final decision to terminate the trial	N/A
Harms	22	Plans for collecting, assessing, reporting, and managing solicited and spontaneously reported adverse events and other unintended effects of trial interventions or trial conduct	online trial protocol
Auditing	23	Frequency and procedures for auditing trial conduct, if any, and whether the process will be independent from investigators and the sponsor	online trial protocol
**Ethics and dissemination**			
Research ethics approval	24	Plans for seeking research ethics committee/institutional review board (REC/IRB) approval	10, 11
Protocol amendments	25	Plans for communicating important protocol modifications (eg, changes to eligibility criteria, outcomes, analyses) to relevant parties (eg, investigators, REC/IRBs, trial participants, trial registries, journals, regulators)	online trial protocol

For peer review only - http://bmjopen.bmj.com/site/about/guidelines.xhtml

Consent or assent	26a	Who will obtain informed consent or assent from potential trial participants or authorised surrogates, and how (see Item 32)	6
	26b	Additional consent provisions for collection and use of participant data and biological specimens in ancillary studies, if applicable	online trial protocol
Confidentiality	27	How personal information about potential and enrolled participants will be collected, shared, and maintained in order to protect confidentiality before, during, and after the trial	online trial protocol
Declaration of interests	28	Financial and other competing interests for principal investigators for the overall trial and each study site	12
Access to data	29	Statement of who will have access to the final trial dataset, and disclosure of contractual agreements that limit such access for investigators	online trial protocol
Ancillary and post-trial care	30	Provisions, if any, for ancillary and post-trial care, and for compensation to those who suffer harm from trial participation	online trial protocol
Dissemination policy	31a	Plans for investigators and sponsor to communicate trial results to participants, healthcare professionals, the public, and other relevant groups (eg, via publication, reporting in results databases, or other data sharing arrangements), including any publication restrictions	11
	31b	Authorship eligibility guidelines and any intended use of professional writers	12
	31c	Plans, if any, for granting public access to the full protocol, participant-level dataset, and statistical code	N/A
**Appendices**			
Informed consent materials	32	Model consent form and other related documentation given to participants and authorised surrogates	Supplementary info
Biological specimens	33	Plans for collection, laboratory evaluation, and storage of biological specimens for genetic or molecular analysis in the current trial and for future use in ancillary studies, if applicable	online trial protocol

*It is strongly recommended that this checklist be read in conjunction with the SPIRIT 2013 Explanation & Elaboration for important clarification on the items. Amendments to the protocol should be tracked and dated. The SPIRIT checklist is copyrighted by the SPIRIT Group under the Creative Commons "Attribution-NonCommercial-NoDerivs 3.0 Unported" license.

For peer review only - http://bmjopen.bmj.com/site/about/guidelines.xhtml
